# Flame Retardant Polypropylenes: A Review

**DOI:** 10.3390/polym12081701

**Published:** 2020-07-29

**Authors:** Farzad Seidi, Elnaz Movahedifar, Ghasem Naderi, Vahideh Akbari, Franck Ducos, Ramin Shamsi, Henri Vahabi, Mohammad Reza Saeb

**Affiliations:** 1 Provincial Key Lab of Pulp and Paper Science and Technology and Joint International Research Lab of Lignocellulosic Functional Materials, Nanjing Forestry University, Nanjing 210037, China; f_seidi@njfu.edu.cn; 2 Department of Polymer Processing, Iran Polymer and Petrochemical Institute, Tehran 14965/115, Iran; el.movahedifar@gmail.com (E.M.); g.naderi@ippi.ac.ir (G.N.); 3 Université de Lorraine, CentraleSupélec, LMOPS, F-57000 Metz, France; vahidehakbari1991@gmail.com; 4 Université de Lorraine, IUT de Moselle Est, IUTSGM, 57600 Forbach, France; franck.ducos@univ-lorraine.fr; 5 Research and Development Center, Marun Petrochemical Company, Mahshahr 63531 69311, Iran; ramin.shamsi44@gmail.com

**Keywords:** flame retardancy, polypropylene, *Flame Retardancy Index* (*FRI*), cone calorimetry, flame retardants

## Abstract

Polypropylene (PP) is a commodity plastic known for high rigidity and crystallinity, which is suitable for a wide range of applications. However, high flammability of PP has always been noticed by users as a constraint; therefore, a variety of additives has been examined to make PP flame-retardant. In this work, research papers on the flame retardancy of PP have been comprehensively reviewed, classified in terms of flame retardancy, and evaluated based on the universal dimensionless criterion of *Flame Retardancy Index* (*FRI*). The classification of additives of well-known families, i.e., phosphorus-based, nitrogen-based, mineral, carbon-based, bio-based, and hybrid flame retardants composed of two or more additives, was reflected in *FRI* mirror calculated from cone calorimetry data, whatever heat flux and sample thickness in a given series of samples. PP composites were categorized in terms of flame retardancy performance as *Poor*, *Good*, or *Excellent* cases. It also attempted to correlate other criteria like UL-94 and limiting oxygen index (LOI) with *FRI* values, giving a broad view of flame retardancy performance of PP composites. The collected data and the conclusions presented in this survey should help researchers working in the field to select the best additives among possibilities for making the PP sufficiently flame-retardant for advanced applications.

## 1. Introduction

Polymers are building blocks of advanced materials and systems, but their flammability has been a serious constraint in their usage in advanced applications [1,2,3]. Polypropylene (PP) is a commodity plastic widely used in a variety of applications, particularly in the form of composites in load-bearing uses due to its high rigidity and crystallinity [4]. By the end of 2020, the PP market size is expected to reach $112 billion, and it is estimated to reach $155 billion by 2026 [5,6]. Its global production was 56.0 million metric tons in 2018, and it is estimated to reach around 88.0 million metric tons by 2026. This growing demand reflects the importance of PP for applications where low density, hardness, high flexural modulus, and chemical resistance are needed [7,8]. Moreover, PP is a low-cost plastic capable of being processed with various methods, e.g., extrusion, thermoforming, and injection molding [9,10]. Therefore, a huge number of PP products, including fibers, films, sheets, textiles, pipes, and profiles, have been developed and used in the automotive, electrical and electronic, packaging, and construction industries [11,12,13,14]. On the other hand, due to the inherent flammability, the use of flame-retardant additives in PP is necessary to minimize the risk of fire [15]. Different types of flame retardants have been used in PP including minerals, phosphorus-based, nitrogen-based, and intumescent [16,17,18]. It was recognized that additive selection plays a crucial role in achieving acceptable flame retardancy [19], where the type, the size, and the loading percentage of flame retardants control the fire behavior of PP matrix.

A diversity of additives are used in PP to make it flame retardant. There is a need for a comprehensive survey to classify PP composites in terms of flame retardancy. In the present paper, several families of flame retardants examined in PP have been identified and categorized to evaluate their flame retardancy performance in terms of *Flame Retardancy Index* (*FRI*) [19,20]. *FRI* is a universal dimensionless index that takes into account well-known parameters obtained from cone calorimeter test (peak of heat release rate (pHRR), the total heat release (THR), and the time to ignition (TTI)). *FRI* can be simply calculated using Equation (1):

(1) $$\mathrm{FRI}=\frac{{\left[\mathrm{THR}\;\times \left(\frac{\mathrm{pHRR}}{\mathrm{TTI}}\right)\right]}_{\mathrm{Neat}\;\mathrm{Polymer}}}{{\left[\mathrm{THR}\;\times \left(\frac{\mathrm{pHRR}}{\mathrm{TTI}}\right)\right]}_{\mathrm{Composite}}}$$

Basically, the use of *FRI* makes it possible to semi-qualitatively classify polymer composites by labeling them as *Poor*, *Good*, or *Excellent* flame retardancy performance and thus enables evaluation of the efficiency of the incorporated flame retardant (FR). There has always been a need for fast-tracking and classifying polymers for their flame retardant performance. The use of *FRI* made possible classifying polymers and polymer composites in terms of flame retardancy in a simple manner. For *FRI* values below 10^0^ obtained by the use of Equation (1), we have the case (namely *Poor*) where the addition of FR adversely affects flame retardancy of polymer. When *FRI* takes values in the range of 10^0^–10^1^, we name it *Good* flame retardancy performance, such that addition of FR enhances the resistance of polymer against fire. For *FRI* values above 10^1^, which is rare in practical cases, we have an *Excellent* case, where FR significantly improves flame retardancy. It is worth mentioning that some important parameters of testing such as irradiance and sample thickness as well as sample weight can be neglected due to the fact that, in the *FRI* formula, the parameters related to the neat polymer are divided by those of polymer/FR composite. Thus, the dimensionless value obtained can be used as a reliable measure of the efficiency of FR in polymer. In this survey, the data from the literature were extracted first, and five families of flame retardants that served as PP were considered including phosphorus-based, nitrogen-based, mineral, carbon-based, and bio-based flame retardants, and hybrid cases composed of the aforementioned five categories were distinguished. The main aim of the present survey is to give the readers a broad view of FR systems used in PP via *FRI* classification method. Certainly, this classification is not a precise and unique data set for FR selection for PP, but it can be considered as a database to compare different systems. The focus of this work was particularly placed on the reports in which cone calorimetry test was carried out. However, some other parameters such as smoke quantity or the percentage of FR elements (phosphorus, nitrogen, …) were not systematically given in this research paper due to the lack of data, which could lead to unreliable judgments. For some papers, limiting oxygen index (LOI) and UL-94 data were also available, which were used in finding possible correlations between the *FRI* variation and other criteria.

## 2. Phosphorus-Based Flame Retardants

Various types of phosphorus-based flame retardants have been incorporated into PP to make it flame-retardant [21,22,23]. Table 1 reviews the names and the percentages of these flame retardants incorporated into PP. Moreover, the values obtained from cone calorimetry such as the peak of heat release rate (pHRR), the total heat release (THR), and the time to ignition (TTI) are summarized in this Table. The *FRI* value, calculated from cone calorimetry parameters, as well as the LOI and UL-94 values, are also presented in Table 1. In some cases, if LOI and/or UL-94 values were not available, the sign “―” was used.

**Figure 1:**
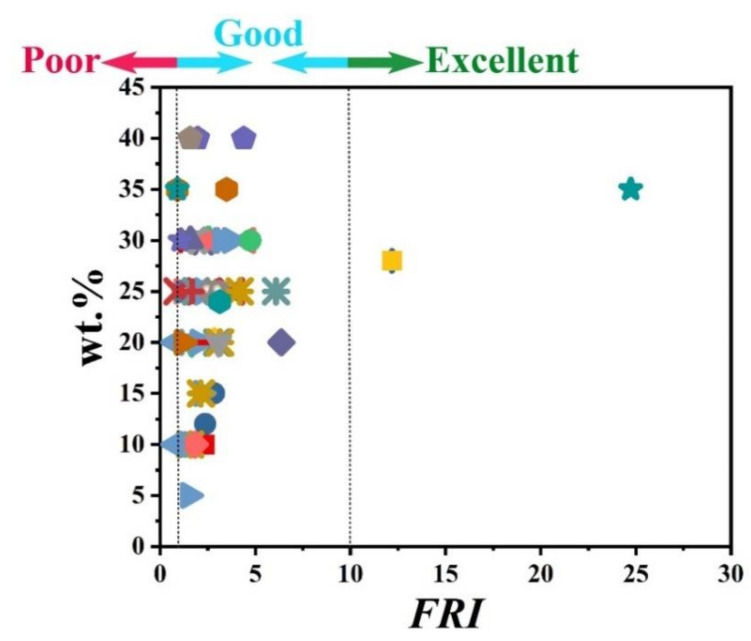
*Flame Retardancy Index* (*FRI*) values as a function of phosphorus flame retardant (FR) type and content. Symbols are indicative of different types of phosphorus flame retardant used. Here: ■ APP-10 [24], 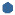 APP-12, APP-15 [25], 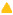 APP-20 [26], 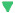 APP-20 [27], 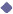 APP-20 [28], 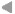 APP-20 [23], 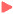 APP-25 [29], 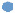 APP-25 [30], 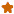 APP-25 [31], 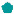 APP-25 [32], 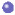 APP-25 [33], 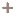 APP-25 [34], 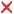 APP-25, m-APP-25 [35], 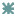 APP-25, mc-APP-25 [36], 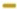 m-APP-25 [37], 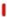 APP-30 [38], 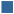 APP-30 [39], 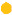 APP-30 [40], 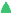 mc-APP-30 [41], 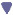 APP-30, mc-APP-30 [42], 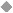 APP-30, mc-APP-30 [43], 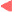 APP-30, mc-APP-30 [44], 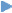 APP-30, mc-APP-5, mc-APP-10, mc-APP-15, mc-APP-20, mc-APP-25, mc-APP-30 [45], 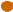 APP-35, m-APP-35 [46], 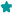 APP-35, m-APP-35 [47], 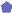 APP-40, mc-APP-40 [48], 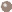 APP-25, P-CA-25 [49], 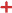 APP-25 [50], 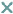 APP-IFR-20 [51], 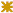 P-IFR-10, P-IFR-15, P-IFR-20, P-IFR-25 [52], 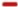 P-IFR-20 [53], 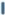 P-IFR-28 [54], 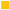 P-IFR-28 [55], 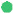 PN-IFR-30 [56], 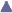 P-FR-30 [57], 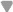 P-FR-20 [58], 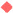 DOPO-10 [15], 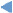 mc-BDP-10, mc-BDP-20 [59], 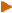 OP-20 [60], 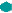 AHP-24 [61], 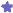 ALPi-30 [62], 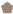 PEPA-40 [63].

The information provided in Table 1 clearly reveals that APP is quite frequently used as a major phosphorus flame retardant in PP matrix. The percentage of incorporation of phosphorus flame retardants was variable from 10 to 40 wt.%. Figure 1 displays the *FRI* as a function of wt.% phosphorus-based FR in PP systems. The name/type of each phosphorus flame retardant is provided in the caption of Figure 1. Three formulations reached the *Excellent* level of flame retardancy, which is quite rare among such data pool. The loading percentage of FR in these formulations varied from 28 to 35 wt.%. Many additives were modified APP and modified phosphorus-nitrogen flame retardants. It can also be speculated that a high loading percentage cannot necessarily guarantee the *Excellent* level of flame retardancy; besides, the type of phosphorus FR is also an important parameter. Figure 1 also reveals that the majority of points are located in the *Good* zone of *FRI*. Therefore, it can be concluded that phosphorus-based flame retardants have quite satisfactorily reinforced PP against flame.

**Figure 2:**
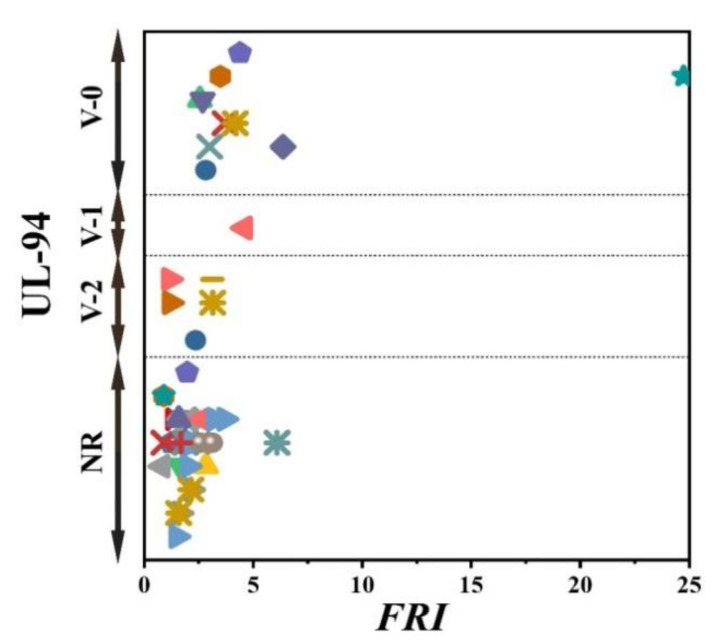
*FRI* values versus UL-94 test results. Symbols are indicative of different types of phosphorus flame retardant (FR) used. The vertical intervals in each category, i.e., V-0, V-1, V-2, and NR, are schematically representative of the amount of additive used. For example, two data distinguished by different symbols having the same or very close *FRI* values (horizontal quantity) in a given category (e.g., V-1) may have different vertical quantities, e.g., both reveal V-1 behavior in the UL-94 test, but the upper contains more FR in Polypropylene (PP).

**Figure 3:**
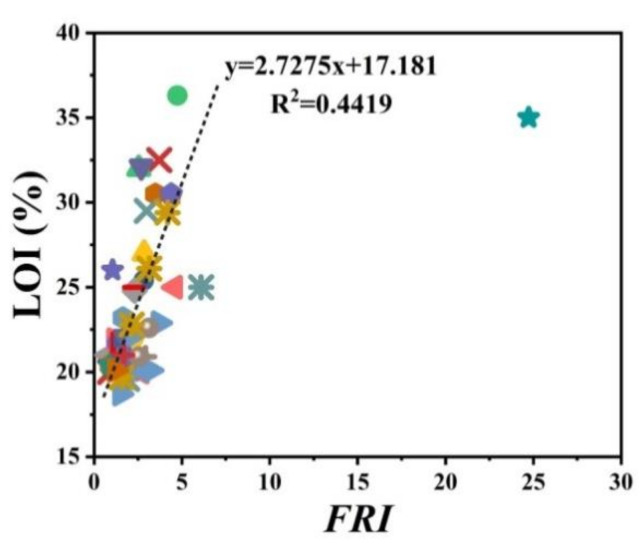
*FRI* values of PP as a function of limiting oxygen index (LOI) test results. Symbols are indicative of different types of phosphorus flame retardant used.

There has always been interest in exploring possible correlations between the data collected from different analyses made on PP materials. Figure 2 shows the flame retardancy performance of phosphorus FR-containing PP in terms of *FRI* versus the corresponding UL-94 test outcomes. From these data, it is evident that no specified correlation exists between the qualitative results collected from UL-94 and the quantitative ones obtained in cone calorimeter measurements. However, in the case of LOI results, Figure 3 suggests a meaningful relationship can be drawn among data achieved from the calculated *FRI* and the LOI test results. The LOI value for pure PP is around 17; however, it is increased by addition of flame retardant up to 36, more than a two-fold rise.

## 3. Nitrogen-Based Flame Retardants

Nitrogen-based flame retardants have also been used in PP to make it resistant against fire. Table 2 gives the names and the percentages of incorporation of these flame retardants, where the data were obtained in cone calorimetry (pHRR, THR, and TTI), *FRI* calculated from cone calorimetry parameters, as well as LOI and UL-94 values. Some of the nitrogen-based FRs listed in Table 2 also contain a phosphorus element. However, the percentage of nitrogen is more important, and therefore these FRs are listed in this Table.

**Figure 4:**
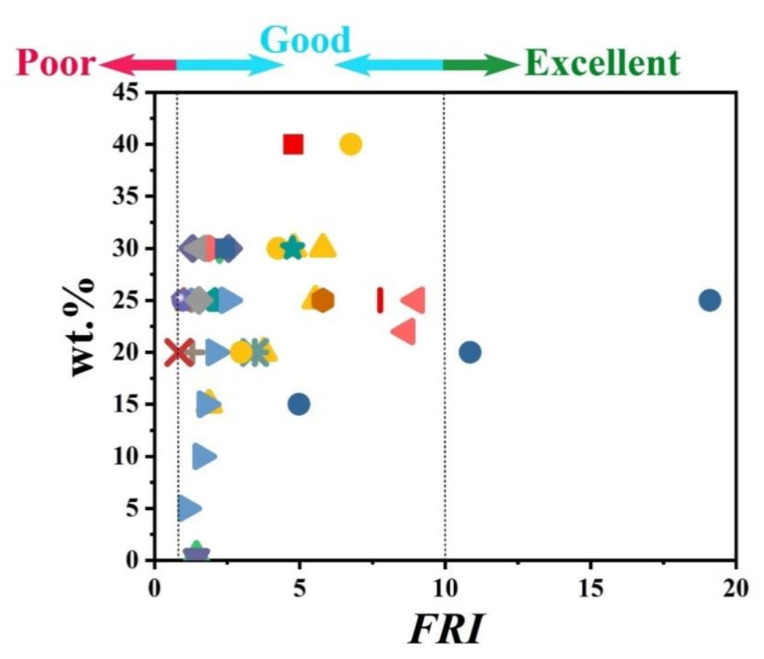
*FRI* values as a function of nitrogen FR type and content. Symbols are indicative of different types of nitrogen flame retardant used. Here: ■ MP-40 [63], 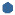 MPPK-15, MPPK-20, MPPK-25 [64], 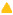 MTP-15, MTP-20, MTP-25, MTP-30, m-MTP-30 [65], 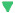 MPyP-30 [57], 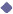 MPyP-30, TA-CFA-30 [66], 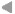 TA-CFA-30 [39], 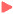 TA-CFA-30 [40], 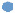 TA-CFA-25 [37], 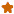 TA-CFA-25 [32], 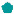 TA-CA-ZnO-25 [29], 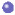 TA-CA-25 [67], 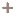 TA-CA-20 [27], 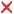 TA-CA-20 [23], 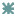 TA-IFR-20 [68], 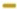 TA-IFR-25 [31], 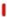 PI-FR-25 [69], 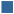 PI-IFR-30 [70], 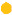 PI-IFR-20, PI-IFR-30, PI-IFR-40 [71], 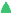 NOR116-0.5 [72], 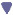 NOR116-0.3 [73], 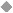 PPU-CA-25 [50], 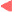 N-FR-22, N-FR-25 [74], 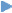 N-IFR-5, N-IFR-10, N-IFR-15, N-IFR-20, N-IFR-25 [75], 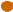 N-IFR-25 [76], 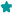 PN-IFR-30 [56].

To give a bright view of the variation trend, Figure 4 illustrates the *FRI* values as a function of wt.% of nitrogen-based flame retardants incorporated into the PP. The percentage of incorporation was changed from 15 to 40 wt.%. Of note, all points are located in the *Good* zone of *FRI*, except two points remarked as *Excellent*. These two points correspond to a kaolinite additive modified with nitrogen and phosphorus agents. A very noticeable point to be considered is that increasing the amount of diallyldimethylammonium (nominated with the 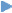 symbol in Figure 4) from 5 to 25 has no serious effect on the value of *FRI*, so that they are aligned vertically around *FRI* values between 1.0 and 2.5. Overall, like what happened to other polymers [77,78], combinatorial flame retardants may be the solution to flammability reduction of PP materials.

**Figure 5:**
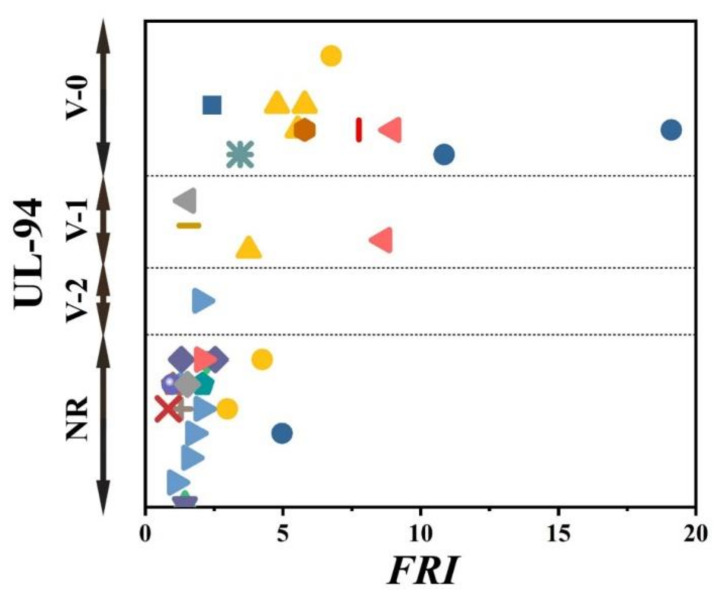
*FRI* values versus UL-94 test results. Symbols are indicative of different types of nitrogen flame retardant (FR) used. The vertical intervals in each category, i.e., V-0, V-1, V-2, and NR, are schematically representative of the amount of additive used. For example, two data distinguished by different symbols having the same or very close *FRI* values (horizontal quantity) in a given category (e.g., V-1), may have different vertical quantities; e.g., both reveal V-1 behavior in UL-94 test, but the upper contains more FR in PP.

**Figure 6:**
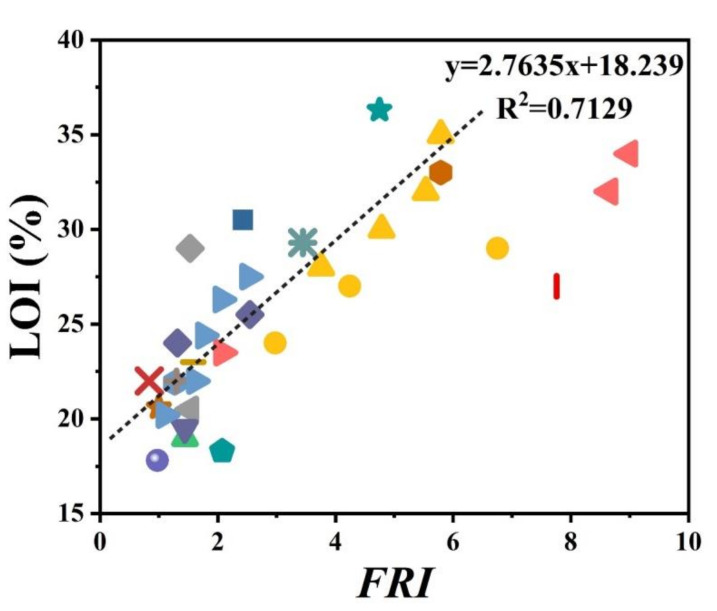
*FRI* values of PP as a function of LOI test results. Symbols are indicative of different types of nitrogen flame retardant used.

Figure 5 patterns UL-94 results as a function of *FRI* for nitrogen-based flame retardant in PP. It can be observed that even at small quantities of *FRI*, V0 in UL-94 was achieved. The diversity of data in Figure 5 can be taken as a signature of sensitivity of UL-94 to *FRI*. Figure 6 shows LOI values as a function of *FRI*. There is a quite reasonable correlation between the LOI and *FRI* values, up to *FRI* value of 6.

## 4. Mineral-Based Flame Retardants

Mineral additives have been widely used in polymers for their acceptable cost and properties [79]. Mineral-based flame retardants including clays are widely used in PP due to their low cost and acceptable thermal resistance. In this family, the most used flame retardants in volume were aluminum trihydroxide (ATH) and magnesium dihydroxide (MDH). However, due to their low efficiency, a high percentage of loading was necessary for achieving an acceptable level of flame retardancy of polymers. The name and the percentage of the used mineral-based flame retardants in PP are listed in Table 3. Cone calorimetry data, *FRI*, LOI, and UL-94 values are also given so as to make possible a detailed view on the status of flame retardant efficiency of PP materials.

**Figure 7:**
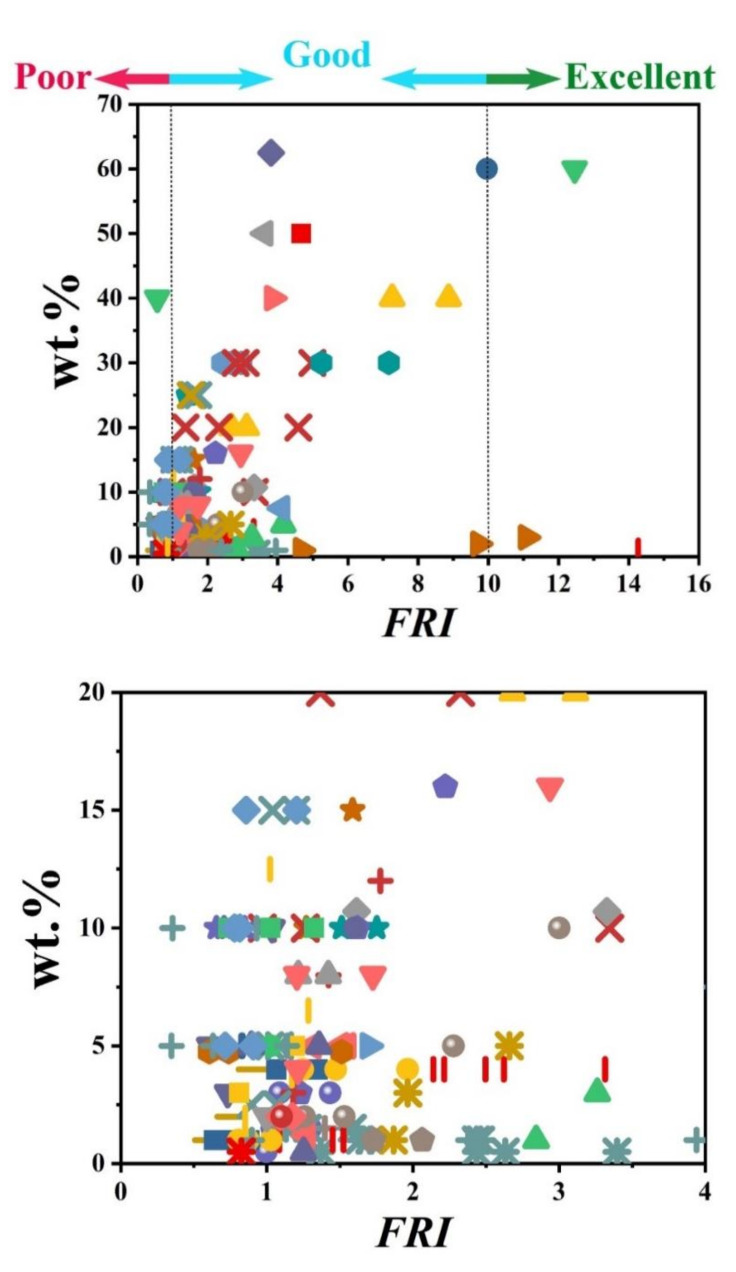

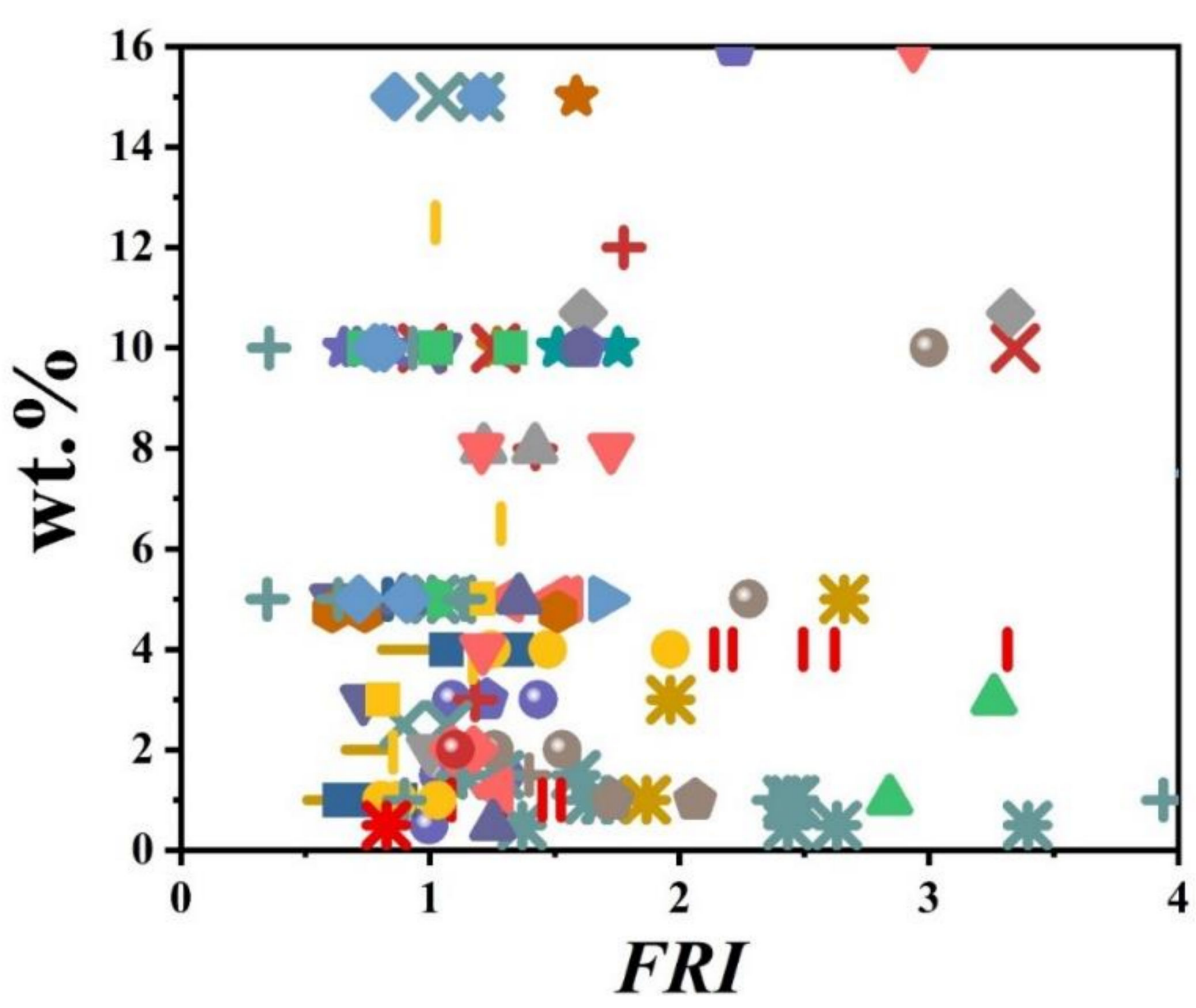
*FRI* values as a function of the mineral FR type and content from close-up and long-shot views. Symbols are indicative of different types of mineral flame retardant used. The diversity and abundance of data were reasons why such different scales were provided for detection of behavior of PP against flame. Here: ■ ATH-50 [80], 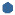 ATH-60 [81], 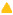 ATH-20, ATH-40, MDH-20, MDH-40 [82], 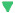 MDH-40, MDH-60 [82], 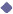 MDH-62.5 [83], 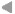 MDH-50 [84], 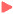 MDH-40 [85], 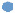 MDH-30, m-MDH-30, m-MDH-30 [86], 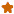 MDH-10, MDH-15 [87], 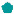 Kaol-25 [64], 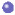 Kaol-0.5, Kaol-1.5, Kaol-3, m-Kaol-0.5, m-Kaol-1.5, m-Kaol-3 [88], 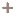 Kaol-1.5, m-Kaol-1.5 [89], 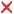 Kaol-10, Kaol-20, Kaol-30, m-Kaol-10, m-Kaol-20, m-Kaol-30, TC-10, TC-20, TC-30 [90], 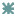 LDH-0.5, LDH-1, LDH-1.5, m-LDH-0.5, m-LDH-1, m-LDH-1.5, LDH-0.5, LDH-1, LDH-1.5, m-LDH-0.5, m-LDH-1, m-LDH-1.5 [91], 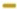 A-LDH-1, A-LDH-2, B-LDH-1, B-LDH-2, B-LDH-4, C-LDH-1, C-LDH-2, C-LDH-4, D-LDH-1, D-LDH-2, D-LDH-4, E-LDH-1, E-LDH-2, E-LDH-4 [92], 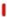 A-LDH-1, A-LDH-4, B-LDH-1, B-LDH-4, C-LDH-1, C-LDH-4, D-LDH-1, D-LDH-4, E-LDH-1, E-LDH-4 [92], 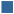 A-LDH-1, A-LDH-4, B-LDH-1, B-LDH-4, C-LDH-1, C-LDH-4, D-LDH-1, D-LDH-4, E-LDH-1, E-LDH-4 [92], 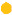 A-LDH-1, A-LDH-4, C-LDH-1, C-LDH-4, E-LDH-1, E-LDH-4 [92], 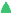 m-LDH-1, m-LDH-3, m-LDH-5 [93], 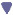 m-LDH-3, m-LDH-5, m-LDH-10 [94], 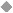 LDH-10.7, m-LDH-10.7 [95], 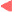 alkyl-NH_4_Cl-1.2, MMT-5, H-MMT-5, m-MMT-5 [96], 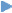 m-MMT-5 [96], 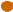 m-MMT-4.75, m-MMT-4.75, m-MMT-4.75 [97], 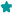 MMT-10, m-MMT-10 [24], 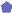 m-MMT-3, m-MMT-10, m-MMT-16 [98], 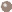 MMT-2, m-MMT-2, m-MMT-5, m-MMT-10 [99], 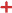 m-MMT-3, m-MMT-8, m-MMT-12 [100], 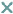 m-MMT-2.5, m-MMT-5, m-MMT-15, m-MMT-25, m-MMT-2.5, m-MMT-5, m-MMT-15, m-MMT-25 [101], 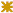 m-MMT-1, m-MMT-3, m-MMT-5 [102], 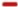 Nf-5, m-BT-5 [103], 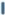 Nf-5, m-BT-5 [103], 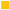 C20A-1, C20A-3, C20A-5 [104], 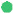 C15A-5 [60], 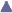 C20A-5, C20A-5, TiO_2_-0.5 [105], 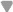 Al_2_O_3_-2 [106], 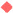 NiFeO-2, CoFeO-2 [107], 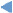 Ni_2_O_3_-7.5 [108], 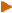 Nmm-cat-1, Nmm-cat-2, Nmm-cat-3 [109], 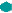 MOSw-30, m-MOSw-30 [110], 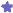 MnO-10, Mn_2_O_3_-10, MnC_2_O_4_-10 [111], 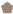 Znacac-1, Cracac-1 [53], 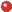 ZrPP-2 [112], 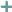 S4SQH-1, S4SQH-5, S4SQH-10, m-S4SQH-1, m-S4SQH-5, m-S4SQH-1, m-S4SQH-5, m-S4SQH-10 [113], 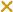 Si-FR-25 [30], 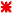 SEP-0.5, m-SEP-0.5 [25], 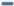 SEP-5, m-SEP-5 [87], 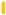 me-POSS-1.95, me-POSS-6.5, ph-POSS-3.75, ph-POSS-12.5 [114], 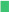 T8-POSS-10, Al-POSS-10, Zn-POSS-10 [115], 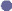 SA-10 [5], 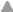 HNT-8, HNT-W-8 [116], 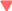 HNT-8, HNT-W-4, HNT-W-8, HNT-W-16 [116], 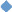 HNT-5, HNT-10, HNT-15, m-HNT-5, m-HNT-10, m-HNT-15 [117].

Figure 7 visualizes the variation of *FRI* value as a function of flame retardant loading in PP systems (for the convenience of readers, two figures are added for giving a better zoom on data points). This figure clearly shows that even at low loading percentages, it is possible to achieve a relatively high *FRI* value depending on the type of mineral. There is no denying that some parameters such as the state of dispersion and size of particles are important factors affecting the flame retardant properties.

**Figure 8:**
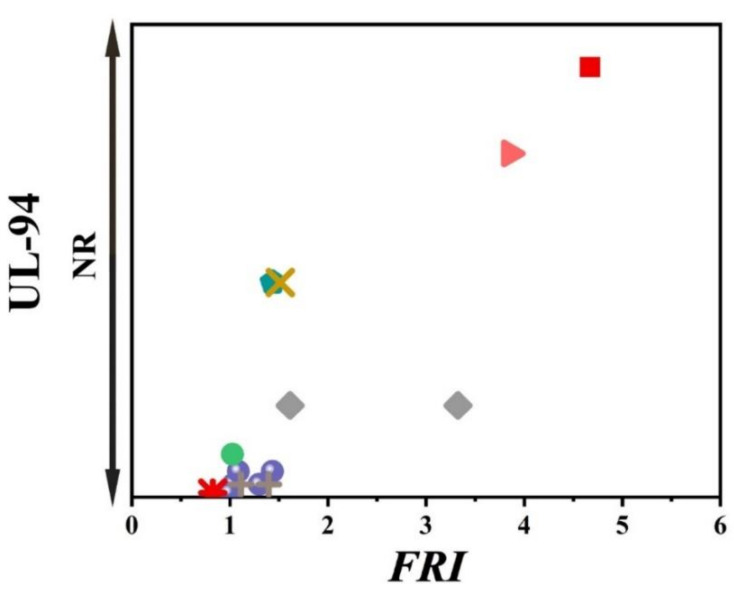
*FRI* values versus UL-94 test results. Symbols are indicative of different types of mineral flame retardant (FR) used. The vertical intervals in each category, i.e., V-0, V-1, V-2, and NR, are schematically representative of the amount of additive used. For example, two data distinguished by different symbols having the same or very close *FRI* values (horizontal quantity) in a given category (e.g., V-1), may have different vertical quantities, e.g., both reveal V-1 behavior in UL-94 test, but the upper contains more FR in PP.

**Figure 9:**
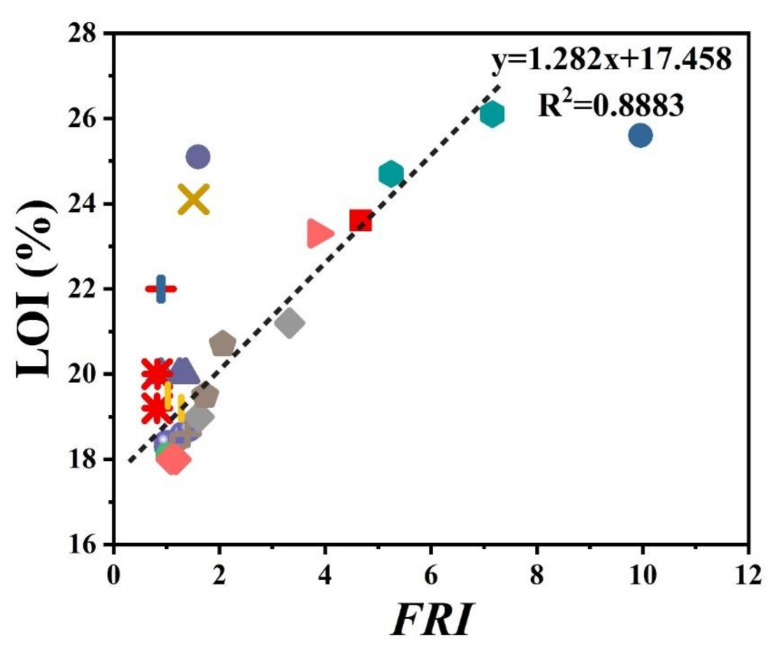
*FRI* values of PP as a function of LOI test results. Symbols are indicative of different types of mineral flame retardant used.

Unfortunately, the number of papers in which cone calorimetry, UL-94, and LOI values were studied was indeed limited, but the ones available are used plotting Figure 8. It should be noted that no formulation among studied ones is rated at V0. In conclusion, it is quite difficult to find a correlation between quantitative and qualitative parameters based on such a tiny set of data. In regard to the relationship between LOI and *FRI*, a meaningful trend can still be seen in Figure 9.

## 5. Carbon-Based Flame Retardants

Carbon-based additives have been widely used in developing polymer composites and nanocomposites [118,119,120,121]. However, due to expense and limited interaction with PP, a few works based on carbon-based flame retardants have been reported on flame-retardant PP materials. Table 4 summarizes all information available on the flame-retardant PP materials containing carbon-based additives.

**Figure 10:**
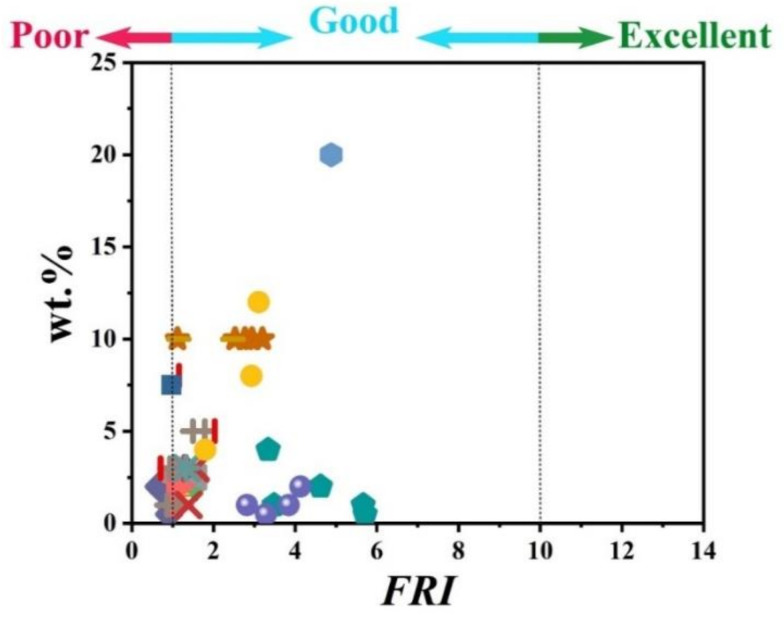
*FRI* values as a function of carbonaceous FR type and content. Symbols are indicative of different types of carbonaceous flame retardant used. Here: ■ GN-2, m-GN-2 [106], 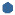 m–rGNO-2, m–rGNO-2 [122], 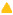 rGNO-2, m-rGNO-2, m-rGNO-2 [123], 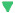 rGNO-2, m-rGNO-2 [112], 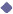 GNO-2, m-GNO-0.5, m-GNO-1, m-GNO-2 [124], 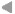 GN-2.5, GN-NiO-2.5, GN-NiCe_x_O_y_-2.5 [125], 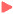 rGNO-2, m-rGNO-1, m-rGNO-2, m-rGNO-3 [126], 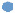 m-rGNO-20 [58], 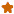 EG(ES 350 F5)-10, EG(ES 700 F5)-10, EG(Nyagraph FP)-10, EG(TEG 315)-10, EG(Nyagraph KP251)-10 [127], 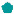 CNT-1, m-CNT-0.5, m-CNT-1, m-CNT-2, m-CNT-4 [128], 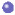 CNT-1, m-CNT-0.5, m-CNT-1, m-CNT-2 [129], 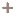 MWCNT-1, MWCNT-3, MWCNT-5, m-MWCNT-1, m-MWCNT-3, m-MWCNT-5 [130], 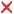 MWCNT-1, MWCNT-3 [131], 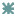 MWCNT-3 [132], 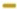 MWCNT-10, CF-10 [133], 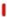 CF-3, CF-8, CB-5 [134], 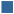 AC-7.5 [108], 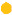 VGCNF-4, VGCNF-8, VGCNF-12 [6].

**Figure 11:**
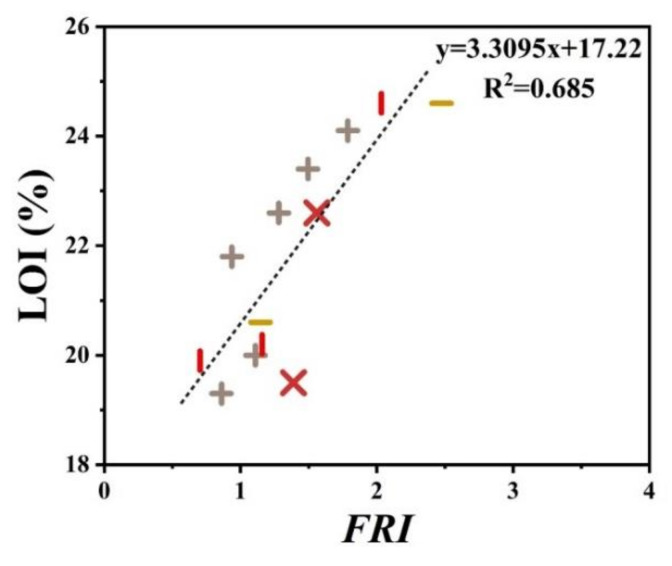
*FRI* values of PP as a function of LOI test results. Symbols are indicative of different types of carbon-based flame retardant used.

Figure 10 shows that with low loading percentage (1 wt.%) of carbon nanotubes, it is possible to achieve the *Good FRI*. No data were available for UL-94 tests. Comparison between Figure 7 and Figure 10 also suggests that low-cost minerals were used at higher loadings, while carbon-based additives were used almost at loadings below 10 wt.%. A limited number of data have also been reported on LOI values. These points are plotted as a function of *FRI* in Figure 11, where a good correlation can be established between *FRI* and LOI values. Deeper understanding of the mechanism behind such correlation requires a detailed view of the origin of tests as well as the chemical structure of additives and possible interaction between the PP and additives.

## 6. Bio-Based Flame Retardants

**Figure 12:**
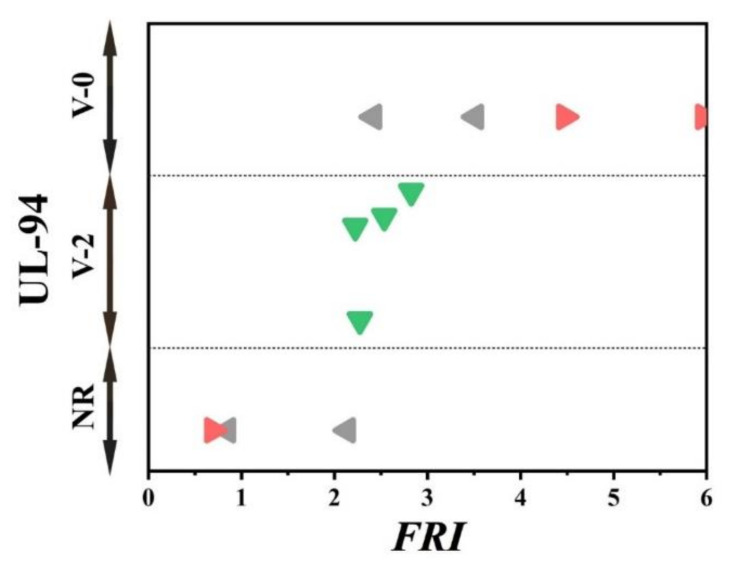
*FRI* values versus UL-94 test results. Symbols are indicative of different types of bio-based flame retardant (FR) used. The vertical intervals in each category, i.e., V-0, V-1, V-2, and NR, are schematically representative of the amount of additive used. For example, two data distinguished by different symbols having the same or very close *FRI* values (horizontal quantity) in a given category (e.g., V-1) may have different vertical quantities, e.g., both reveal V-1 behavior in UL-94 test, but the upper contains more FR in PP.

**Figure 13:**
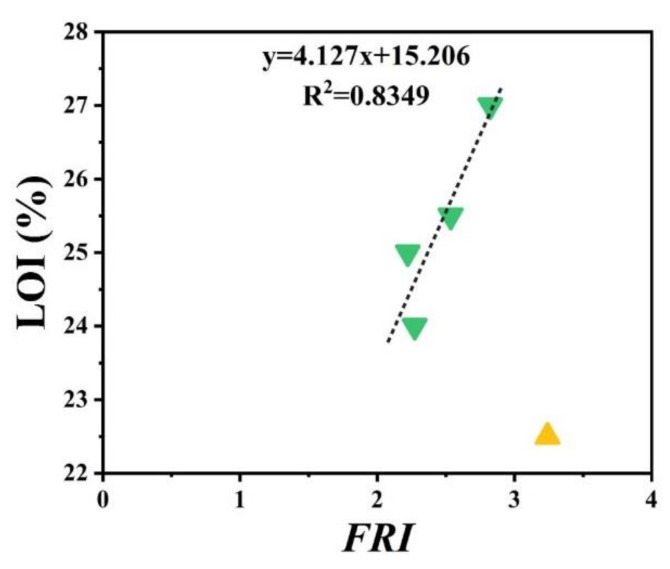
*FRI* values of PP as a function of LOI test results. Symbols are indicative of different types of bio-based flame retardant used. The green triangles are related to a mixture of phytic acid and piperazine-based FR. The increase of LOI is directly related to the percentage of FR loading, 15, 18, 20, and 25 wt.%.

In recent years, due to sustainability issues, the use of bio-based additives has also been investigated in PP. However, the number of research papers is limited on this subject. Table 5 gives the name and loading percentage of these bio-based FR. The obtained results from cone calorimetry, LOI, and UL-94 tests are also listed in Table 5. Figure 12 and Figure 13 display UL-94 and LOI results as a function of *FRI* for bio-based flame retardant in PP, respectively. 

**Figure 14:**
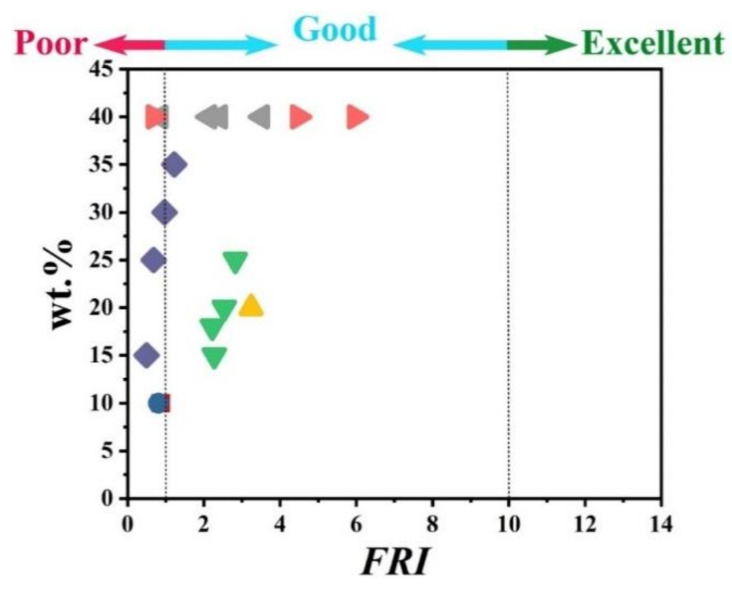
*FRI* values as a function of bio-based FR type and content. Symbols are indicative of different types of bio-based flame retardant used. Here: ■ CD-10 [135], 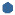 HAandCD-FR-10 [15], 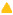 m-lig-20 [136], 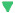 PHPI-FR-15, PHPI-FR-18, PHPI-FR-20, PHPI-FR-25 [137], 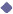 BC-15, BC-25, BC-30, BC-35 [138], 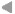 Wool-40, m-wool-40, m-wool-40, m-CF-40 [28], 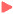 CF-40, m-CF-40, m-CF-40 [139].

*FRI* values are plotted as a function of loading percentage of bio-based FR in Figure 14. It can be observed that a high quantity of bio-based FR, 40 wt.% is needed to achieve *FRI* equal to 6.

## 7. Combination of Flame Retardants

As observed in previous sections, using an additive alone can to a limited extent improve flame-retardant properties of PP. Combination of flame retardants is a strategy to improve further the flame retardancy via synergism between various flame retardants [140,141,142]. Moreover, the quantity of the used flame retardant can be reduced in polymer so as to prevent mechanical properties deterioration. Different combinative additive systems were considered in PP. The corresponded data are collected and summarized in Table 6. The third column gives the ratio between flame retardants.

**Figure 15:**
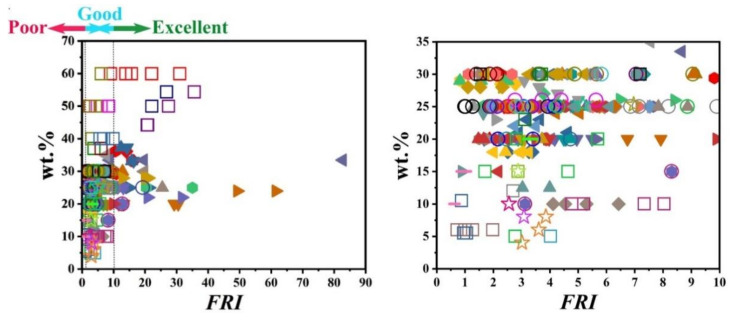
*FRI* values as a function of combinatorial FR additives and their content in PP in long-shot (left-hand figure) and close-up (right-hand figure) views. Symbols are indicative of different types of combinatorial flame retardant used. Here: ▲ APP-13.2/PER-6.8 [68], ▼ APP-16.7/PER-8.3 [143], 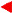 APP-18.7/PER-6.3 [67], 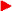 APP-22.5/PER-7.5, mc-(APP-22.5&PER-7.5) [144], 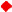 APP-10.5/PER-9.8/MEL-9.1, APP-15.3/PER-9.3/MEL-8.8, APP-19.1/PER-8.9/MEL-8.2 [145], 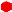 APP-10.5/PER-9.8/MEL-9.1, APP-15.3/PER-9.3/MEL-8.8, APP-19.1/PER-8.9/MEL-8.2 [145], 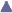 APP-15.3/PER-9.3/MEL-8.6, APP-14.3/PER-8.7/MEL-8.1/MDH-6.2 [146], 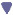 APP-15.3/PER-9.3/MEL-8.6, APP-14.3/PER-8.7/MEL-8.1/MDH-6.2 [146], 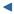 APP-12/PER-4/MEL-4/C20A-1, APP-12/PER-4/MEL-4/C20A-3, APP&MMT-12/PER-4/MEL-4/C20A-1, APP&MMT-12/PER-4/MEL-4/C20A-3 [104] 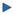 m-APP-16.6/DPER-4.2/MEL-4.2, m-APP-16/DPER-4/MEL-4/SEP-1 [147], 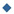 APP-13.5/PER-4.5, APP-12.75/PER-4.25/MF-1, APP-12.75/PER-4.25/MFA-1 [148], 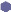 APP-22.5/PER-7.5 [149], 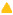 APP-12.7/PER-5.3, APP-12/PER-5/TA-FR-1 [150], 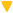 APP-16.67/PER-8.33, APP-16.33/PER-8.17/NOR116-0.5 [72], 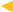 APP-13.5/PER-4.5, APP-12.75/PER-4.25/G-bases-1, APP-12.75/PER-4.25/U-bases-1 [151], 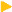 APP-17.2/DPER-7.8, m-APP-17.2/DPER-7.8, APP-16.2/DPER-7.8/ATH-1 [152], 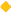 APP-21.4/PER-7.1, APP-20.3/PER-6.8/Kaol-1.4 [70], 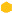 APP-21.75/PER-7.25, APP-19.5/PER-6.5/MMT-3, APP-19.5/PER-6.5/m-MMT-3 [153], 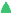 APP-21.75/PER-7.25, APP-19.5/PER-6.5/MMT-3, APP-19.5/PER-6.5/m-MMT-3, APP-19.5/PER-6.5/m-MMT-3 [154], 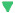 APP-18.75/PER-6.25, APP-18/PER-6/LDH-1, APP-18/PER-6/m-LDH-1 [155], 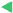 APP-18.75/PER-6.25, APP-18/PER-6/m-SiR-1, APP-18/PER-6/m-SiR-1, APP-16.5/PER-5.5/m-SiR-3 [156], 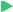 m-APP-16.7/DPER-8.3, m-APP-16.7/DPER-8.3/Z-1, m-APP-16.7/DPER-8.3/Z-1/MWCNT-0.1 [157], 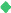 APP-18.75/PER-6.25, APP-18.75/PER-6.25/ALL-2 [158], 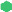 APP-16.7/PER-8.3, APP-15/PER-7.5/MAO-2.5, APP-15/PER-7.5/Zn-MAO-2.5 [159], 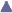 APP-16.7/PER-8.3, APP-16/PER-8/m-SEP-1, APP-15.3/PER-7.7/m-SEP-2, APP-14.7/PER-7.3/m-SEP-3, APP-14/PER-7/m-SEP-4, APP-13.3/PER-6.7/m-SEP-5 [160], 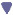 APP-15/PER-5, APP-14.25/PER-4.75/OP-POSS-1, APP-14.25/PER-4.75/A-POSS-1, APP-14.25/PER-4.75/OA-POSS-1, APP-14.25/PER-4.75/TS-POSS-1 [161], 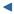 APP-20/PER-10¸ APP-19/PER-9.5/T-RS-5, APP-19/PER-9.5/CV-5, APP-19/PER-9.5/CR-5 [162], 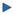 APP-15/PER-5, APP-14.25/PER-4.75/ZnB-1, APP-14.25/PER-4.75/BPO_4_-1, APP-14.25/PER-4.75/Bsi-1, APP-14.25/PER-4.75/LaB-1 [163], 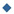 APP-18.75/PER-6.25, APP-17.25/PER-5.75/NiFeO-2, APP-17.25/PER-5.75/CoFeO-2 [107], 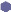 APP-16.67/PER-8.33, APP-15.33/PER-7.67/Ni_12_P_5_-2, APP-15.33/PER-7.67/Co_2_P-2, APP-15.33/PER-7.67/Cu_3_P-2 [164], 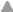 APP-18.75/PER-6.25, APP-18/PER-6/ZHS-1 [165], 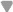 APP-18.75/PER-6.25, APP-18.75/PER-6.25/MnAc-1, APP-18.75/PER-6.25/MnAc-2, APP-18.75/PER-6.25/MnAc-3, APP-18.75/PER-6.25/MnAc-4 [166], 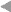 APP-21/DPER-7/m-SA-7 [167], 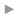 APP-15.4/PEPA-7.6, APP-15.4/PEPA-7.6/NOR116-2, APP-15.4/PEPA-7.6/ZrP-2, APP-15.4/PEPA-7.6/m-ZrP-2 [168], 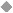 MCAPP-16.7/PEPA-8.3, MCAPP-15.7/PEPA-7.8/Kaol-1.5, MCAPP-15.7/PEPA-7.8/m-Kaol-1.5 [169], 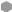 MCAPP-16.7/PEPA-8.3, MCAPP-15.7/PEPA-7.8/Kaol-1.5, MCAPP-15.7/PEPA-7.8/m-Kaol-1.5 [170], 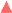 MCAPP-16.7/PEPA-8.3, MCAPP-15.7/PEPA-7.8/Kaol-1.5, MCAPP-15.7/PEPA-7.8/Kaol nanoroll-1.5 [171], 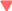 MCAPP-16.7/PEPA-8.3, MCAPP-15.7/PEPA-7.8/Kaol-1.5, MCAPP-15.7/PEPA-7.8/m-Kaol-1.5 [89], 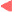 mc-APP-16.7/PEPA-8.3, mc-APP-15.7/PEPA-7.8/Kaol-1.5, mc-APP-15.7/PEPA-7.8/HNT-1.5, mc-APP-15.7/PEPA-7.8/Kaol-1.35/HNT-0.15 [172], 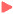 mc-APP-16.7/PEPA-8.3, mc-APP-15.7/PEPA-7.8/Kaol-1.5, mc-APP-15.7/PEPA-7.8/HSA-A-1.5, mc-APP-15.7/PEPA-7.8/HSA-P-1.5, mc-APP-15.7/PEPA-7.8/HSA-A-La-1.5, mc-APP-15.7/PEPA-7.8/HSA-P-La-1.5 [173], 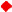 APP-12.5/P-CA-12.5 [49], 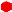 APP-28/PhZ-FR-2, APP-26/PhZ-FR-4, APP-24/PhZ-FR-6, APP-22/PhZ-FR-8 [38], 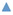 mc-APP-22.5/THEIC-7.5 [41], 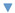 APP-16.67/PPU-CA-8.33, APP-12.5/PPU-CA-12.5 [50], 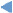 APP-17.6/TA-CFA-4.4, m-APP-17.6/TA-CFA-4.4 [95], 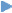 APP-16.7/TA-CFA-8.3 [32], 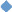 APP-20/TA-CFA-5, m-APP-20/TA-CFA-5 [37], 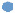 APP-20/TA-CFA-10, APP-22.5/TA-CFA-7.5, APP-24/TA-CFA-6 [40], 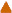 APP-15/TA-CFA-15, APP-20/TA-CFA-10, APP-22.5/TA-CFA-7.5, APP-24/TA-CFA-6 [39], 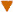 APP-15/TA-CFA-5, APP-14.63/TA-CFA-4.87/m-MMT-0.5, APP-14.25/TA-CFA-4.75/m-MMT-1, APP-13.87/TA-CFA-4.63/m-MMT-1.5, APP-13.5/TA-CFA-4.5/m-MMT-2, APP-12.75/TA-CFA-4.25/m-MMT-3 [174], 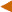 APP-18.24/TA-CFA-4.56/SiO_2_-1.2, APP-15.66/TA-CFA-3.91/AHP-3.4/SiO_2_-1.03 [61], 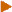 APP-18.24/TA-CFA-4.56/SiO_2_-1.2, APP-18.24/TA-CFA-4.56/SiO_2_-1.2 [175], 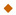 APP-20/TA-CFA-5, APP-19.6/TA-CFA-4.9/rGNO-0.5, APP-19.2/TA-CFA-4.8/rGNO-1, APP-18.4/TA-CFA-4.6/rGNO-2 [176], 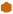 m-APP-20/TA-CFA-5, m-APP-19.6/TA-CFA-4.9/rGNO-0.5, m-APP-19.2/TA-CFA-4.8/rGNO-1, m-APP-18.4/TA-CFA-4.6/rGNO-2, m-APP-17.6/TA-CFA-4.9/m-APP@rGNO-2.5, m-APP-15.2/m-APP@rGNO-5/TA-CFA-4.8, m-APP-10.4/m-APP@rGNO-10/TA-CFA-4.6 [177], 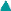 APP-10/TA-CA-10 [23], 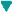 APP-13.33/TA-CA-6.67, APP-15/TA-CA-5 [27], 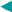 APP-16.7/TA-CA-8.3 [67], 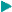 APP-18.75/TA-CA-6.25, mc-APP-18.75/TA-CA-6.25 [36], 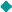 APP-13.33/TA-CA-6.67 [149], 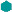 APP-20/TA-CA-10, APP-20/Homo-TA-CA-10 [178], ▲ APP-15/TA-CA-15, APP-24/TA-CA-6 [179], ▼ APP-16.7/TA-CA-8.3, APP-16.5/TA-CA-8.2/NOR116-0.3 [73], ◄ APP-14.7/TA-CA-5.3, APP-14/TA-CA-5/m-MMT-1, APP-12.5/TA-CA-4.5/m-MMT-3 [180], ► APP-16.72/TA-CFA-4.18/SiO_2_-1.1, APP-16.72/TA-CFA-4.18/SiO_2_-1.1 [181], ♦ APP-16.7/TA-CA-ZnO-8.3 [29], 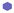 APP&TA-IFR-10, APP&TA-IFR-15, APP&TA-IFR-20 [182], 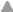 APP-12.5/TA-IFR-12.5, APP-16.67/TA-IFR-8.33, APP-18.75/TA-IFR-6.25 [31], 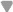 APP-20/PI-TA-CA-5, APP-20/PI-TA-CA-5 [33], 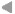 APP-22.5/PI-IFR-7.5, APP-16.4/PI-IFR-8.2/TA-CFA-5.4 [70], 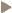 APP-15/ATH-15, mc-(APP-15&ATH-15) [183], 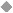 APP-8/MMT-2, APP-6/MMT-4, APP-8/m-MMT-2, APP-6/m-MMT-4 [24], 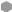 APP-15/Nf-5, APP-15/m-BT-5 [103], 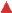 APP-15/Nf-5, APP-15/m-BT-5 [103], 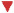 APP-15/C15A-5, OP-15/C15A-5 [60], 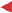 APP-12/C20A-5/PER-4/MEL-4, APP&MMT-12/C20A-5/PER-4/MEL-4, APP&MMT-6/C20A-5/PER-2/MEL-2, APP&MMT-9/C20A-5/PER-3/MEL-3 [104], 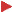 APP-19/m-LDH-1, APP-18/m-LDH-2, APP-17/m-LDH-3 [26], 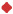 APP-IFR-18/LDH-2, APP-IFR-16/LDH-4, APP-IFR-18/LDH-2, APP-IFR-16/LDH-4 [51], 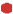 APP-12.5/Si-FR-12.5, APP-15/Si-FR-10, APP-13.8/Si-FR-9.2, APP-16.67/Si-FR-8.33, APP-18.75/Si-FR-6.25 [30], 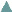 APP-12/SEP-0.5, APP-12/m-SEP-0.5 [25], 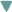 APP-22.8/SiO_2_-1.2 [175], 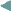 APP-22/CB-3, APP-20/CB-5, APP-18/CB-7 [34], 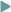 APP-7.5/CD-7.5 [135], 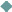 P-CA-24/MEL-6 [184], 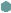 P-FR-15/MPyP-15 [57], 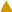 P-FR-20/GNO-2 [58], 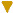 P-IFR-19/Znacac-1, P-IFR-19/Cracac-1 [53], 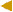 PN-IFR-29.5/m-MMT-0.5, PN-IFR-29/m-MMT-1, PN-IFR-28.5/m-MMT-1.5, PN-IFR-28/m-MMT-2, PN-IFR-27.5/m-MMT-2.5, PN-IFR-27/m-MMT-3, PN-IFR-26/m-MMT-4, PN-IFR-25/m-MMT-5 [56], 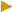 P-IFR-25.5/m-MMT-2.5, P-IFR-25.5/m-LDH-2.5, P-IFR-25.5/A-POSS-2.5, P-IFR-25.5/MWCNT-2.5 [55], 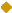 P-IFR-26.5/MWCNT-1.5, P-IFR-25.5/MWCNT-2.5, P-IFR-24.5/MWCNT-3.5 [54], 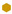 PA-16/EDA-9 [139], 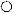 PPU-CA-12.5/APP-12.5, PPU-CA-16.67/APP-8.33, PPU-CA-18.75/APP-6.25, PPU-CA-20/APP-5 [50], 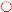 MP-15/PER-10, MP-15/PER-9/Kaol-1, MP-15/PER-8/Kaol-2 [64], 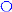 MP-12.3/PER-7.7, MP-15.4/PER-9.6, MP-11.7/PER-7.3/m-MMT-1, MP-14.7/PER-9.3/m-MMT-1 [185], 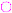 MMP-16.64/PER-9.36, MMP-16/PER-9/La_2_O_3_-1, MMP-15.36/PER-8.64/La_2_O_3_-2, MMP-14.72/PER-8.28/La_2_O_3_-3 [186], 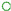 MPP-22.5/DPER-7.5, MPP-15/EG-10/DPER-5 [187], 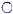 MATMP-16.7/PER-8.3 [143], 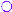 MPyP-18.75/PER-6.25, MPyP-17.25/PER-5.75/m-CD-2 [188], 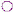 MPyP-15/P-FR-15 [57], 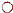 MPyP-22.5/TA-CFA-7.5, MPyP-15/TA-CFA-15, TA-CFA-22.5/MPyP-7.5 [66], 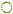 TA-CFA-15/APP-15, TA-CFA-20/APP-10 [39], 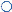 TA-CA-10/APP-10 [23], 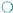 TA-CA-13.33/APP-6.67 [27], 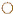 TA-CA-20/APP-10, TA-CA-15/APP-15 [179], 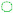 TA-IFR-12.5/APP-12.5, TA-IFR-16.67/APP-8.33 [31], 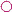 TA&APP-IFR-10, TA&APP-IFR-15, TA&APP-IFR-20 [182], 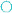 PI-IFR-20/APP-10 [70], 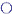 N-FR-24.5/SiO_2_-0.5, N-FR-22/SiO_2_-3 [74], 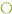 N-IFR-20/PA6-5 [75], 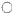 N-IFR-24.5/HGM-0.5, N-IFR-24/HGM-1, N-IFR-23/HGM-2, N-IFR-22/HGM-3 [76], 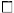 ATH-15/APP-15, mc-(ATH-15&APP-15) [183], 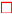 ATH-55/GB-5, ATH-50/GB-10, ATH-47/GB-10/m-ZrP-3, ATH-44/GB-10/m-ZrP-6, ATH-41/GB-10/m-ZrP-9 [81], 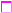 ATH-49/m-MMT-1, ATH-47/m-MMT-3, ATH-45/m-MMT-5 [80], 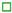 ATH-20/m-MMT-3, ATH-20/m-MMT-10, ATH-20/m-MMT-17 [82], 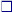 MDH-16.6/APP-12.7/PER-7.7/MEL-7.2, MDH-25/APP-11.5/PER-7/MEL-6.5, MDH-31.8/APP-10.4/PER-6.3/MEL-5.9 [146], 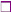 MDH-16.6/APP-12.7/PER-7.7/MEL-7.2, MDH-25/APP-11.5/PER-7/MEL-6.5, MDH-31.8/APP-10.4/PER-6.3/MEL-5.9 [146], 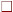 MDH-20/m-MMT-17 [82], 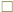 MDH-30/m-MMT-10, MDH-40/m-MMT-10, MDH-50/m-MMT-10 [82], 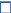 MDH-39/m-MMT-1, MDH-37/m-MMT-3, MDH-35/m-MMT-5 [85], 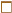 MDH-45/Cobalt chelate-5, MDH-40/Cobalt chelate-10, MDH-35/Cobalt chelate-15, MDH-30/Cobalt chelate-20 [84], 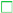 m-MMT-2.5/SEP-2.5, MDH-10/m-SEP-5, MDH-15/m-SEP-5, MDH-10/m-MMT-2.5/SEP-2.5, MDH-15/m-MMT-2.5/SEP-2.5 [87], 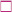 MMT-6/APP-4, MMT-8/APP-2, m-MMT-6/APP-4, m-MMT-8/APP-2 [24], 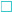 Si-FR-12.5/APP-12.5 [30], 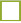 Ni_2_O_3_-7.5/AC-7.5 [108], 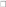 SEP-10/MWCNT-2 [189], 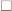 C30B-3/ACPB-3, C30B-3/BUPB-3, C30B-3/MEPB-3, C30B-3/PBPA-3 [191], 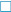 Si-3/SnCl_2_-2 [190], 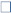 C20A-5/TiO_2_-0.5, m-C20A-5/TiO_2_-0.5, m-C20A-10/TiO_2_-0.5 [105], 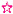 CF-5/MWCNT-5 [133], 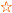 CB-3/MWCNT-1, CB-5/MWCNT-1, CB-5/MWCNT-3 [131], 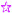 CB-5/CF-3 [134], 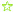 AC-7.5/Ni_2_O_3_-7.5 [108], 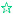 PER-10.4/MEL-9.7/APP-5.2 [145], 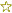 PER-10.4/MEL-9.7/APP-5.2 [145], 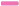 CD-7/TEP-3, CD-10/TEP-5, CD-7.5/APP-7.5 [135], 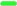 m-lig-18/Ni(Ac)_2_-2, m-lig-18/Co- (Ac)_2_-2, m-lig-18/Zn(Ac)_2_-2 [136].

Figure 15 displays the performance of different combinatorial additive systems used for PP. It can be clearly observed from the left-hand side figure that cases with *FRI* values above 10 (*Excellent* zone) are more frequent compared to all previous cases in which only one additive was used. More interestingly, the combination of additives appeared a useful strategy where very high *FRI* values (event more than 50) took place at intermediate loadings (25–30 wt.%). For achieving a high *FRI* value, the combination of several types of flame retardants is needed, for example, phosphorus, intumescent, and mineral flame retardants [150] or phosphorus, nitrogen, and mineral flame retardants [164].

**Figure 16:**
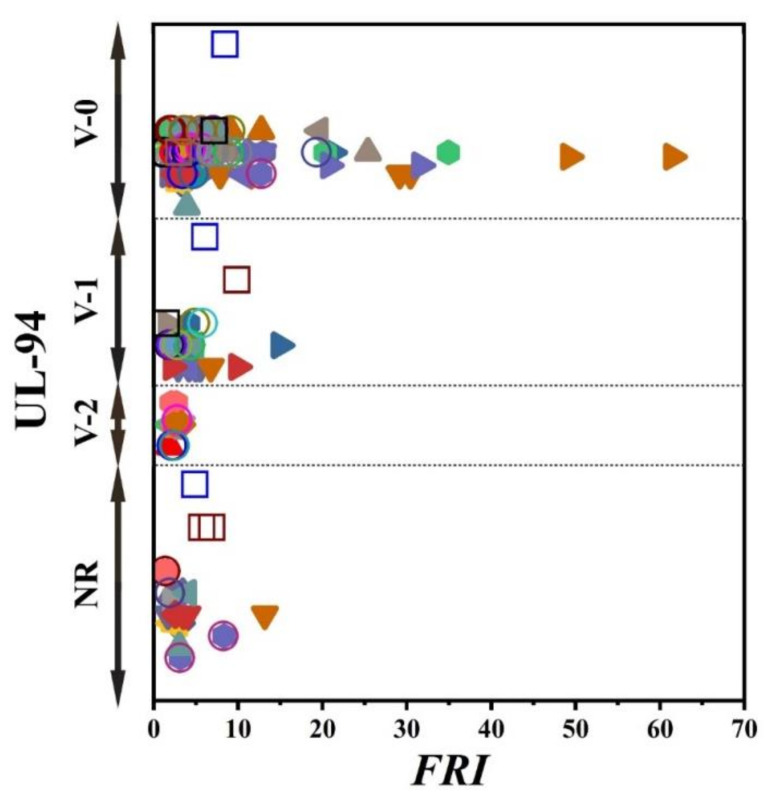
*FRI* values versus UL-94 test results. Symbols are indicative of combination of flame retardant (FR) additives used in PP. The vertical intervals in each category, i.e., V-0, V-1, V-2, and NR, are schematically representative of the amount of additive used. For example, two data distinguished by different symbols having the same or very close *FRI* values (horizontal quantity) in a given category (e.g., V-1), may have different vertical quantities, e.g., both reveal V-1 behavior in UL-94 test, but the upper contains more FR in PP.

**Figure 17:**
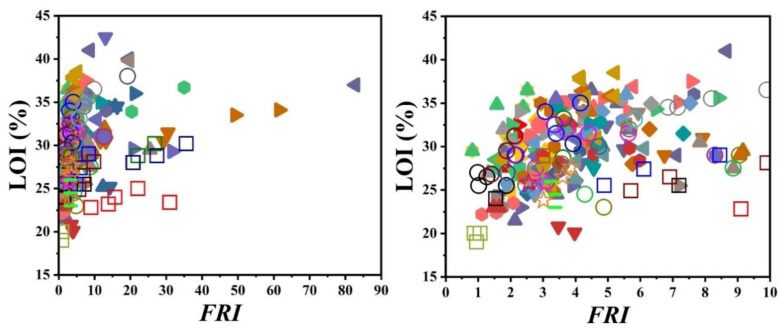
*FRI* values of PP as a function of LOI test results in long-shot (left-hand figure) and close-up (right-hand figure). Symbols are indicative of different types of blend flame retardants used. The left-side plot reveals that *FRI* values above 10 (*Excellent* zone) took place in several cases, which is in contradiction with all previous cases in which only one additive was used.

Figure 16 shows that V-0 level in UL-94 is automatically obtained in the case of combined flame retardant systems used in PP regardless of the *FRI* value. However, no correlation exists between the *FRI* and LOI (Figure 17). The complexity of polymer–filler interaction can be considered as the main reason for diversity of properties.

## 8. Conclusions and Future Perspective

This work opens new avenues to the experts working on “flame retardant polyolefins”, the title of a Special Issue entitled “Flame Retardant Polyolefins” in Polymers journal for which this work is designed and carried out. In this work, more than 150 research papers from the literature dealing with the flame retardancy of PP were analyzed, classified, and discussed in terms of flame retardancy performance. From the selected papers were extracted cone calorimetry data to calculate Flame Retardancy Index (*FRI*) as a measure or label of flame retardant performance. To have a comprehensive overview of flame retardant PP materials, works on PP flame retardancy were categorized in terms of additives used in classes including: phosphorus-based, nitrogen-based, mineral, carbon-based, bio-based, and hybrid combinatorial flame retardants composed of two or more additives. The analysis of efficiency of flame retardancy was performed in terms of the *FRI* variation as a function of wt.% of additives used. The analysis quite obviously unveiled the superiority of the combination of additives over the use of each one separately. In addition, the UL-94 and LOI values available in each class of additives were plotted in terms of the *FRI* so as to find possible correlation between analyses made in the literature. This work provided a pool of data on flame-retardant PP materials for future research on PP materials. It was elucidated that *FRI* can satisfactorily make possible classification of PP materials in terms of flame retardancy performance. The present work provides those research works that claim achieving synergistic effect of two or more flame retardants with a clear measure of flame retardant performance as *Poor*, *Good*, and *Excellent* labels assigned to PP materials, based on cone calorimetry data. Moreover, future works on LOI and UL-94 tests can be added to the data used here so as to draw a more detailed picture of flame retardancy behavior of PP materials. The approach can be used to make judgement about other flame retardant polyolefins. Moreover, we believe that the mechanical properties of FR polymers should also be considered in the future, but it is pertinent to the completeness of data in the literature. The importance of mechanical properties springs from the fact that highly loaded systems are prone to mechanical failure as a consequence of stress concentration. All in all, the type and the percentage of FRs in polymers affect both the mechanical and flame retardant properties of polymers; therefore, optimization of both properties is of importance.

## Figures and Tables

**Table 1:** Flame-retardant PP materials containing phosphorus-based (P) flame retardants. Data are extracted from the literature: cone calorimetry parameters (TTI, pHRR, THR), LOI, and UL-94 values. The *FRI* values were calculated by authors of the present review. The name and the percentage of flame retardants are provided in separate columns. “wt.%” was used for loading level of additives, while “―” stands for the systems free of additive or the neat PP. * FR means flame retardant. Since all comparisons were made in terms of *FRI*, classification of polymers in terms of their flame retardant properties was not surveyed based on the chemistry of additives, heat flux, sample thickness, etc.

PP Containing Phosphorus-Based (P) FR *	wt.%	TTI (s)	pHRR (kW·m^−2^)	THR (MJ·m^−2^)	Irradiance (kW·m^−2^)	Sample Thickness (mm)	*FRI*	LOI	UL-94	Ref.
	―	14	1104	106	35	0.4	―	―	―	[24]
**Ammonium polyphosphate (APP)**	10	24	925	92	35	0.4	2.35	―	―	[24]
	―	54	1610	106	35	3	―	20.8	NR	[25]
**APP**	12	37	510	97	35	3	2.36	22.3	V-2	[25]
**APP**	**15**	**27**	**339**	**89**	**35**	**3**	**2.82**	**25.4**	**V-0**	**[25]**
	―	34	1294	154.2	50	4	―	19	NR	[26]
**APP**	20	21	306	141.6	50	4	2.84	27	NR	[26]
	―	48	1351	107	35	3.2	―	18.5	NR	[27]
**APP**	20	40	787	92	35	3.2	1.66	20.5	NR	[27]
	―	24.3	1388.3	80.3	50	2.4	―	―	NR	[28]
**APP**	**20**	**19.3**	**254.8**	**54.5**	**50**	**2.4**	**6.37**	―	**V-0**	**[28]**
	―	66	633	44.2	35	3	―	17	NR	[23]
**APP**	20	31	424	38.6	35	3	0.80	21	NR	[23]
	―	18	1457	156	50	3	―	19	NR	[29]
**APP**	25	20	1455	148	50	3	1.17	21.9	V-2	[29]
	―	25	981	147	50	―	―	17.6	NR	[30]
**APP**	25	18	579	109	50	―	1.64	23.2	NR	[30]
	―	48	988	88.3	35	3.2	―	17	NR	[31]
**APP**	25	43	652	80	35	3.2	1.49	21	NR	[31]
	―	20	809	96	50	3	―	17.6	NR	[32]
**APP**	25	11	397	87	50	3	1.23	20.6	NR	[32]
	―	21	1242	111	50	3.2	―	18.6	NR	[33]
**APP**	25	21	979	107	50	3.2	1.31	21.7	NR	[33]
	―	35	1203	197.6	50	6	―	18.2	NR	[34]
**APP**	25	33	390.8	196	50	6	2.92	20.9	NR	[34]
	―	25	841.6	89.1	50	3	―	18	NR	[35]
**APP**	25	13	473.3	90.2	50	3	0.91	20	NR	[35]
**Piperazine-modified APP (m-APP)**	**25**	**17**	**162.6**	**84.5**	**50**	**3**	**3.71**	**32.5**	**V-0**	**[35]**
	―	33	1416	219	50	6	―	17	NR	[36]
**APP**	25	19	526	180	50	6	1.88	19.6	NR	[36]
**Polysiloxane shell-coated APP (mc-APP)**	25	19	214	137	50	6	6.08	25	NR	[36]
	―	45	759.2	98.8	35	3	―	17	NR	[37]
**Melamine and phytic acid-modified APP (m-APP)**	25	33	218.1	80.6	35	3	3.12	22.5	V-2	[37]
	―	37	1284	121	50	3	―	―	―	[38]
**APP**	30	22	767	111	50	3	1.08	21.7	NR	[38]
	―	48	988	88.3	35	3	―	17	NR	[39]
**APP**	30	32	459	77.6	35	3	1.63	22	NR	[39]
	―	50	1350	91.2	35	3	―	17	NR	[40]
**APP**	30	58	851	74.4	35	3	2.25	22	NR	[40]
	―	33	1238	123.7	50	3	―	17.8	NR	[41]
**Melamine-formaldehyde-tris(2-hydroxyethyl) isocyanurate resin microencapsulated APP (mc-APP)**	**30**	**24**	**375**	**116.4**	**50**	**3**	**2.55**	**32**	**V-0**	**[41]**
	―	44	831	158	35	3	―	17.5	NR	[42]
**APP**	30	30	432	114	35	3	1.81	22	NR	[42]
**Dipentaerythritol and 4,4′ diphenylmethanediisocyanate and melamine microencapsulated APP (mc-APP)**	**30**	**27**	**300**	**100**	**35**	**3**	**2.68**	**32.1**	**V-0**	[42]
	―	29	1186	215	50	6	―	17	NR	[43]
**APP**	30	18	543	180	50	6	1.61	20.1	NR	[43]
**Epoxy acrylate microencapsulated APP (mc-APP)**	30	13	332	149	50	6	2.31	24.8	NR	[43]
	―	40	1174.7	102.2	35	3	―	17	NR	[44]
**APP**	30	38	526.5	80.6	35	3	2.68	20	NR	[44]
**4,4′-diphenylmethane diisocyanate and melamine and pentaerythritol microencapsulated APP (mc-APP)**	30	30	301.8	65.1	35	3	4.58	25	V-1	[44]
	―	68	577.5	82.7	35	3	―	18.2	NR	[45]
**APP**	30	41	201.1	44.5	35	3	3.21	20.1	NR	[45]
**Thermoplastic polyurethane microencapsulated APP (mc-APP)**	5	57	395.4	67.2	35	3	1.50	18.7	NR	[45]
**Thermoplastic polyurethane microencapsulated APP (mc-APP)**	10	42	282.5	63.7	35	3	1.63	19.6	NR	[45]
**Thermoplastic polyurethane microencapsulated APP (mc-APP)**	15	40	214.9	59.9	35	3	2.18	20	NR	[45]
**Thermoplastic polyurethane microencapsulated APP (mc-APP)**	20	32	193.6	57.3	35	3	2.02	20.3	NR	[45]
**Thermoplastic polyurethane microencapsulated APP (mc-APP)**	25	30	145.4	64.1	35	3	2.26	22.2	NR	[45]
**Thermoplastic polyurethane microencapsulated APP (mc-APP)**	30	31	140.6	41.8	35	3	3.70	22.9	NR	[45]
	―	25	841.6	89.1	50	3	―	18	NR	[46]
**APP**	35	11	435.9	83.9	50	3	0.90	20.4	NR	[46]
**Ethylenediamine-modified APP (m-APP)**	**35**	**11**	**156.1**	**60.5**	50	3	**3.49**	**30.5**	**V-0**	[46]
	―	25	841.6	89.1	50	3	―	18	NR	[47]
**APP**	35	11	435.9	83.9	50	3	0.90	20.4	NR	[47]
**Ethanolamine-modified APP (m-APP)**	**35**	**18**	**96.6**	**22.6**	50	3	**24.73**	**35**	**V-0**	[47]
	―	33	837	212	50	6	―	17	NR	[48]
**APP**	40	30	440	186	50	6	1.97	20.8	NR	[48]
**Pentaerythritol triacrylate microencapsulated APP (mc-APP)**	**40**	**32**	**214**	**183**	50	6	**4.39**	**30.5**	**V-0**	[48]
	―	38	1284	214	50	6	―	18.2	NR	[49]
**APP**	25	34	537	177	50	6	2.58	20.9	NR	[49]
**Phosphorus-based charring agent: 3,9-Bis-(1-oxo-2,6,7-trioxa-1-phospha-bicyclo[2,2,2]oct-4-ylmethoxy)-2,4,8,10-tetraoxa-3,9 diphospha-spiro[5.5]undecane 3,9-dioxide (P-CA)**	25	35	480	168	50	6	3.13	22.6	NR	[49]
	―	42	831	112	35	3	―	18	NR	[50]
**APP**	25	36.4	578	83	35	3	1.68	21	NR	[50]
	―	36	1373	174.8	50	3	―	18.5	NR	[51]
**APP-based intumescent flame retardant (APP-IFR)**	**20**	**22**	**326**	**149.9**	50	3	**3.00**	**29.5**	**V-0**	[51]
	―	28	865	30.7	35	5	―	18.4	NR	[52]
**Phosphorus-based IFR: Six(1-oxo-2,6,7-trioxa-1-phosphabicyclo[2,2,2]octane-4-methyl) cyclotriphosphazene (P-IFR)**	10	28	595	28.2	35	5	1.58	19.7	NR	[52]
**Phosphorus-based IFR: Six(1-oxo-2,6,7-trioxa-1-phosphabicyclo[2,2,2]octane-4-methyl) cyclotriphosphazene (P-IFR)**	15	30	515	25.8	35	5	2.14	22.8	NR	[52]
**Phosphorus-based IFR: Six(1-oxo-2,6,7-trioxa-1-phosphabicyclo[2,2,2]octane-4-methyl) cyclotriphosphazene (P-IFR)**	20	33	433	23	35	5	3.14	26.1	V-2	[52]
**Phosphorus-based IFR: Six(1-oxo-2,6,7-trioxa-1-phosphabicyclo[2,2,2]octane-4-methyl) cyclotriphosphazene (P-IFR)**	**25**	**35**	**407**	**19.5**	35	5	**4.18**	**29.4**	**V-0**	[52]
	―	30	390	44	35	1.6	―	17.4	―	[53]
**Phosphorus-based IFR: Poly (4,4-diamino diphenyl methane Obicyclicpentaerythritol phosphate-phosphate) (P-IFR)**	20	24	224	27	35	1.6	2.26	25	―	[53]
	―	37	363	56	35	3	―	―	―	[54]
**Phosphorus-based IFR: compound containing Phosphorus(22%) and Nitrogene(18%) (P-IFR)**	28	33	62	24	35	3	12.18	―	―	[54]
	―	37	363	56	35	3	―	―	―	[55]
**Phosphorus-based IFR: compound containing Phosphorus(22%) and Nitrogene(18%) (P-IFR)**	28	33	62	24	35	3	12.18	―	―	[55]
	―	29	980	136	50	―	―	18.5	―	[56]
**Phosphorus and Nitrogene-based IFR**	30	22	229	93	50	―	4.74	36.3	―	[56]
	―	65	1416.6	128.5	35	3	―	―	NR	[57]
**Phosphorus-based flame retardant: Tri (1-oxo-2,6,7-trioxa-1-phosphabicyclo [2,2,2] octane-methyl) phosphate (P-FR)**	30	38	640.2	104.8	35	3	1.58	―	NR	[57]
	―	54	1199	97.8	35	―	―	―	―	[58]
**Phosphorus-based FR: Poly(4,4-diaminodiphenyl methane spirocyclicpentaerythritol bisphosphonate) (P-FR)**	20	69	620	78.5	35	―	3.07	―	―	[58]
	―	61	1026	166	35	4	―	―	―	[15]
**9,10-dihydro-9-oxa-10-phosphaphenanthrene-10-oxide (DOPO)**	10	60	648	141	35	4	1.83	―	―	[15]
	―	84	1000	96	35	3	―	―	―	[59]
**Tetraethyl orthosilicate microencapsulated bisphenol-A bis (diphenyl phosphate) (mc-BDP)**	10	57	808	101	35	3	0.79	―	―	[59]
**Tetraethyl orthosilicate microencapsulated BDP (mc-BDP)**	20	60	932	93	35	3	0.79	―	―	[59]
	―	44	1172	87.1	35	2.5	―	18.1	NR	[60]
**Organic phosphinate (OP)**	20	46	1052	84.2	35	2.5	1.20	20.1	V-2	[60]
	―	34	1052.4	90.8	50	3	―	17.5	NR	[61]
**Aluminum hypophosphite (AHP)**	24	23	267.1	77.3	50	3	3.13	―	―	[61]
	―	66	480	93	35	3	―	17	―	[62]
**Aluminium phosphinate (ALPi)**	30	73	524	89.8	35	3	1.04	26	―	[62]
	―	44	1175	106	35	3	―	―	―	[63]
**Pentaerythritol phosphate (PEPA)**	40	35	776	81	35	3	1.57	―	―	[63]

**Table 2:** Flame retardant PP materials containing nitrogen-based (N) flame retardants. Data are extracted from the literature: cone calorimetry parameters (TTI, pHRR, THR), LOI, and UL-94 values. The *FRI* values were calculated by authors of the present review. The name and the percentage of flame retardants are provided in separate columns. “wt.%” was used for loading level of additives, while “―” stands for the systems free of additive or the neat PP. * FR means flame retardant. Since all comparisons were made in terms of *FRI*, classification of polymers in terms of their flame-retardant properties was not surveyed based on the chemistry of additives, heat flux, sample thickness, etc.

PP Containing Nitrogen-Based (N) FR *	wt.%	TTI (s)	pHRR (kW·m^−2^)	THR (MJ·m^−2^)	Irradiance (kW·m^−2^)	Sample Thickness (mm)	*FRI*	LOI	UL-94	Ref.
	―	44	1175	106	35	3	―	―	―	[63]
**Melamine phosphate (MP)**	40	39	296	78	35	3	4.78	―	―	[63]
	―	54	930	140	35	4	―	―	NR	[64]
**Melamine salt of pentaerythritol phosphate kaolinite (MPPK)**	15	30	208	70	35	4	4.96	―	NR	[64]
**MPPK**	**20**	**28**	**148**	**42**	**35**	**4**	**10.86**	**―**	**V-0**	[64]
**MPPK**	**25**	**34**	**130**	**33**	**35**	**4**	**19.10**	**―**	**V-0**	[64]
	―	30	929	134	50	3	―	17	NR	[65]
**Melamine salt of tripentaerythriol phosphate (MTP)**	15	22	480	101	50	3	1.88	―	―	[65]
**MTP**	20	22	267	91	50	3	3.75	28	V-1	[65]
**MTP**	**25**	**22**	**226**	**73**	**50**	**3**	**5.53**	**32**	**V-0**	[65]
**MTP**	**30**	**22**	**219**	**72**	**50**	**3**	**5.78**	**35**	**V-0**	[65]
**Methyl hydrogen siloxane modified MTP (m-MTP)**	**30**	**21**	**253**	**72**	**50**	3	**4.78**	**30**	**V-0**	[65]
	―	65	1417	128.5	35	3	―	―	NR	[57]
**Melamine pyrophosphate (MPyP)**	30	36	437	103.1	35	3	2.23	―	NR	[57]
	―	34	1727	112	35	3	―	17	NR	[66]
**MPyP**	30	19	511	83	35	3	2.54	25.5	NR	[66]
**Triazine-based charring foaming agent: synthesized by reaction of cyanuric chloride and ethanolamine and ethylenediamine (TA-CFA)**	30	13	584	96	35	3	1.31	24	NR	[66]
	―	48	988	88.3	35	3	―	17	NR	[39]
**Triazine-based CFA: synthesized by reaction of cyanuric chloride and piperazine (TA-CFA)**	30	34	468	86.6	35	3	1.52	20.5	V-1	[39]
	―	50	1350	91.2	35	3	―	17	NR	[40]
**Triazine-based CFA: synthesized by polycondensation of 2-chloro-4,6-di-(2-hydroxyethylamino)-s-triazine (TA-CFA)**	30	38	518	86.7	35	3	2.08	23.5	NR	[40]
	―	45	759.2	98.8	35	3	―	17	NR	[37]
**Triazine-based CFA: synthesized from a macromolecular triazine derivative containing hydroxyethylamino and triazine rings and ethylenediamino groups (TA-CFA)**	25	34	487.4	91.6	35	3	1.26	21.9	NR	[37]
	―	20	809	96	50	3	―	17.6	NR	[32]
**Triazine-based CFA: Poly[N4-bis(ethylenediamino)-phenyl phosphonic-N2, N6-bis(ethylenediamino)-1,3,5-triazine-N-phenyl (TA-CFA)**	25	12	529	88	50	3	1.00	20.6	NR	[32]
	―	18	1457	156	50	3	―	19	NR	[29]
**Triazin-based CA—Zinc oxide (TA-CA-ZnO)**	25	17	694	149	50	3	2.07	18.3	NR	[29]
	―	41	840.3	115.7	35	3	―	16.4	NR	[67]
**Triazin-based CA: Poly(ethanediamine-1,3,5-triazine-p-4-amino-2,2,6,6-tetramethylpiperidine) (TA-CA)**	25	30	684	106.7	35	3	0.97	17.8	NR	[67]
	―	48	1351	107	35	3.2	―	18.5	NR	[27]
**Triazin-based CA: compound containing pentaerythritol and triazine structure (TA-CA)**	20	42	994	98	35	3.2	1.29	22	NR	[27]
	―	66	633	44.2	35	3	―	17	NR	[23]
**Triazin-based CA: synthesized by** **reaction of tris (2-hydrooxyethyl) isocyanurate and 2-carboxyethyl (phenyl) phosphinic acid (TA-CA)**	20	31	417	37.6	35	3	0.83	22	NR	[23]
	―	31	1239	123.6	50	3	―	18.5	NR	[68]
**Triazin-based IFR: synthesized by** **reaction of tris(2-hydroxyethyl) isocyanurate and polyphosphoric acid and melamine (TA-IFR)**	**20**	**18**	**289.9**	**89**	**50**	**3**	**3.44**	**29.3**	**V-0**	[68]
	―	48	988	88.3	35	3.2	―	17	NR	[31]
**Triazin-based IFR: synthesized by** **reaction of cyanuric chloride** **and N-amino ethylpiperazine (TA-IFR)**	25	38	504	86.6	35	3.2	1.58	23	V-1	[31]
	―	20	904.4	126.2	50	3	―	18	NR	[69]
**Piperazine-based FR: synthesized by** **reaction of diphenylphosphinyl chloride and piperazine (PI-FR)**	**25**	**58**	**487.7**	**87.5**	**50**	**3**	**7.75**	**27**	**V-0**	[69]
	―	45	1269	146.4	50	3	―	17.5	NR	[70]
**Piperazine-based IFR: Piperazine spirocyclic phosphoramidate (PI-IFR)**	**30**	**17**	**240.4**	**120.2**	**50**	**3**	**2.42**	**30.5**	**V-0**	[70]
	―	47	802	104	35	3	―	18	NR	[71]
**Piperazine-based IFR: synthesized by** **reaction of phosphorus chloride and 2,6,7-trioxa-l-phosphabicyclo[2,2,2]-octane-4-methanol and anhydrous piperazine (PI-IFR)**	20	36	275	78	35	3	2.97	24	NR	[71]
**Piperazine-based IFR: synthesized by** **reaction of phosphorus chloride and 2,6,7-trioxa-l-phosphabicyclo[2,2,2]-octane-4-methanol and anhydrous piperazine (PI-IFR)**	30	37	209	74	35	3	4.24	27	NR	[71]
**Piperazine-based IFR: synthesized by** **reaction of phosphorus chloride and 2,6,7-trioxa-l-phosphabicyclo[2,2,2]-octane-4-methanol and anhydrous piperazine (PI-IFR)**	**40**	**37**	**162**	**60**	**35**	**3**	**6.75**	**29**	**V-0**	[71]
	―	36	799.3	170.9	35	4	―	18	NR	[72]
**N-alkoxy hindered amine (NOR116)**	0.5	44	738.8	156.5	35	4	1.44	19	NR	[72]
	―	36	799	170	35	4	―	17.5	NR	[73]
**NOR116**	0.3	44	738	156	35	4	1.44	19.5	NR	[73]
	―	42	831	112	35	3	―	18	NR	[50]
**Polyurethane containing Phosphorus-based CA (PPU-CA)**	25	27.3	475	83	35	3	1.53	29	NR	[50]
	―	42	1025	137.7	35	4	―	―	―	[74]
**Nitrogen-based FR: compound containing Nitrogen (27.5.wt.%) and Phosphorus (15.6 wt.%) (N-FR)**	22	22	170	50.3	35	4	8.64	32	V-1	[74]
**Nitrogen-based FR: compound containing Nitrogen (27.5 wt.%) and Phosphorus (15.6 wt.%) (N-FR)**	**25**	**21**	**160**	**49.1**	**35**	**4**	**8.98**	**34**	**V-0**	[74]
	―	30	1093	108.2	50	3	―	18	NR	[75]
**Nitrogen-based IFR: Poly (diallyldimethylammonium) and polyphosphate polyelectrolyte complexe-based IFR (N-IFR)**	5	28	968.5	103.4	50	3	1.10	20.2	NR	[75]
**Nitrogen-based IFR: Poly (diallyldimethylammonium) and polyphosphate polyelectrolyte complexe-based IFR (N-IFR)**	10	25	626.2	97.1	50	3	1.62	22	NR	[75]
**Nitrogen-based IFR: Poly (diallyldimethylammonium) and polyphosphate polyelectrolyte complexe-based IFR (N-IFR)**	15	23	543.1	94.3	50	3	1.76	24.4	NR	[75]
**Nitrogen-based IFR: Poly (diallyldimethylammonium) and polyphosphate polyelectrolyte complexe-based IFR (N-IFR)**	20	21	443.9	90.1	50	3	2.06	26.3	NR	[75]
**Nitrogen-based IFR: Poly (diallyldimethylammonium) and polyphosphate polyelectrolyte complexe-based IFR (N-IFR)**	25	18	335.3	83.9	50	3	2.52	27.5	V-2	[75]
	―	25	874.1	89.3	50	3	―	18	NR	[76]
**Nitrogen-based IFR: compound containing Nitrogen (23%) and Phosphorus (21%) (N-IFR)**	**25**	**12**	**94.9**	**68.2**	**50**	**3**	**5.78**	**33**	**V-0**	[76]
	―	29	980	136	50	―	―	18.5	―	[56]
**Phosphorus and Nitrogene based IFR**	30	22	229	93	50	―	4.74	36.3	―	[56]

**Table 3:** Flame-retardant PP materials containing mineral-based (M) flame retardants. Data are extracted from the literature: cone calorimetry parameters (TTI, pHRR, THR), LOI, and UL-94 values. The *FRI* values were calculated by authors of the present review. The name and the percentage of flame retardants are provided in separate columns. “wt.%” was used for loading level of additives, while “―” stands for the systems free of additive or the neat PP. * FR means flame retardant. Since all comparisons were made in terms of *FRI*, classification of polymers in terms of their flame-retardant properties was not surveyed based on the chemistry of additives, heat flux, sample thickness, etc.

PP Containing Mineral-Based (M) FR *	wt.%	TTI (s)	pHRR (kW·m^−2^)	THR (MJ·m^−2^)	Irradiance (kW·m^−2^)	Sample Thickness (mm)	*FRI*	LOI	UL-94	Ref.
	―	37	1425	121.4	50	3	―	17.3	NR	[80]
**Aluminum trihydroxide (ATH)**	50	52	539	96.6	50	3	4.66	23.6	NR	[80]
	―	32	1470	175	50	4	―	18	―	[81]
**ATH**	60	34	280	98	50	4	9.96	25.6	―	[81]
	―	26	1967	112	50	3	―	―	―	[82]
**ATH**	20	27	817	90	50	3	3.11	―	―	[82]
**ATH**	40	28	467	70	50	3	7.25	―	―	[82]
**Magnesium dihydroxide (MDH)**	20	31	1000	98	50	3	2.68	―	―	[82]
**MDH**	40	34	433	75	50	3	8.87	―	―	[82]
	―	30	1684	89	50	3	―	―	―	[82]
**MDH**	40	3	377	71	50	3	0.55	―	―	[82]
**MDH**	60	29	228	51	50	3	12.46	―	―	[82]
	―	63.2	521.35	49.8	30	―	―	―	―	[83]
**MDH**	62.5	81.1	115.5	75.7	30	―	3.81	―	―	[83]
	―	71	2283	218	35	1	―	―	―	[84]
**MDH**	50	97	789	238	35	1	3.62	―	―	[84]
	―	38	1425	121.4	50	3	―	17.5	NR	[85]
**MDH**	40	46	548	99.1	50	3	3.85	23.3	NR	[85]
	―	29	1660	33.4	35	1	―	―	―	[86]
**MDH**	30	39	989	28.3	35	1	2.66	―	―	[86]
**Dodecanoic acid-treated MDH (m-MDH)**	30	32	882	28.7	35	1	2.41	―	―	[86]
**Dodecylphosphate treated MDH (m-MDH)**	30	29	651	28.8	35	1	2.95	―	―	[86]
	―	37	584	75.6	50	3	―	―	―	[87]
**MDH**	10	33	471	65.9	50	3	1.26	―	―	[87]
**MDH**	15	31	381	61.2	50	3	1.58	―	―	[87]
	―	54	930	140	35	4	―	―	NR	[64]
**Kaolinite (Kaol)**	25	32	463	116	35	4	1.43	―	NR	[64]
	―	29	1474	142	50	3	―	18	NR	[88]
**Kaol**	0.5	28	1429	142	50	3	0.99	18.3	NR	[88]
**Kaol**	1.5	27	1346	140	50	3	1.03	18.3	NR	[88]
**Kaol**	3	26	1279	135	50	3	1.08	18.4	NR	[88]
**Ammonium sulfamate intercalated kaol** **(m-Kaol)**	0.5	27	1389	141	50	3	0.99	18.4	NR	[88]
**Ammonium sulfamate intercalated kaol** **(m-Kaol)**	1.5	28	1169	133	50	3	1.29	18.6	NR	[88]
**Ammonium sulfamate intercalated kaol** **(m-Kaol)**	3	27	1079	126	50	3	1.43	18.7	NR	[88]
	―	27	1474	142	50	―	―	18	NR	[89]
**Kaol**	1.5	27	1346	140	50	―	1.11	18.3	NR	[89]
**Ammonium sulfamate intercalated Kaol** **(m-Kaol)**	1.5	28	1169	133	50	―	1.39	18.6	NR	[89]
	―	44	1000	145	50	―	―	―	―	[90]
**Kaol**	10	35	634	144	50	―	1.26	―	―	[90]
**Kaol**	20	38	396	136	50	―	2.33	―	―	[90]
**Kaol**	30	41	348	126	50	―	3.08	―	―	[90]
**Trisilanolisooctyl polyhedral oligomeric silsesquioxane modified kaol (m-Kaol)**	10	35	850	140	50	―	0.96	―	―	[90]
**Trisilanolisooctyl polyhedral oligomeric silsesquioxane modified kaol (m-Kaol)**	20	38	650	141	50	―	1.36	―	―	[90]
**Trisilanolisooctyl polyhedral oligomeric silsesquioxane modified kaol (m-Kaol)**	30	50	430	137	50	―	2.79	―	―	[90]
**Talc (TC)**	10	49	377	128	50	―	3.34	―	―	[90]
**TC**	20	56	341	118	50	―	4.58	―	―	[90]
**TC**	30	50	295	112	50	―	4.98	―	―	[90]
	―	45	1831.96	110.8	50	―	―	―	―	[91]
**Ni-Al layered double hydroxide (LDH)**	0.5	53	1635.53	106.8	50	―	1.36	―	―	[91]
**Ni-Al LDH (LDH)**	1	92	1430.59	117.8	50	―	2.46	―	―	[91]
**Ni-Al LDH (LDH)**	1.5	41	1266.66	129.1	50	―	1.13	―	―	[91]
**Organically modified Ni-Al LDH (m-LDH)**	0.5	59	1116.37	70.2	50	―	3.39	―	―	[91]
**Organically modified Ni-Al LDH (m-LDH)**	1	45	1026.86	81.24	50	―	2.43	―	―	[91]
**Organically modified Ni-Al LDH (m-LDH)**	1.5	49	1254.95	111.1	50	―	1.58	―	―	[91]
**Cu-Al LDH (LDH)**	0.5	45	1026.86	81.2	50	―	2.43	―	―	[91]
**Cu-Al LDH (LDH)**	1	57	1276.46	123	50	―	1.63	―	―	[91]
**Cu-Al LDH (LDH)**	1.5	50	1449.98	121.8	50	―	1.27	―	―	[91]
**Organically modified Cu-Al LDH (m-LDH)**	0.5	69	985.91	120	50	―	2.63	―	―	[91]
**Organically modified Cu-Al LDH (m-LDH)**	1	54	1175.99	121.6	50	―	1.70	―	―	[91]
**Organically modified Cu-Al LDH (m-LDH)**	1.5	54	1345.14	114.3	50	―	1.58	―	―	[91]
	―	20	1849	121	50	3	―	―	―	[92]
**Mg-Al LDH with mole ratio: Zn:Mg:Al/0:2:1 (A-LDH)**	1	15	1981	141	50	3	0.60	―	―	[92]
**A-LDH**	2	16	1764	139	50	3	0.73	―	―	[92]
**Zn-Mg-Al LDH with mole ratio: Zn:Mg:Al/0.5:1.5:1 (B-LDH)**	1	14	1997	136	50	3	0.57	―	―	[92]
**B-LDH**	2	14	1512	133	50	3	0.77	―	―	[92]
**B-LDH**	4	13	1153	128	50	3	0.98	―	―	[92]
**Zn-Mg-Al LDH with mole ratio: Zn:Mg:Al/1:1:1 (C-LDH)**	1	18	2004	135	50	3	0.74	―	―	[92]
**C-LDH**	2	14	1546	132	50	3	0.76	―	―	[92]
**C-LDH**	4	12	1225	125	50	3	0.87	―	―	[92]
**Zn-Mg-Al LDH with mole ratio: Zn:Mg:Al/1.5:0.5:1 (D-LDH)**	1	18	1938	135	50	3	0.76	―	―	[92]
**D-LDH**	2	15	1656	130	50	3	0.77	―	―	[92]
**D-LDH**	4	13	1294	123	50	3	0.91	―	―	[92]
**Zn-Al LDH with mole ratio: Zn:Mg:Al/2:0:1 (E-LDH)**	1	16	1977	136	50	3	0.66	―	―	[92]
**E-LDH**	2	17	1543	113	50	3	1.09	―	―	[92]
**E-LDH**	4	14	1382	126	50	3	0.89	―	―	[92]
	―	17	2380	140	50	3	―	―	―	[92]
**A-LDH**	1	20	1906	135	50	3	1.52	―	―	[92]
**A-LDH**	4	16	1137	129	50	3	2.13	―	―	[92]
**B-LDH**	1	17	1715	134	50	3	1.44	―	―	[92]
**B-LDH**	4	17	1025	124	50	3	2.62	―	―	[92]
**C-LDH**	1	16	1875	130	50	3	1.28	―	―	[92]
**C-LDH**	4	14	992	125	50	3	2.21	―	―	[92]
**D-LDH**	1	15	2008	135	50	3	1.08	―	―	[92]
**D-LDH**	4	16	997	126	50	3	2.49	―	―	[92]
**E-LDH**	1	17	1796	13	50	3	14.27	―	―	[92]
**E-LDH**	4	16	757	125	50	3	3.31	―	―	[92]
	―	26	1975	125	50	3	―	―	―	[92]
**A-LDH**	1	21	1831	149	50	3	0.73	―	―	[92]
**A-LDH**	4	23	1274	127	50	3	1.34	―	―	[92]
**B-LDH**	1	23	1838	135	50	3	0.88	―	―	[92]
**B-LDH**	4	18	1017	126	50	3	1.33	―	―	[92]
**C-LDH**	1	20	1676	137	50	3	0.82	―	―	[92]
**C-LDH**	4	17	981	124	50	3	1.32	―	―	[92]
**D-LDH**	1	19	1833	136	50	3	0.72	―	―	[92]
**D-LDH**	4	15	1061	126	50	3	1.06	―	―	[92]
**E-LDH**	1	18	1966	136	50	3	0.63	―	―	[92]
**E-LDH**	4	17	965	126	50	3	1.32	―	―	[92]
	―	23	1726	133	50	3	―	―	―	[92]
**A-LDH**	1	22	1763	121	50	3	1.02	―	―	[92]
**A-LDH**	4	20	1283	125	50	3	1.24	―	―	[92]
**C-LDH**	1	19	1795	131	50	3	0.80	―	―	[92]
**C-LDH**	4	16	897	121	50	3	1.47	―	―	[92]
**E-LDH**	1	21	1845	130	50	3	0.87	―	―	[92]
**E-LDH**	4	18	750	122	50	3	1.96	―	―	[92]
	―	16	1443	141	50	3	―	—	—	[93]
**Sodium dodecyl sulphate modified Ni-Al LDH (m-LDH)**	1	30	1100	122	50	3	2.84	―	―	[93]
**Sodium dodecyl sulphate modified Ni-Al LDH (m-LDH)**	3	32	1040	120	50	3	3.26	―	―	[93]
**Sodium dodecyl sulphate modified Ni-Al LDH (m-LDH)**	5	36	975	113	50	3	4.15	―	―	[93]
	―	44	1443	158	35	3	―	―	―	[94]
**Undecenoate modified Mg-Al LDH (m-LDH)**	3	40	1627	174	35	3	0.73	―	―	[94]
**Undecenoate modified Mg-Al LDH (m-LDH)**	5	32	1629	167	35	3	0.60	―	―	[94]
**Undecenoate modified Mg-Al LDH (m-LDH)**	10	42	1339	157	35	3	1.03	―	―	[94]
	―	31	1302	114	50	4	―	17.8	NR	[95]
**Carbonate intercalated Mg-Al LDH (LDH)**	10.7	22	837	78	50	4	1.61	19	NR	[95]
**Dihydrogen phosphate intercalated Mg-Al LDH (m-LDH)**	10.7	23	534	62	50	4	3.32	21.2	NR	[95]
	―	52	1792	219.4	35	―	―	―	―	[96]
**Octadecyltrimethyl ammonium chloride (alkyl-NH_4_Cl)**	1.2	53	1463	215.6	35	―	1.27	―	―	[96]
**Montmorillonite (MMT)**	5	45	1196	216.7	35	―	1.31	―	―	[96]
**Protoned MMT (H-MMT)**	5	42	1000	211.4	35	―	1.50	―	―	[96]
**Dioctadecyldimethyl ammonium chloride modified MMT (m-MMT)**	5	43	996	210.8	35	―	1.54	―	―	[96]
	―	55	1740	219.8	35	―	―	―	―	[96]
**Dioctadecyldimethyl ammonium chloride modified MMT (m-MMT)**	5	50	982	208.6	35	―	1.69	―	―	[96]
	―	37	2655	131	35	3	―	―	―	[97]
**Methyl tallow bis(2-hydroxyethyl) ammonium modified MMT (m-MMT)**	4.75	27	1365	123	35	3	1.51	―	―	[97]
**Silica pillared methyl tallow bis(2-hydroxyethyl) ammonium modified MMT (m-MMT)**	4.75	22	2585	132	35	3	0.60	―	―	[97]
**Silica pillared methyl tallow bis(2-hydroxyethyl) ammonium modified MMT powder supported with CuO (m-MMT)**	4.75	24	2315	132	35	3	0.73	―	―	[97]
	―	14	1104	106	35	0.4	―	―	―	[24]
**MMT**	10	18	1005	99	35	0.4	1.51	―	―	[24]
**Modified MMT (m-MMT)**	10	19	925	98	35	0.4	1.75	―	―	[24]
	―	53	1896	102	35	3	―	―	―	[98]
**Alkylstyrene surfactant modified MMT (m-MMT)**	3	50	1502	99	35	3	1.22	―	―	[98]
**Alkylstyrene surfactant modified MMT (m-MMT)**	10	50	1200	94	35	3	1.61	―	―	[98]
**Alkylstyrene surfactant modified MMT (m-MMT)**	16	51	882	95	35	3	2.22	―	―	[98]
	―	60	1136	296	35	―	―	―	―	[99]
**MMT**	2	51	633	295	35	―	1.53	―	―	[99]
**Alkylammonium modified MMT (m-MMT)**	2	58	870	297	35	―	1.25	―	―	[99]
**Alkylammonium modified MMT (m-MMT)**	5	55	459	295	35	―	2.27	―	―	[99]
**Alkylammonium modified MMT (m-MMT)**	10	56	357	293	35	―	3.00	―	―	[99]
	―	52	1897	101	35	3	―	―	―	[100]
**Ammonium salt of an oligomer modified MMT (m-MMT)**	3	48	1577	95	35	3	1.18	―	―	[100]
**Ammonium salt of an oligomer modified MMT (m-MMT)**	8	49	1309	97	35	3	1.42	―	―	[100]
**Ammonium salt of an oligomer modified MMT (m-MMT)**	12	52	1160	93	35	3	1.77	―	―	[100]
	―	43	1845	118	50 OR 35	—	―	—	—	[101]
**Styrene-vinylbenzyl chloride copolymer modified MMT (m-MMT)**	2.5	47	1953	114	50 OR 35	—	1.06	―	―	[101]
**Styrene-vinylbenzyl chloride copolymer modified MMT (m-MMT)**	5	45	1889	111	50 OR 35	—	1.08	―	―	[101]
**Styrene-vinylbenzyl chloride copolymer modified MMT (m-MMT)**	15	37	1448	108	50 OR 35	—	1.19	―	―	[101]
**Styrene-vinylbenzyl chloride copolymer modified MMT (m-MMT)**	25	38	1191	102	50 OR 35	—	1.58	―	―	[101]
**Methyl methacrylate-vinylbenzyl chloride copolymer modified MMT (m-MMT)**	2.5	44	2025	123	50 OR 35	—	0.89	―	―	[101]
**Methyl methacrylate-vinylbenzyl chloride copolymer modified MMT (m-MMT)**	5	42	1738	120	50 OR 35	—	1.01	―	―	[101]
**Methyl methacrylate-vinylbenzyl chloride copolymer modified MMT (m-MMT)**	15	39	1651	115	50 OR 35	—	1.04	―	―	[101]
**Methyl methacrylate-vinylbenzyl chloride copolymer modified MMT (m-MMT)**	25	41	1139	105	50 OR 35	—	1.73	―	―	[101]
	―	55	1586	113	35	—	―	—	—	[102]
**Methyl methacrylate modified MMT** **(m-MMT)**	1	66	1108	104	35	―	1.86	―	―	[102]
**Methyl methacrylate modified MMT** **(m-MMT)**	3	44	839	87	35	―	1.96	―	―	[102]
**Methyl methacrylate modified MMT** **(m-MMT)**	5	35	557	77	35	―	2.65	―	―	[102]
	―	50.2	789	156.6	35	―	―	17.5	―	[103]
**Nanofil (Nf)**	5	48	739	173.4	35	―	0.92	22	―	[103]
**Organically modified bentonite (m-BT)**	5	45.6	774	166.6	35	―	0.87	22	―	[103]
	―	33	847	159.8	50	―	―	17.5	―	[103]
**Nf**	5	37	1047	174	50	―	0.83	22	―	[103]
**m-BT**	5	36	1093	164	50	―	0.82	22	―	[103]
	―	35	1622	103	35	3	―	―	―	[104]
**Cloisite 20A: Dimethyl, dihydrogenated tallow ammonium modified MMT (C20A)**	1	33	1751	105	35	3	0.85	―	―	[104]
**C20A**	3	34	1874	107	35	3	0.80	―	―	[104]
**C20A**	5	39	1487	105	35	3	1.19	―	―	[104]
		44	1172	87.1	35	2.5	―	18.1	NR	[60]
**Cloisite 15A: dimethyl dehydrogenated tallow ammonium cation modified sodium MMT (C15A)**	5	41	1050	88.2	35	2.5	1.02	18.1	NR	[60]
	―	88	565.9	71.9	35	3	―	―	―	[105]
**C20A**	5	76	518.2	75.9	35	3	0.89	20	―	[105]
**C20A**	5	89	415.6	73	35	3	1.35	20	―	[105]
**Titanium dioxide (TiO_2_)**	0.5	99	488.1	75	35	3	1.25	20	―	[105]
	―	49	1247	114.2	35	―	―	―	―	[106]
**Activated alumina (Al_2_O_3_)**	2	35	943	108.2	35	―	0.99	―	―	[106]
	―	28	1633	132	50	4	―	18	―	[107]
**NiFeO**	2	27	1372	129	50	4	1.17	18	―	[107]
**CoFeO**	2	24	1335	127	50	4	1.08	18	―	[107]
	―	38	1284	241	50	6	―	―	―	[108]
**Ni_2_O_3_**	7.5	53	655	161	50	6	4.09	―	―	[108]
	―	64	1909	254	50	3	―	―	―	[109]
**Mo/Mg/Ni/O catalysts (Nmm-cat)**	1	62	490	205	50	3	4.67	―	―	[109]
**Nmm-cat**	2	63	292	168	50	3	9.72	―	―	[109]
**Nmm-cat**	3	60	275	149	50	3	11.09	―	―	[109]
	―	52	915.7	112.5	50	3	―	18	―	[110]
**Magnesium oxysulfate whisker (MOSw)**	30	62	259.1	90.4	50	3	5.24	24.7	―	[110]
**Dodecyl dihydrogen phosphate modified MOSw (m-MOSw)**	30	64	243.3	72.8	50	3	7.15	26.1	―	[110]
	―	48	195.5	28.6	35	2	―	―	―	[111]
**Manganese oxide (MnO)**	10	54	233.7	31.8	35	2	0.84	―	―	[111]
**Manganese oxide (Mn_2_O_3_)**	10	48	271.3	31.5	35	2	0.65	―	―	[111]
**Manganese oxalate (MnC_2_O_4_)**	10	50	281.6	29.3	35	2	0.70	―	―	[111]
	―	30	390	44	35	1.6	―	17.4	―	[53]
**Zinc acetyl acetonate (Znacac)**	1	31	366	28	35	1.6	1.73	19.5	―	[53]
**Chromium acetyl acetonate (Cracac)**	1	31	307	28	35	1.6	2.06	20.7	―	[53]
	―	51	1053	117.6	35	3	―	―	―	[112]
**Zirconium phenylphosphonate (ZrPP)**	2	34	754	99.4	35	3	1.10	―	―	[112]
	―	40	364	40	35	2	―	―	―	[113]
**Siloxane silsesquioxane resin (S4SQH)**	1	41	354	47	35	2	0.89	―	―	[113]
**S4SQH**	5	21	500	44	35	2	0.34	―	―	[113]
**S4SQH**	10	19	445	44	35	2	0.35	―	―	[113]
**n-octyl functionalized S4SQH (m-S4SQH)**	1	43	227	29	35	2	2.37	―	―	[113]
**n-octyl functionalized S4SQH (m-S4SQH)**	5	40	481	48	35	2	0.63	―	―	[113]
**n-octadecyl functionalized S4SQH (m-S4SQH)**	1	40	168	22	35	2	3.93	―	―	[113]
**n-octadecyl functionalized S4SQH (m-S4SQH)**	5	43	328	42	35	2	1.13	―	―	[113]
**n-octadecyl functionalized S4SQH (m-S4SQH)**	10	47	391	47	35	2	0.93	―	―	[113]
	―	25	981	147	50	―	―	17.6	NR	[30]
**Polysiloxane based FR (Si-FR)**	25	18	624	110	50	―	1.51	24.1	NR	[30]
	―	54	1610	106	35	3	―	20.8	NR	[25]
**Sepiolite (SEP)**	0.5	48	1701	108	35	3	0.82	20	NR	[25]
**Organically modified SEP (m-SEP)**	0.5	46	1665	106	35	3	0.82	19.2	NR	[25]
	―	37	584	75.6	50	3	―	―	―	[87]
**SEP**	5	24	533	68.1	50	3	0.78	―	―	[87]
**Organically treated SEP (m-SEP)**	5	23	515	66.1	50	3	0.80	―	―	[87]
	―	60	968	100	35	3	―	19.2	―	[114]
**Methyl polyhedral oligomeric silsesquioxane (me-POSS)**	1.95	54	1023	100	35	3	0.85	–	―	[114]
**me-POSS**	6.5	60	786	96	35	3	1.28	19.2	―	[114]
**Phenyl POSS (ph-POSS)**	3.75	61	858	98	35	3	1.17	–	―	[114]
**ph-POSS**	12.5	53	872	96	35	3	1.02	19.5	―	[114]
	―	56	1103	111	35	3	―	―	―	[115]
**Octaisobutyl POSS (T8-POSS)**	10	50	1325	112	35	3	0.73	―	―	[115]
**Al-POSS**	10	37	624	98	35	3	1.32	―	―	[115]
**Zn-POSS**	10	54	1069	108	35	3	1.02	―	―	[115]
	―	54	1242	221	35	6	―	17.5	―	[5]
**Silica aerogel (SA)**	10	57	892	203	35	6	1.60	25.1	―	[5]
	―	49.5	622	74.5	50	3	―	―	―	[116]
**Halloysite nanotube (HNT)**	8	44	495	68.5	50	3	1.21	―	―	[116]
**HNT-Water injection (HNT-W)**	8	45.5	451	66.5	50	3	1.42	―	―	[116]
	―	52.5	620	70.5	50	3	―	―	―	[116]
**HNT**	8	48	495	67	50	3	1.20	―	―	[116]
**HNT-W**	4	49	507	66.5	50	3	1.21	―	―	[116]
**HNT-W**	8	46	367	60.5	50	3	1.72	―	―	[116]
**HNT-W**	16	42.5	219	55	50	3	2.93	―	―	[116]
	―	35	749	90.1	50	3	―	―	―	[117]
**HNT**	5	34	936.7	98	50	3	0.71	―	―	[117]
**HNT**	10	31	773.6	99.3	50	3	0.77	―	―	[117]
**HNT**	15	32	557.9	91.9	50	3	1.20	―	―	[117]
**Melamine and phytic acid modified HNT** **(m-HNT)**	5	30	713.2	89.4	50	3	0.90	―	―	[117]
**Melamine and phytic acid modified HNT** **(m-HNT)**	10	28	708.4	93.4	50	3	0.81	―	―	[117]
**Melamine and phytic acid modified HNT** **(m-HNT)**	15	27	678.8	89.3	50	3	0.85	―	―	[117]

**Table 4:** Flame-retardant PP materials containing carbon-based (C) flame retardants. Data are extracted from the literature: cone calorimetry parameters (TTI, pHRR, THR), LOI, and UL-94 values. The *FRI* values were calculated by authors of the present review. The name and the percentage of flame retardants are provided in separate columns. “wt.%” was used for loading level of additives, while “―” stands for the systems free of additive or the neat PP. * FR means flame retardant. Since all comparisons were made in terms of *FRI*, classification of polymers in terms of their flame-retardant properties was not surveyed based on the chemistry of additives, heat flux, sample thickness, etc.

PP Containing Carbon-Based (C) FR *	wt.%	TTI (s)	pHRR (kW·m^−2^)	THR (MJ·m^−2^)	Irradiance (kW·m^−2^)	Sample Thickness (mm)	*FRI*	LOI	UL-94	Ref.
	―	49	1247	114.2	35	―	―	―	―	[106]
**Graphene (GN)**	2	35	989	107.9	35	―	0.95	―	―	[106]
**Activated alumina decorated GN (m-GN)**	2	41	866	110.5	35	―	1.24	―	―	[106]
	―	51	1053	118.4	35	3	―	―	―	[122]
**P-phenylenediamine modified reduced graphene oxide (m–rGNO)**	2	33	928	104	35	3	0.83	―	―	[122]
**Polyaniline nanofiber modified rGNO** **(m–rGNO)**	2	27	763	98.4	35	3	0.87	―	―	[122]
	―	45	1230	113.6	35	3	―	―	―	[123]
**rGNO**	2	30	1105	97.5	35	3	0.86	―	―	[123]
**Hexachlorocyclotriphosphazene modified rGNO (m–rGNO)**	2	27	967	112.9	35	3	0.76	―	―	[123]
**Hexachlorocyclotriphosphazene modified rGNO decoration with Ni(OH)_2_ nanosheet** **(m–rGNO)**	2	35	829	92	35	3	1.42	―	―	[123]
	―	51	1053	117.6	35	3	―	―	―	[112]
**rGNO**	2	31	835	98.3	35	3	0.91	―	―	[112]
**Zirconium phenylphosphonate decorated rGNO (m-rGNO)**	2	39	676	89.8	35	3	1.55	―	―	[112]
	―	50	1044	101.4	35	3	―	―	―	[124]
**Graphene oxide (GNO)**	2	33	979	108.2	35	3	0.65	―	―	[124]
**Melamine modified GNO (m-GNO)**	0.5	40	892	104.1	35	3	0.91	―	―	[124]
**Melamine modified GNO (m-GNO)**	1	37	834	100.6	35	3	0.93	―	―	[124]
**Melamine modified GNO (m-GNO)**	2	33	739	98.7	35	3	0.95	―	―	[124]
	―	38	1526	47.4	35	―	―	―	―	[125]
**GN**	2.5	39	1279	58.8	35	―	0.98	―	―	[125]
**GN-Nickel oxide (GN-NiO)**	2.5	35	1110	45.4	35	―	1.32	―	―	[125]
**GN and Ni–Ce mixed oxide (GN-NiCexOy)**	2.5	32	956	39.2	35	―	1.62	―	―	[125]
	―	32	909	45.8	35	―	―	―	―	[126]
**rGNO**	2	28	778	40	35	―	1.17	―	―	[126]
**Phosphomolybdic acid modified rGNO** **(m-rGNO)**	1	27	773	39.6	35	―	1.14	―	―	[126]
**Phosphomolybdic acid modified rGNO** **(m-rGNO)**	2	23	737	38.4	35	―	1.05	―	―	[126]
**Phosphomolybdic acid modified rGNO** **(m-rGNO)**	3	25	700	38.4	35	―	1.21	―	―	[126]
	―	54	1199	97.8	35	―	―	―	―	[58]
**Poly(4,4-diaminodiphenyl methane spirocyclicpentaerythritol bisphosphonate)-4,4-diaminodiphenyl methane modified rGNO (m-rGNO)**	20	66	397	73.9	35	―	4.88	―	―	[58]
	―	66	383	76	35	3	―	―	―	[127]
**Expandable graphite with commercial name ES 350 F5 (EG(ES 350 F5))**	10	32	91	56	35	3	2.76	―	―	[127]
**EG with commercial name ES 700 F5 (EG(ES 700 F5))**	10	35	92	57	35	3	2.94	―	―	[127]
**EG with commercial name Nyagraph FP (EG(Nyagraph FP))**	10	44	92	66	35	3	3.19	―	―	[127]
**EG with commercial name TEG 315 (EG(TEG 315))**	10	53	134	69	35	3	2.52	―	―	[127]
**EG with commercial name Nyagraph KP251 (EG(Nyagraph KP251))**	10	54	308	69	35	3	1.12	―	―	[127]
	―	38	1361	85	35	3	―	―	―	[128]
**Carbon nanotube (CNT)**	1	37	431	75	35	3	3.48	―	―	[128]
**Modofied CNT (m-CNT)**	0.5	42	361	62	35	3	5.71	―	―	[128]
**Modofied CNT (m-CNT)**	1	42	342	66	35	3	5.66	―	―	[128]
**Modofied CNT (m-CNT)**	2	41	386	70	35	3	4.61	―	―	[128]
**Modofied CNT (m-CNT)**	4	39	450	79	35	3	3.33	―	―	[128]
	―	40	1360	80	35	3	―	―	―	[129]
**CNT**	1	35	462	73	35	3	2.82	―	―	[129]
**Fullerene C60 decorated CNT (m-CNT)**	0.5	38	443	71	35	3	3.28	―	―	[129]
**Fullerene C60 decorated CNT (m-CNT)**	1	39	400	69	35	3	3.84	―	―	[129]
**Fullerene C60 decorated CNT (m-CNT)**	2	38	385	65	35	3	4.13	―	―	[129]
	―	35	1203	208	50	6	―	18.2	―	[130]
**Multiwall carbon nanotube (MWCNT)**	1	24	945	211	50	6	0.86	19.3	―	[130]
**MWCNT**	3	23	845	208	50	6	0.93	21.8	―	[130]
**MWCNT**	5	23	553	199	50	6	1.49	23.4	―	[130]
**Modified MWCNT (m-MWCNT)**	1	25	775	208	50	6	1.10	20	―	[130]
**Modified MWCNT (m-MWCNT)**	3	24	670	200	50	6	1.28	22.6	―	[130]
**Modified MWCNT (m-MWCNT)**	5	24	485	198	50	6	1.78	24.1	―	[130]
	―	30	1261	208	50	6	―	18	―	[131]
**MWCNT**	1	21	678	195	50	6	1.38	19.5	―	[131]
**MWCNT**	3	20	584	192	50	6	1.55	22.6	―	[131]
	―	24	1620	110	50	3	―	―	―	[132]
**MWCNT**	3	17	931	102	50	3	1.32	―	―	[132]
	―	38	1284	214	50	6	―	18.2	―	[133]
**MWCNT**	10	25	367	199	50	6	2.47	24.6	―	[133]
**Carbon fiber (CF)**	10	30	915	207	50	6	1.14	20.6	―	[133]
	―	35	1212	198	50	6	―	18.2	―	[134]
**CF**	3	25	1203	203	50	6	0.70	19.9	―	[134]
**CF**	8	26	777	198	50	6	1.15	20.2	―	[134]
**Carbon black (CB)**	5	23	417	186	50	6	2.03	24.6	―	[134]
	―	38	1284	241	50	6	―	―	―	[108]
**Activated carbon (AC)**	7.5	15	682	185	50	6	0.96	―	―	[108]
	―	48	1518	112.4	35	3.1	―	―	―	[6]
**Vapor grown carbon nanofiber (VGCNF)**	4	35	610	113.1	35	3.1	1.80	―	―	[6]
**VGCNF**	8	47	525	108.6	35	3.1	2.93	―	―	[6]
**VGCNF**	12	49	547	102.4	35	2.9	3.10	―	―	[6]

**Table 5:** Flame-retardant PP materials containing bio-based (Bio) flame retardants. Data are extracted from the literature: cone calorimetry parameters (TTI, pHRR, THR), LOI, and UL-94 values. The *FRI* values were calculated by authors of the present review. The name and the percentage of flame retardants are provided in separate columns. “wt.%” was used for loading level of additives, while “―” stands for the systems free of additive or the neat PP. * FR means flame retardant. Since all comparisons were made in terms of *FRI*, classification of polymers in terms of their flame-retardant properties was not surveyed based on the chemistry of additives, heat flux, sample thickness, etc.

PP Containing Bio-Based (Bio) FR *	wt.%	TTI (s)	pHRR (kW·m^−2^)	THR (MJ·m^−2^)	Irradiance (kW·m^−2^)	Sample Thickness (mm)	*FRI*	LOI	UL-94	Ref.
	―	46	1541	90	35	3	―	―	―	[135]
**Cyclodextrin nanosponge (CD)**	10	34	1462	80	35	3	0.87	―	―	[135]
	―	61	1026	166	35	4	―	―	―	[15]
**Hydroxyapatite and Cyclodextrin-based FR (HAandCD-FR)**	10	32	708	156	35	4	0.80	―	―	[15]
**Propylene-block-ethylene copolymer**	―	49	1350	87.3	35	3	―	17.5	―	[136]
**Phosphorus and nitrogen elements modified lignin (m-lig)**	20	38	380	74.2	35	3	3.24	22.5	―	[136]
	―	22.5	1004.7	122.6	50	3.2	―	18	NR	[137]
**Phytic acid and Piperazine-based FR (PHPI-FR)**	15	17.5	388.5	108.5	50	3.2	2.27	24	V-2	[137]
**PHPI-FR**	**18**	**17**	**386.2**	**108.4**	**50**	**3.2**	**2.22**	**25**	**V-0**	[137]
**PHPI-FR**	**20**	**17**	**346**	**106.1**	**50**	**3.2**	**2.53**	**25.5**	**V-0**	[137]
**PHPI-FR**	**25**	**16.5**	**303.4**	**105.4**	**50**	**3.2**	**2.82**	**27**	**V-0**	[137]
	―	29	1054	97	50	―	―	―	―	[138]
**Biochar (BC)**	15	12	753.01	112.68	50	―	0.49	―	―	[138]
**BC**	25	13.3	616.31	111.26	51	―	0.68	―	―	[138]
**BC**	30	15	539.34	101.2	52	―	0.96	―	―	[138]
**BC**	35	16.3	477.22	98.31	53	―	1.22	―	―	[138]
	―	24.3	1388.3	80.3	50	2.4	―	―	NR	[28]
**Wool**	40	12.3	858.7	77.3	50	2.4	0.85	―	NR	[28]
**Phosphoric acid-treated wool fiber (m-wool)**	40	14.3	426.7	72	50	2.4	2.13	―	NR	[28]
**Phosphoric acid-treated wool fiber (m-wool)**	**40**	**15**	**436.3**	**65.3**	**50**	**2.4**	**2.41**	―	**V-0**	[28]
**Phosphoric acid-treated chicken feather** **(m-CF)**	**40**	**14.7**	**336.7**	**57**	**50**	**2.4**	**3.51**	―	**V-0**	[28]
	―	24.7	1198.2	78.7	50	2.4	―	―	NR	[139]
**Chicken feather (CF)**	40	17	1234.1	76.1	50	2.4	0.69	―	NR	[139]
**Phosphoric acid and ethylenediamine treated chicken feather (m-CF)**	**40**	**19.3**	**280.5**	**58.7**	**50**	**2.4**	**4.47**	―	**V-0**	[139]
**Phosphoric acid and ethylenediamine treated chicken feather (m-CF)**	**40**	**17.7**	**216.1**	**52.4**	**50**	**2.4**	**5.96**	―	**V-0**	[139]

**Table 6:** The flame retardancy performance of PP containing various combinations of flame retardants in terms of *FRI* (* the name and percentage of incorporated flame retardants are given after PP). P = phosphorus FR, Np = non-phosphorus FR, N = nitrogen FR, nN = non-nitrogen-based FR, M = mineral FR, Bio = bio-based FR, nBio = non bio-based FR (one can also consider some nitrogen-based FRs containing phosphorus element as the combination of phosphorus and nitrogen resulting in synergism, Table 2). Since all comparisons were made in terms of *FRI*, classification of polymers in terms of their flame-retardant properties was not surveyed based on the chemistry of additives, heat flux, sample thickness, etc.

Name	wt.%	Type of FR	TTI (s)	pHRR (kW·m^−2^)	THR (MJ·m^−2^)	Irradiance (kW·m^−2^)	Sample Thickness (mm)	*FRI*	LOI	UL-94	Ref.
	―	―	31	1239	123.6	50	3	―	18.5	NR	[68]
**APP/Pentaerythritol (APP/PER)**	20	P:nP 2:1	18	514.7	92.6	50	3	1.86	27.6	V-2	[68]
	―	―	25	1239	123.6	50	3	―	18.5	NR	[143]
**APP/PER**	25	P:nP 2:1	17	442.3	98.8	50	3	2.38	30.1	V-0	[143]
	―	―	41	840.3	115.7	35	3	―	16.4	NR	[67]
**APP/PER**	25	P:nP 3:1	32	354.7	82	35	3	2.60	24.7	V-1	[67]
	―	―	34	1727	112	35	3	―	18	NR	[144]
**APP/PER**	30	P:nP 3:1	13	392	80	35	3	2.35	31	V-0	[144]
**Hydroxyl silicone oil co-microencapsulated APP and PER (mc-(APPandPER))**	30	P:nP 3:1	10	325	78	35	3	2.24	32.5	V-0	[144]
	―	―	166	412	105	25	3	―	―	―	[145]
**APP/PER /Melamine (APP/PER/MEL)**	29.4	P:nP:nP 1.07:1:0.92	175	76.4	55	25	3	10.85	―	―	[145]
**APP/PER/MEL**	33.2	P:nP:nP 1.64:1:0.94	188	65	46.3	25	3	16.27	―	―	[145]
**APP/PER/MEL**	36.2	P:nP:nP 2.14:1:0.92	180	68	49.4	25	3	13.96	―	―	[145]
	―	―	24	687	119	50	3	―	―	―	[145]
**APP/PER/MEL**	29.4	P:nP:nP 1.07:1:0.92	33	158	72.5	50	3	9.81	―	―	[145]
**APP/PER/MEL**	33.2	P:nP:nP 1.64:1:0.94	36	115	67.8	50	3	15.72	―	―	[145]
**APP/PER/MEL**	36.2	P:nP:nP 2.14:1:0.92	30	133	73.2	50	3	10.49	―	―	[145]
	―	―	24	687	119	25	3	―	17.8	―	[146]
**APP/PER/MEL**	33.2	P:nP:nP 1.64:1:0.93	36	115	67.8	25	3	15.72	34.5	―	[146]
**APP/PER/MEL/MDH**	37.3	P:nP:nP:nP 1.64:1:0.9:0.7	36	156	63.9	25	3	12.30	25.2	―	[146]
	―	―	166	412	105	50	3	―	17.8		[146]
**APP/PER/MEL**	33.2	P:nP:nP 1.64:1:0.93	188	65	46.3	50	3	16.27	34.5	―	[146]
**APP/PER/MEL/MDH**	37.3	P:nP:nP:nP 1.64:1:0.9:0.7	196	79.5	45.1	50	3	14.24	25.2	―	[146]
	―	―	35	1622	103	35	3	―	―	―	[104]
**APP/PER/MEL/C20A**	21	P:nP:nP:nP 3:1:1:0.25	25	463	89	35	3	2.89	―	―	[104]
**APP/PER/MEL/C20A**	23	P:nP:nP:nP 3:1:1:0.75	26	430	91	35	3	3.17	―	―	[104]
**APP and MMT/PER/MEL/C20A**	21	P:nP:nP:nP 3:1:1:0.25	24	306	81	35	3	4.62	―	―	[104]
**APP and MMT/PER/MEL/C20A**	23	P:nP:nP:nP 3:1:1:0.75	21	344	80	35	3	3.64	―	―	[104]
	―	―	48	662	83.3	35	4	―	17.6	NR	[147]
**γ-aminopropyltriethoxysilane modified APP/Dipentaerythritol/MEL** **(m-APP/DPER/MEL)**	25	P:nP:nP 4:1:1	30	71	32.5	35	4	14.93	34.4	V-1	[147]
**γ-aminopropyltriethoxysilane modified APP/ DPER/MEL/SEP** **(m-APP-/DPER/MEL/SEP)**	25	P:nP:nP:nP 4:1:1:0.25	29	51	30.8	35	4	21.20	36	V-0	[147]
	―	―	48	1007	126.3	35	3.2	―	17.5	NR	[148]
**APP/PER**	18	P:nP 3:1	45	423.5	112	35	3.2	2.51	24.6	NR	[148]
**APP/PER/Melamine fomaldehyde (APP/PER/MF)**	18	P:nP:nP 3:1:0.2	48	352.3	110.4	35	3.2	3.27	26.3	NR	[148]
**APP/PER/Adenosine monophosphate embedded Melamine fomaldehyde (APP/PER/MFA)**	18	P:nP:nP 3:1:0.2	49	355.1	108.1	35	3.2	3.38	27	V-0	[148]
	―	―	70	1267	147	50	3	―	17.5	NR	[149]
**APP/PER**	30	P:nP 3:1	40	321	80	50	3	4.14	29.5	V-1	[149]
	―	―	47	991.4	140.8	35	3.2	―	18.4	NR	[150]
**APP/PER**	18	P:nP 2.4:1	44	434.1	123.8	35	3.2	2.43	26.2	NR	[150]
**APP/PER /Triazine-based FR: N, N′, N″-1, 3, 5-triazine-2, 4, 6-triyltris-glycine (APP/PER/TA-FR)**	18	P:nP:nP 2.4:1:0.2	42	323.3	126	35	3.2	3.06	29.5	V-0	[150]
	―	―	36	799.3	170.9	35	4	―	18	NR	[72]
**APP/PER**	25	P:nP 2:1	23	322	144.5	35	4	1.87	31	V-1	[72]
**APP/PER/NOR116**	25	P:nP:nP 2:1:0.06	33	313.8	136.5	35	4	2.92	35	V-0	[72]
	―	―	51	888	125	35	3.2	―	17.5	NR	[151]
**APP/PER**	18	P:nP 3:1	45	439	111	35	3.2	2.00	24.6	NR	[151]
**APP/PER/Guanine: nitrogenous bases (APP/PER/G-bases)**	18	P:nP:nP 3:1:0.3	46	324	105	35	3.2	2.94	27.6	NR	[151]
**APP/PER/Uracil: nitrogenous bases (APP/PER/U-bases)**	18	P:nP:nP 3:1:0.3	46	293	105	35	3.2	3.25	28.7	V-0	[151]
	―	―	38	886	144	50	3	―	17.6	NR	[152]
**APP/DPER**	25	P:nP 2.2:1	30	386	117	50	3	2.23	26.8	V-1	[152]
**Aluminum chloride modified APP/ DPER (m-APP/DPER)**	25	P:nP 2.2:1	25	226	104	50	3	3.57	32.1	V-0	[152]
**APP/DPER/ATH**	25	P:nP:nP 2:1:0.1	28	381	108	50	3	2.28	28.7	V-0	[152]
	―	―	31	1002	114	50	4	―	17.8	NR	[70]
**APP/PER**	28.5	P:nP 3:1	24	318	122	50	4	2.27	30	V-0	[70]
**APP/PER/Kaol**	28.5	P:nP:nP 3:1:0.2	22	222	131	50	4	2.78	33	V-0	[70]
	―	―	70	415.6	99.6	35	3	―	17	NR	[153]
**APP/PER**	29	P:nP 3:1	21	160.7	94.2	35	3	0.82	29.5	V-0	[153]
**APP/PER/MMT**	29	P:nP:nP 3:1:0.46	42	149.8	69.5	35	3	2.38	34.5	V-0	[153]
**APP/PER/Melamine modified MMT (APP/PER/m-MMT)**	29	P:nP:nP 3:1:0.46	37	157.9	55.1	35	3	2.51	36.5	V-0	[153]
	―	―	70	415.6	99.6	35	3	―	17	NR	[154]
**APP/PER**	29	P:nP 3:1	21	160.7	94.2	35	3	0.82	29.5	V-0	[154]
**APP/PER/MMT**	29	P:nP:nP 3:1:0.46	42	149.8	69.5	35	3	2.38	34.5	V-0	[154]
**APP/PER/Melamine modified MMT (APP/PER/m-MMT)**	29	P:nP:nP 3:1:0.46	37	157.9	55.1	35	3	2.51	36.5	V-0	[154]
**APP/PER/Triphenylphonium modified MMT (APP/PER/m-MMT)**	29	P:nP:nP 3:1:0.46	38	168.2	84.7	35	3	1.57	34.8	V-0	[154]
	―	―	50	720	136	35	4	―	17	NR	[155]
**APP/PER**	25	P:nP 3:1	39	267	111	35	4	2.57	26.3	V-1	[155]
**APP/PER/Zn-Ni-Al LDH(APP/PER/LDH)**	25	P:nP:nP 3:1:0.16	35	296	109	35	4	2.12	27	V-1	[155]
**APP/PER/Azobenzene-4,4′-dicarboxylic acid modified Ni-Zn-Al LDH** **(APP/PER/m-LDH)**	25	P:nP:nP 3:1:0.16	39	271	102	35	4	2.76	29.3	V-0	[155]
	―	―	53	655	108.1	35	3	―	17.1	NR	[156]
**APP/PER**	25	P:nP 3:1	32	261	93.4	35	3	1.75	28.9	V-2	[156]
**APP/PER/Acid-treated waste silicon rubber composite insulator (APP/PER/m-SiR)**	25	P:nP:nP 3:1:0.16	29	273	92.6	35	3	1.53	28.5	V-0	[156]
**APP/PER/Acid and N_2_ plasma-treated SiR (APP/PER/m-SiR)**	25	P:nP:nP 3:1:0.16	29	205	91.8	35	3	2.05	30.9	V-0	[156]
**APP/PER/Acid and N_2_ plasma-treated SiR (APP/PER/m-SiR)**	25	P:nP:nP 3:1:0.16	24	208	97.5	35	3	1.58	27.4	V-0	[156]
	―	―	30	930	135	50	3	―	17	NR	[157]
**Methyl hydrogen siloxane-treated APP/DPER (m-APP/DPER)**	25	P:nP 2:1	19	347	113	50	3	2.02	32.5	V-0	[157]
**Methyl hydrogen siloxane-treated APP/DPER/Zeolite (m-APP/DPER/Z)**	26	P:nP:nP 2:1:0.1	21	209	50	50	3	8.41	35.6	V-0	[157]
**Methyl hydrogen siloxane-treated APP/DPER/ Z/MWCNT** **(m-APP/DPER/Z/MWCNT)**	26.1	P:nP:nP:nP 2:1:0.1:0.01	21	226	60	50	3	6.48	34.3	V-0	[157]
	―	―	76	590	93	35	3	―	18	NR	[158]
**APP/PER**	25	P:nP 3:1	55	200	74	35	3	2.68	27	V-1	[158]
**APP/PER/Allophane:hydrated aluminosilicate (APP/PER/ALL)**	27	P:nP:nP 3:1:0.3	53	149	68	35	3	3.77	35	V-0	[158]
	―	―	28	1547	123	50	3	―	17.7	NR	[159]
**APP/PER**	25	P:nP 2:1	18	436	123	50	3	2.28	27.6	V-1	[159]
**APP/PER/Mesoporous aluminosilicate oxide (APP/PER/MAO)**	25	P:nP:nP 2:1:0.33	31	188	55	50	3	20.37	33.9	V-0	[159]
**APP/PER/Zn-MAO**	25	P:nP:nP 2:1:0.33	28	136	40	50	3	34.97	36.7	V-0	[159]
	―	―	38	1060	151.1	50	4	―	18.5	NR	[160]
**APP/PER**	25	P:nP 2:1	24	263	134.6	50	4	2.85	28.8	V-1	[160]
**APP/PER/Organo modified SEP (APP/PER/m-SEP)**	25	P:nP:nP 2:1:0.125	27	223	105.6	50	4	4.83	30	V-0	[160]
**APP/PER/Organo modified SEP (APP/PER/m-SEP)**	25	P:nP:nP 2:1:0.25	30	247	114.1	50	4	4.48	36.5	V-0	[160]
**APP/PER/Organo modified SEP (APP/PER/m-SEP)**	25	P:nP:nP 2:1:0.41	31	237	104.3	50	4	5.28	35	V-0	[160]
**APP/PER/Organo modified SEP (APP/PER/m-SEP)**	25	P:nP:nP 2:1:0.57	29	263	135.8	50	4	3.42	24.5	NR	[160]
**APP/PER/Organo modified SEP (APP/PER/m-SEP)**	25	P:nP:nP 2:1:0.74	29	362	158.4			2.13	21.5	NR	[160]
	―	―	43	460	58	35	3	―	17.7	NR	[161]
**APP/PER**	20	P:nP 3:1	27.2	229	44	35	3	1.67	24.5	NR	[161]
**APP/PER/Octaphenyl POSS** **(APP/PER/OP-POSS)**	20	P:nP:nP 3:1:0.2	33.2	199	36	35	3	2.87	26	V-1	[161]
**APP/PER/Aminopropyl isobutyl-octaphenyl POSS (APP/PER/A-POSS)**	20	P:nP:nP 3:1:0.2	32.3	178	27	35	3	4.17	28.1	V-1	[161]
**APP/PER/Octaammonium POSS (APP/PER/OA-POSS)**	20	P:nP:nP 3:1:0.2	37.7	164	26	35	3	5.48	29.7	V-1	[161]
**APP/PER/Trissulfonic acid propyl POSS (APP/PER/TS-POSS)**	20	P:nP:nP 3:1:0.2	35.4	153	29	35	3	4.95	32.4	V-1	[161]
	―	―	59	347.1	80.97	35	3	―	17	―	[162]
**APP/PER**	30	P:nP 2:1	52.5	70.43	49.96	35	3	7.10	29	―	[162]
**APP/PER/Thermally-treated solid waste (APP/PER/T-RS)**	33.5	P:nP:nP 2:1:0.5	48	65.71	40.21	35	3	8.65	41	―	[162]
**APP/PER/Volcanic ash (APP/PER/CV)**	33.5	P:nP:nP 2:1:0.5	101	19.73	29.48	35	3	82.72	37	―	[162]
**APP/PER/Rice husk ash (APP/PER/CR)**	33.5	P:nP:nP 2:1:0.5	62	48.16	31.36	35	3	19.55	40		[162]
	―	―	65	920	145	35	4	―	17.5	NR	[163]
**APP/PER**	20	P:nP 3:1	32	305	93	35	4	2.31	23	NR	[163]
**APP/PER/Zinc borate (APP/PER/ZnB)**	20	P:nP:nP 3:1:0.2	37	330	125	35	4	1.84	29.5	V-0	[163]
**APP/PER/Borophosphate (APP/PER/BPO_4_)**	20	P:nP:nP 3:1:0.2	33	226	53	35	4	5.65	30	V-0	[163]
**APP/PER/Boron silicon containing preceramic oligomer (APP/PER/Bsi)**	20	P:nP:nP 3:1:0.2	34	255	70	35	4	3.90	25.5	V-0	[163]
**APP/PER/Lanthanum borate (APP/PER/LaB)**	20	P:nP:nP 3:1:0.2	43	260	97	35	4	3.49	27	V-0	[163]
	―	―	28	1633	132	50	4	―	18	―	[107]
**APP/PER**	25	P:nP 2:1	21	483	116	50	4	2.88	27.5	―	[107]
**APP/PER/NiFeO**	25	P:nP:nP 2:1:0.35	20	425	107	50	4	3.38	34.6	―	[107]
**APP/PER/CoFeO**	25	P:nP:nP 2:1:0.35	19	323	124	50	4	3.65	35	―	[107]
	―	―	28	1337	95.1	35	3	―	18	NR	[164]
**APP/PER**	25	P:nP 2:1	41	588.8	88.4	35	3	3.57	28	V-0	[164]
**APP/PER/Nickel phosphide nanocrystalline (APP/PER/Ni_12_P_5_)**	25	P:nP:P 2:1:0.26	54	363.2	88.2	35	3	7.65	36	V-0	[164]
**APP/PER/Cobaltous phosphide nanocrystalline (APP/PER/Co_2_P)**	25	P:nP:P 2:1:0.26	79	306.6	89.1	35	3	13.12	34	V-0	[164]
**APP/PER/Cupric phosphide nanocrystalline (APP/PER/Cu_3_P)**	25	P:nP:P 2:1:0.26	42	562.4	93.2	35	3	3.63	31.5	V-1	[164]
	―	―	75	471	102	50	3	―	18	NR	[165]
**APP/PER**	25	P:nP 3:1	45	265	83	50	3	1.31	27	V-1	[165]
**APP/PER/Zinc hydroxystannate (APP/PER/ZHS)**	25	P:nP:nP 3:1:0.16	40	193	75	50	3	1.77	32	V-0	[165]
	―	―	24	1361	107.5	50	3	―	―	―	[166]
**APP/PER**	25	P:nP 3:1	21	455	85.4	50	3	3.29	―	V-2	[166]
**APP/PER/Manganese acetate (APP/PER/MnAc)**	26	P:nP:nP 3:1:0.16	23	372	75.2	50	3	5.01	―	V-0	[166]
**APP/PER/MnAc**	27	P:nP:nP 3:1:0.32	19	366	74.1	50	3	4.27	―	V-0	[166]
**APP/PER/MnAc**	28	P:nP:nP 3:1:0.48	19	383	83.6	50	3	3.61	―	V-0	[166]
**APP/PER/MnAc**	29	P:nP:nP 3:1:0.64	18	369	96.1	50	3	3.09	―	V-0	[166]
	―	―	15	782	230	35	4	―	―	―	[167]
**APP/DPER/phosphorylated sodium alginate (APP/DPER/m-SA)**	35	P:nP:nP 3:1:1	27	335	128	35	4	7.55	―	―	[167]
	―	―	46	631.6	135.4	35	4	―	19	NR	[168]
**APP/PEPA**	23	P:nP 2:1	38	297.9	113.8	35	4	2.08	30.5	NR	[168]
**APP/PEPA/NOR116**	25	P:nP:nP 2:1:0.26	36	260.3	112.5	35	4	2.28	34	V-2	[168]
**APP/PEPA/Zirconium phosphate (APP/PEPA/ZrP)**	25	P:nP:nP 2:1:0.26	41	221.8	112.5	35	4	3.05	31.5	V-2	[168]
**APP/PEPA/Macromolecular N-alkoxy hindered amine functionalized ZrP (APP/PEPA/m-ZrP)**	25	P:nP:nP 2:1:0.26	40	157	112.2	35	4	4.22	33	V-0	[168]
	―	―	27	1474	142	50	3	―	18	NR	[169]
**MCAPP/PEPA**	25	P:P 2:1	18	438	123	50	3	2.59	31.1	V-2	[169]
**MCAPP/PEPA/Kaol**	25	P:P:nP 2:1:0.2	17	373	123	50	3	2.87	32.5	V-0	[169]
**MCAPP/PEPA/Acidically modified kaol (MCAPP/PEPA/m-Kaol)**	25	P:P:nP 2:1:0.2	20	233	105	50	3	6.33	34.9	V-0	[169]
	―	―	27	1474	142	50	3	―	18.1	NR	[170]
**MCAPP/PEPA**	25	P:P 2:1	18	438	123	50	3	2.59	31.1	V-2	[170]
**MCAPP/PEPA/Kaol**	25	P:P:nP 2:1:0.2	17	372	123	50	3	2.88	32.5	V-0	[170]
**MCAPP/PEPA/Thiourea modified kaol (MCAPP/PEPA/m-Kaol)**	25	P:P:nP 2:1:0.2	21	291	103	50	3	5.43	35.4	V-0	[170]
	―	―	27	1474	142	50	3	―	18	NR	[171]
**MCAPP/PEPA**	25	P:P 2:1	18	438	123	50	3	2.59	31.1	V-2	[171]
**MCAPP/PEPA/Kaol**	25	P:P:nP 2:1:0.2	17	373	123	50	3	2.87	32.5	V-0	[171]
**MCAPP/PEPA/Kaol nanoroll (MCAPP/PEPA/Kaol nanoroll)**	25	P:P:nP 2:1:0.2	19	269	120	50	3	4.56	34.5	V-0	[171]
	―	―	27	1474	142	50	―	―	18	NR	[89]
**MCAPP/PEPA**	25	P:nP 2:1	18	438	123	50	―	2.59	31.1	V-2	[89]
**MCAPP/PEPA/Kaol**	25	P:P:nP 2:1:0.2	17	373	123	50	―	2.87	32.5	V-0	[89]
**MCAPP/PEPA/Ammonium sulfamate intercalated kaol (MCAPP/PEPA/m-Kaol)**	25	P:P:nP 2:1:0.2	18	309	125	50	―	3.61	35.3	V-0	[89]
	―	―	27	1474	142	50	3	―	18	NR	[172]
**Microcapsulated APP/PEPA** **(mc-APP/PEPA)**	25	P:P 2:1	18	438	123	50	3	2.59	31	V-2	[172]
**Microcapsulated APP/PEPA/Kaol** **(mc-APP/PEPA/Kaol)**	25	P:P:nP 2:1:0.2	17	373	123	50	3	2.87	32.5	V-0	[172]
**Microcapsulated APP/PEPA/HNT** **(mc-APP/PEPA/HNT)**	25	P:P:nP 2:1:0.2	18	341	109	50	3	3.75	35.2	V-0	[172]
**Microcapsulated APP/PEPA/Kaol/HNT (mc-APP/PEPA/Kaol/HNT)**	25	P:P:nP:nP 2:1:0.18:0.2	19	263	97	50	3	5.77	36.9	V-0	[172]
	―	―	27	1474	142	50	3	―	18	NR	[173]
**Microcapsulated APP/PEPA** **(mc-APP/PEPA)**	25	P:P 2:1	18	436	123	50	3	2.60	31.2	V-2	[173]
**Microcapsulated APP/PEPA/Kaol** **(mc-APP/PEPA/Kaol)**	25	P:P:nP 2:1:0.2	17	374	122	50	3	2.88	32.5	V-2	[173]
**Microcapsulated APP/PEPA/HSA-A** **(mc-APP/PEPA/HSA-A)**	25	P:P:nP 2:1:0.2	23	299	106	50	3	5.62	34.1	V-0	[173]
**Microcapsulated APP/PEPA/HSA-P** **(mc-APP/PEPA/HSA-P)**	25	P:P:nP 2:1:0.2	20	257	84	50	3	7.18	35.1	V-0	[173]
**Microcapsulated APP/PEPA/ HSA-A-La (mc-APP/PEPA/HSA-A-La)**	25	P:P:nP 2:1:0.2	16	248	103	50	3	4.85	35.5	V-0	[173]
**Microcapsulated APP/PEPA/HSA-P-La** **(mc-APP/PEPA/HSA-P-La)**	25	P:P:nP 2:1:0.2	17	212	82	50	3	7.58	37.5	V-0	[173]
	―	―	38	1284	214	50	6	―	18.2	NR	[49]
**APP/Phosphorus based CA: 3,9-Bis-(1-oxo-2,6,7-trioxa-1-phospha-bicyclo[2,2,2]oct-4-ylmethoxy)-2,4,8,10-tetraoxa-3,9 diphospha-spiro[5.5]undecane 3,9-dioxide** **(APP/P-CA)**	25	P:P 1:1	31	318	161	50	6	4.37	30.5	V-0	[49]
	―	―	37	1284	121	50	3	―	―	―	[38]
**APP/Phosphorus-based FR: Cyclotriphosphazene containing six (aminopropyl)-triethoxysilicone groups (APP/P-FR)**	30	P:P 14:1	18	596	114	50	3	1.11	22.2	NR	[38]
**APP/Phosphorus-based FR: Cyclotriphosphazene containing six (aminopropyl)-triethoxysilicone groups (APP/P-FR)**	30	P:P 6.5:1	17	420	109	50	3	1.55	22.4	NR	[38]
**APP/Phosphorus-based FR: Cyclotriphosphazene containing six (aminopropyl)-triethoxysilicone groups (APP/P-FR)**	30	P:P 4:1	18	382	95	50	3	2.08	23.5	V-2	[38]
**APP/Phosphorus based FR: Cyclotriphosphazene containing six (aminopropyl)-triethoxysilicone groups (APP/P-FR)**	30	P:P 2.751	17	282	95	50	3	2.66	26.5	V-2	[38]
	―	―	33	1238	123.7	50	3	―	17.8	NR	[41]
**Melamine-formaldehyde-tris(2-hydroxyethyl) isocyanurate resin microencapsulated APP/Tris(2-hydroxyethyl) isocyanurate** **(mc-APP/THEIC)**	30	P:nP 3:1	28	232	100.7	50	3	5.56	36	V-0	[41]
	―	―	42	831	112	35	3	―	18	NR	[50]
**APP/Polyurethane containing phosphorus-based CA (APP/PPU-CA)**	25	P:nP 2:1	19.8	232	69	35	3	2.74	24.5	V-2	[50]
**APP/PPU-CA**	25	P:N 1:1	17.1	288	70	35	3	1.87	25.5	V-1	[50]
	―	―	37	1677	184.4	50	4	―	17	NR	[95]
**APP/Triazine-based CFA (APP/TA-CFA)**	22	P:nP 4:1	21	397.3	161.1	50	4	2.74	30.4	V-0	[95]
**(3-Aminopropyl) triethoxysilane modified APP microcapsulated with methylpolysiloxane/Triazine-based CFA** **(m-APP/TA-CFA)**	22	P:nP 4:1	16	271.7	140.8	50	4	3.49	31.7	V-0	[95]
	―	―	20	809	96	50	3	―	17.6	NR	[32]
**APP/Triazine based CFA: Poly[N4-bis(ethylenediamino)-phenyl phosphonic-N2, N6-bis(ethylenediamino)-1,3,5-triazine-N-phenyl (APP/TA-CFA)**	25	P:nP 2:1	11	121	81	50	3	4.35	34	V-0	[32]
	―	―	45	759.2	98.8	35	3	―	17	NR	[37]
**APP/Triazine based CFA: synthesized from a macromolecular triazine derivative containing hydroxyethylamino and triazine rings and ethylenediamino groups** **(APP/TA-CFA)**	25	P:nP 4:1	40	167.6	82.5	35	3	4.82	34	V-0	[37]
**Melamine and phytic acid modified APP/Triazine-based CFA: synthesized from a macromolecular triazine derivative containing hydroxyethylamino and triazine rings and ethylenediamino groups** **(m-APP/TA-CFA)**	25	P:nP 4:1	43	115.6	82.3	35	3	7.53	35	V-0	[37]
	―	―	50	1350	91.2	35	3	―	17	NR	[40]
**APP/Triazine-based CFA: synthesized by polycondensation of 2-chloro-4,6-di-(2-hydroxyethylamino)-s-triazine** **(APP/TA-CFA)**	30	P:nP 2:1	56	422	70.7	35	3	4.62	32.5	V-0	[40]
**APP/Triazine-based CFA: synthesized by polycondensation of 2-chloro-4,6-di-(2-hydroxyethylamino)-s-triazine** **(APP/TA-CFA)**	30	P:nP 3:1	48	316	68.8	35	3	5.43	33	V-0	[40]
**APP/Triazine-based CFA: synthesized by polycondensation of 2-chloro-4,6-di-(2-hydroxyethylamino)-s-triazine** **(APP/TA-CFA)**	30	P:nP 4:1	52	414	71.1	35	3	4.35	31.5	V-0	[40]
	―	―	48	988	88.3	35	3	―	17	NR	[39]
**APP/Triazine-based CFA: synthesized by reaction of cyanuric chloride and piperazine (APP/TA-CFA)**	30	P:N 1:1	32	82.4	77.9	35	3	9.06	29	V-0	[39]
**APP/Triazine-based CFA: synthesized by reaction of cyanuric chloride and piperazine (APP/TA-CFA)**	30	P:nP 2:1	52	94.2	78.4	35	3	12.79	32	V-0	[39]
**APP/Triazine-based CFA: synthesized by reaction of cyanuric chloride and piperazine (APP/TA-CFA)**	30	P:nP 3:1	34	167	83.4	35	3	4.43	34	V-0	[39]
**APP/Triazine-based CFA: synthesized by reaction of cyanuric chloride and piperazine (APP/TA-CFA)**	30	P:nP 4:1	56	163.6	67.9	35	3	9.16	29.5	V-0	[39]
	―	―	48	906	90.3	35	3.2	―	17	NR	[174]
**APP/Triazine-based CFA: synthesized by reaction of cyanuric chloride and piperazine (APP/TA-CFA)**	20	P:nP 3:1	34	143	60.1	35	3.2	6.74	29	V-1	[174]
**APP/Triazine-based CFA: synthesized by reaction of cyanuric chloride and piperazine /hexadecyl trimethyl ammonium bromide modified MMT (APP/TA-CFA/m-MMT)**	20	P:nP:nP 3:1:0.1	36	132	58.7	35	3.2	7.91	31	V-0	[174]
**APP/Triazine-based CFA: synthesized by reaction of cyanuric chloride and piperazine /hexadecyl trimethyl ammonium bromide modified MMT (APP/TA-CFA/m-MMT)**	20	P:nP:nP 3:1:0.2	36	90	58.6	35	3.2	11.63	30.5	V-0	[174]
**APP/Triazine-based CFA: synthesized by reaction of cyanuric chloride and piperazine /hexadecyl trimethyl ammonium bromide modified MMT (APP/TA-CFA/m-MMT)**	20	P:nP:nP 3:1:0.3	32	52.6	35.5	35	3.2	29.20	30.5	V-0	[174]
**APP/Triazine-based CFA: synthesized by reaction of cyanuric chloride and piperazine /hexadecyl trimethyl ammonium bromide modified MMT (APP/TA-CFA/m-MMT)**	20	P:nP:nP 3:1:0.5	38	55.2	38.5	35	3.2	30.47	31.5	V-0	[174]
**APP/Triazine-based CFA: synthesized by reaction of cyanuric chloride and piperazine /hexadecyl trimethyl ammonium bromide modified MMT (APP/TA-CFA/m-MMT)**	20	P:nP:nP 3:1:0.7	36	112.7	41.4	35	3.2	13.15	30.5	NR	[174]
	―	―	34	1052	90.8	50	3	―	17.5	NR	[61]
**APP/Triazine-based CFA: synthesized by reaction of cyanuric chloride and ethanolamine and ethylenediamine/Silicon dioxide (APP/TA-CFA/SiO_2_)**	24	P:nP:nP 4:1:0.26	30	236.6	84.7	50	3	4.20	36.4	V-1	[61]
**APP/Triazine-based CFA: synthesized by reaction of cyanuric chloride and ethanolamine and ethylenediamine/AHP/SiO_2_ (APP/TA-CFA/AHP/SiO_2_)**	24	P:nP:nP:nP 4:1:0.86:0.2	33	221	83.2	50	3	5.04	35.8	V-0	[61]
	―	―	36	1153	130	50	―	―	17.5	NR	[175]
**APP/Triazine-based CFA: N-ethyl triazineepiperazine copolymer/SiO_2_ (APP/TA-CFA/SiO_2_)**	24	P:nP:nP 4:1:0.26	26	88	20	50	―	61.50	34.1	V-0	[175]
**APP/Triazine-based CFA: N-ethyl triazineepiperazine copolymer/SiO_2_ (APP/TA-CFA/SiO_2_)**	24	P:nP:nP 4:1:0.26	27	95	24	50	―	49.30	33.5	V-0	[175]
	―	―	50	1025	110.8	35	3	―	17	NR	[176]
**APP/Triazine-based CFA: synthesized by reaction of cyanuric chloride and ethanolamine and ethylenediamine** **(APP/TA-CFA)**	25	P:nP 4:1	36	213	90.5	35	3	4.24	34	V-0	[176]
**APP/Triazine-based CFA: synthesized by reaction of cyanuric chloride and ethanolamine and ethylenediamine/rGNO (APP/TA-CFA/rGNO)**	25	P:nP:nP 4:1:0.1	35	140	90.4	35	3	6.28	32	V-0	[176]
**APP/Triazine-based CFA: synthesized by reaction of cyanuric chloride and ethanolamine and ethylenediamine/rGNO (APP/TA-CFA/rGNO)**	25	P:nP:nP 4:1:0.2	34	156	86	35	3	5.75	28	V-0	[176]
**APP/Triazine-based CFA: synthesized by reaction of cyanuric chloride and ethanolamine and ethylenediamine/rGNO (APP/TA-CFA/rGNO)**	25	P:nP:nP 4:1:0.4	36	262	94.4	35	3	3.30	25	V-2	[176]
	―	―	45	1456	139.1	35	3	―	17	NR	[177]
**Piperazine modified APP/Triazine-based CFA: synthesized by reaction of cyanuric chloride and ethanolamine and ethylenediamine (m-APP/TA-CFA)**	25	P:nP 4:1	41	501	126	35	3	2.92	35	V-0	[177]
**Piperazine modified APP/Triazine based CFA: synthesized by reaction of cyanuric chloride and ethanolamine and ethylenediamine/rGNO** **(m-APP/TA-CFA/rGNO)**	25	P:nP:nP 4:1:0.1	39	434	125.1	35	3	3.23	34	V-0	[177]
**Piperazine modified APP/Triazine-based CFA: synthesized by reaction of cyanuric chloride and ethanolamine and ethylenediamine/rGNO** **(m-APP/TA-CFA/rGNO)**	25	P:nP:nP 4:1:0.2	37	350	123.9	35	3	3.84	32	V-0	[177]
**Piperazine modified APP/Triazine-based CFA: synthesized by reaction of cyanuric chloride and ethanolamine and ethylenediamine/rGNO** **(m-APP/TA-CFA/rGNO)**	25	P:nP:nP 4:1:0.4	37	397	125.4	35	3	3.34	30	V-0	[177]
**Piperazine modified APP/Triazine-based CFA: synthesized by reaction of cyanuric chloride and ethanolamine and ethylenediamine/Piperazine modified APP attached with rGNO** **(m-APP/TA-CFA/m-APP@rGNO)**	25	P:nP:nP 3.6:1:0.5	38	401	117.3	35	3	3.63	33	V-0	[177]
**Piperazine modified APP/Piperazine modified APP attached with rGNO/Triazine-based CFA: synthesized by reaction of cyanuric chloride and ethanolamine and ethylenediamine** **(m-APP/m-APP@rGNO/TA-CFA)**	25	P:nP:nP 3.04:1:0.96	34	290	117.1	35	3	4.50	30.5	V-0	[177]
**Piperazine modified APP/Piperazine modified APP attached with rGNO/Triazine-based CFA: synthesized by reaction of cyanuric chloride and ethanolamine and ethylenediamine** **(m-APP/m-APP@rGNO/TA-CFA)**	25	P:nP:nP 1.04:1:0.46	31	464	125.8	35	3	2.39	26.5	V-2	[177]
	―	―	66	633	44.2	35	3	―	17	NR	[23]
**APP/Triazine-based CA: synthesized by reaction of 2-carboxyethyl (phenyl) phosphinic acid and tris (2-hydrooxyethyl) isocyanurate (APP/TA-CA)**	20	P:N 1:1	38	83	41	35	3	4.73	30	V-0	[23]
	―	―	48	1351	107	35	3.2	―	18.5	NR	[27]
**APP/Triazine-based CA: synthesized by reaction of cyanuric chloride, 2,6,7-trioxa-1-phosphabicyclo [2,2,2]octane-4-methanol and piperazine (APP/TA-CA)**	20	P:nP 2:1	39	255	101	35	3.2	4.56	27.5	V-0	[27]
**APP/Triazine-based CA: synthesized by reaction of cyanuric chloride, 2,6,7-trioxa-1-phosphabicyclo [2,2,2]octane-4-methanol and piperazine (APP/TA-CA)**	20	P:nP 3:1	37	253	98	35	3.2	4.49	28	V-0	[27]
	―	―	41	840.3	115.7	35	3	―	16.4	NR	[67]
**APP/Triazine-based CA: Poly(ethanediamine-1,3,5-triazine-p-4-amino-2,2,6,6-tetramethylpiperidine) (APP/TA-CA)**	25	P:nP 2:1	28	227.9	62	35	3	4.69	30.3	V-0	[67]
	―	―	33	1416	219	50	6	―	17	NR	[36]
**APP/Triazin-based CA: poly(1,3,5-triazin-2-aminoethanol diethylenetriamine) (APP/TA-CA)**	25	P:nP 3:1	17	219	165	50	6	4.42	32.7	V-0	[36]
**Polysiloxane shell-coated APP/Triazin-based CA: poly(1,3,5-triazin-2-aminoethanol diethylenetriamine) (mc-APP/TA-CA)**	25	P:nP 3:1	18	176	82	50	6	11.72	35	V-0	[36]
	―	―	70	1267	147	50	3	―	17.5	NR	[149]
**APP/Triazine based CA: synthesized by reaction of cyanuric trichloride and diphenylamine and ethylenediamine (APP/TA-CA)**	30	P:nP 2:1	35	187	68	50	3	7.32	31.5	V-0	[149]
	―	―	25	1239	123.6	50	3	―	18.5	NR	[178]
**APP/Triazine-based CA: Tris(2-hydroxyethyl) isocyanurate (APP/TA-CA)**	30	P:nP 2:1	18	253.7	91.2	50	3	4.76	32.8	V-0	[178]
**APP/Triazine-based CA: Tris(2-hydroxyethyl) isocyanurate homopolymer (APP/Homo-TA-CA)**	30	P:nP 2:1	18	349.9	91.3	50	3	3.45	34.6	V-0	[178]
	―	―	47	860.6	110.3	35	3	―	18	NR	[179]
**APP/Triazine-based CA: synthesized by reaction of cyanuric chloride and 2,6,7-trioxa-l-phosphabicyclo[2,2,2]octane-4-methanol and diethylenetriamine (APP/TA-CA)**	30	P:N 1:1	31	136.5	81	35	3	5.66	32	V-0	[179]
**APP/Triazine-based CA: synthesized by reaction of cyanuric chloride and 2,6,7-trioxa-l-phosphabicyclo[2,2,2]octane-4-methanol and diethylenetriamine (APP/TA-CA)**	30	P:nP 4:1	35	257.6	80.8	35	3	3.39	35.5	V-0	[179]
	―	―	36	799	170	35	4	―	17.5	NR	[73]
**APP/Triazine-based CA: synthesized by reaction of Cyanuric chloride and Ethanedi-amine and γ-Aminopropyl triethoxysilane (APP/TA-CA)**	25	P:nP 2:1	25	180	131	35	4	4.00	35.5	V-0	[73]
**APP/Triazine-based CA: synthesized by reaction of Cyanuric chloride and Ethanedi-amine and γ-Aminopropyl triethoxysilane/ NOR116 (APP/TA-CA/NOR116)**	25	P:nP:nP 2:1:0.03	32	76	122	35	4	13.02	42.5	V-0	[73]
	―	―	55	818	213	35	6	―	―	―	[180]
**APP/Triazine-based CA: synthesized by polycondensation of 2-amino-4,6-dichloro-s-triazines and diethylenetriamine** **(APP/TA-CA)**	20	P:nP 2.8:1	35	218	126	35	6	4.03	30.8	V-1	[180]
**APP/Triazine-based CA: synthesized by polycondensation of 2-amino-4,6-dichloro-s-triazines and diethylenetriamine/Organically modified MMT (APP/TA-CA/m-MMT)**	20	P:nP:nP 2.8:1:0.2	34	159	64	35	6	10.58	33	V-0	[180]
**APP/Triazine-based CA: synthesized by polycondensation of 2-amino-4,6-dichloro-s-triazines and diethylenetriamine/Organically modified MMT (APP/TA-CA/m-MMT)**	20	P:nP:nP 2.8:1:0.6	37	270	156	35	6	2.78	28.9	NR	[180]
	―	―	29	1603	133	50	4	―	17.5	NR	[181]
**APP/Triazine-based CFA: N-methyl triazineethylenediamine copolymer/SiO_2_ (APP/TA-CFA/SiO_2_)**	22	P:nP:nP 4:1:0.26	16	91	62	50	4	20.84	29.6	V-0	[181]
**APP/Triazine-based CFA: N-methyl triazineethylenediamine copolymer/SiO_2_ (APP/TA-CFA/SiO_2_)**	22	P:nP:nP 4:1:0.26	22	74	69	50	4	31.67	29.3	V-0	[181]
	―	―	18	1457	156	50	3	―	19	NR	[29]
**APP/Triazine-based CA: synthesized by reaction of cyanuric chloride and γ-aminopropyltriethoxy silane and trimethylamine and ethylenediamine-Zinc oxide (APP/TA-CA-ZnO)**	25	P:nP 2:1	19	191	112	50	3	11.21	31.1	V-0	[29]
**APP/Triazine-based CA: synthesized by reaction of cyanuric chloride and γ-aminopropyltriethoxy silane and trimethylamine and ethylenediamine/ZnO (APP/TA-CA/ZnO)**	25	P:nP:nP 2.25:1:0.12	18	430	132	50	3	4.00	26.1	V-1	[29]
	―	―	62	1221	265	35	6	―	19	NR	[182]
**APP and Triazine-based IFR (APPandTA-IFR)**	10	P:N ―	44	313	235	35	6	3.12	26	NR	[182]
**APP and Triazine-based IFR (APPandTA-IFR)**	15	P:N ―	45	148	191	35	6	8.30	29	NR	[182]
**APP and Triazine-based IFR (APPandTA-IFR)**	20	P:N ―	43	115	153	35	6	12.75	31	V-0	[182]
	―	―	48	988	88.3	35	3.2	―	17	NR	[31]
**APP/Triazin-based IFR: synthesized by reaction of cyanuric chloride** **and N-amino ethylpiperazine (APP/TA-IFR)**	25	P:N 1:1	44	123	73.3	35	3.2	8.86	27.5	V-0	[31]
**APP/Triazin-based IFR: synthesized by reaction of cyanuric chloride** **and N-amino ethylpiperazine (APP/TA-IFR)**	25	P:nP 2:1	38	117	23.2	35	3.2	25.44	29.5	V-0	[31]
**APP/Triazin-based IFR: synthesized by reaction of cyanuric chloride** **and N-amino ethylpiperazine (APP/TA-IFR)**	25	P:nP 3:1	36	113	73.9	35	3.2	7.83	30.5	V-0	[31]
	―	―	21	1242	111	50	3.2	―	18.6	NR	[33]
**APP/Piperazine-Triazine-based CA: synthesized by reaction of cyanuric chloride and anhydrous piperazine (APP/PI-TI-CA)**	25	P:nP 4:1	22	330	103	50	3.2	4.24	34.1	V-0	[33]
**APP/Piperazine-Triazine-based CA cluster: synthesized by reaction of cyanuric chloride and anhydrous piperazine (APP/PI-TI-CA)**	25	P:nP 4:1	21	242	96	50	3.2	5.93	33.9	V-0	[33]
	―	―	45	1269	146.35	50	3	―	17.5	NR	[70]
**APP/Piperazine-based IFR: Piperazine spirocyclic phosphoramidate (APP/PI-IFR)**	30	P:nP 3:1	23	208.8	81.41	50	3	5.58	32.5	V-0	[70]
**APP/Piperazine-based IFR: Piperazine spirocyclic phosphoramidate/Triazine based CFA (APP/PI-IFR/TA-CFA)**	30	P:nP:nP 2:1:0.65	23	116.1	41.57	50	3	19.66	39.8	V-0	[70]
	―	―	76	455	102	35	3	―	17	NR	[183]
**APP/ATH**	30	P:M 1:1	40	210	75	35	3	1.55	24	V-1	[183]
**4,4′-diphenylmethane diisocyanateandmelamine co-microencapsulated APP and ATH** **(mc-(APPandATH))**	30	P:M 1:1	75	120	53	35	3	7.20	25.5	V-0	[183]
	―	―	14	1104	106	35	0.4	―	―	―	[24]
**APP/MMT**	10	P:nP 4:1	25	769	66	35	0.4	4.11	―	―	[24]
**APP/MMT**	10	P:nP 1.5:1	27	765	65	35	0.4	4.53	―	―	[24]
**APP/Modified MMT (APP/m-MMT)**	10	P:nP 4:1	29	715	64	35	0.4	5.29	―	―	[24]
**APP/Modified MMT (APP/m-MMT)**	10	P:nP 1.5:1	30	619	63	35	0.4	6.43	―	―	[24]
	―	―	50.2	789	156.6	35	―	―	17.5	―	[103]
**APP/Nf**	20	P:nP 3:1	40.8	399	167.9	35	―	1.49	23	―	[103]
**APP/organically modified BT (APP/m-BT)**	20	P:nP 3:1	42.6	386	155.1	35	―	1.75	23	―	[103]
	―	―	33	847	159.8	50	―	―	17.5	―	[103]
**APP/Nf**	20	P:nP 3:1	29	426	168.2	50	―	1.66	23	―	[103]
**APP/organically modified BT (APP/m-BT)**	20	P:nP 3:1	24	445	150	50	―	1.47	23	―	[103]
	―	―	44	1172	87.1	35	2.5	―	18.1	NR	[60]
**APP/C15A**	20	P:nP 3:1	61	490	72.7	35	2.5	3.97	20.1	NR	[60]
**OP/C15A**	20	P:nP 3:1	50	400	83.6	35	2.5	3.46	20.8	NR	[60]
	―	―	35	1622	103	35	3	―	―	―	[104]
**APP/C20A/PER/MEL**	25	P:nP:nP:nP 2.4:1:0.8:0.8	26	403	93	35	3	3.31	―	―	[104]
**APPandMMT/C20A/PER/MEL**	25	P:nP:nP:nP 2.4:1:0.8:0.8	23	385	80	35	3	3.56	―	―	[104]
**APPandMMT/C20A/PER/MEL**	15	P:nP:nP:nP 1.2:1:0.4:0.4	18	460	86	35	3	2.17	―	―	[104]
**APPandMMT/C20A/PER/MEL**	20	P:nP:nP:nP 1.8:1:0.6:0.6	18	411	86	35	3	2.43	―	―	[104]
	―	―	34	1294	154.2	50	4	―	19	NR	[26]
**APP/Phytic acid modified LDH** **(APP/m-LDH)**	20	P:nP 19:1	20	200	59.3	50	4	9.89	―	V-1	[26]
**APP/Phytic acid modified LDH** **(APP/m-LDH)**	20	P:nP 9:1	19	291	144.3	50	4	2.65	―	V-0	[26]
**APP/Phytic acid modified LDH** **(APP/m-LDH)**	20	P:nP 5.6:1	18	327	150.8	50	4	2.14	―	V-1	[26]
	―	―	36	1373	174.8	50	3	―	18.5	NR	[51]
**APP based IFR/Mg-Al LDH (APP-IFR/LDH)**	20	P:nP 9:1	18	286	108.8	50	3	3.85	32.5	V-0	[51]
**APP-based IFR/Mg-Al LDH (APP-IFR/LDH)**	20	P:nP 4:1	15	326	120.3	50	3	2.54	―	NR	[51]
**APP-based IFR/Mg-Zn-Al LDH** **(APP-IFR/LDH)**	20	P:nP 9:1	16	306	136	50	3	2.56	―	V-0	[51]
**APP-based IFR/Mg-Zn-Al LDH** **(APP-IFR/LDH)**	20	P:nP 4:1	14	265	90.4	50	3	3.89	―	V-0	[51]
	―	―	25	981	147	50	―	―	17.6	NR	[30]
**APP/Polysiloxane-based FR (APP/Si-FR)**	25	P:M 1:1	14	277	97	50	―	3.00	28.9	V-0	[30]
**APP/Polysiloxane-based FR (APP/Si-FR)**	25	P:nP 1.5:1	15	168	91	50	―	5.65	29.8	V-0	[30]
**APP/Polysiloxane-based FR (APP/Si-FR)**	25	P:nP 1.5:1	14	238	98	50	―	3.46	28.2	V-0	[30]
**APP/Polysiloxane-based FR (APP/Si-FR)**	25	P:nP 2:1	18	245	93	50	―	4.55	29.1	V-0	[30]
**APP/Polysiloxane-based FR (APP/Si-FR)**	25	P:nP 3:1	20	197	98	50	―	5.97	28.4	V-0	[30]
	―	―	54	1610	106	35	3	―	20.8	NR	[25]
**APP/SEP**	12.5	P:nP 24:1	27	320	88	35	3	3.03	25.7	NR	[25]
**APP/Organically modified SEP** **(APP/m-SEP)**	12.5	P:nP 24:1	28	241	92	35	3	3.99	26.3	V-0	[25]
	―	―	36	1153	130	50	―	―	17.5	NR	[175]
**APP/SiO_2_**	24	P:nP 19:1	30	502	151	50	―	1.64	―	―	[175]
	―	―	35	1203	197.6	50	6	―	18.2	NR	[34]
**APP/CB**	25	P:nP 7.3:1	32	343.6	157.2	50	6	4.02	28.3	NR	[34]
**APP/CB**	25	P:nP 4:1	31	316.2	136.9	50	6	4.86	29.1	V-1	[34]
**APP/CB**	25	P:nP 2.5:1	33	302.1	121.4	50	6	6.11	29.8	V-0	[34]
	―	―	46	1541	90	35	3	―	―	―	[135]
**APP/CD**	15	P:Bio 1:1	24	910	89	35	3	0.89	―	―	[135]
	―	―	38	1284	214	50	6	―	18.2	NR	[184]
**Phosphorus based CA: 3,9-Bis-(1-oxo-2,6,7-trioxa-1-phospha-bicyclo[2,2,2]oct-4-ylmethoxy)- 2,4,8,10-tetraoxa-3,9-diphospha-spiro[5.5]undecane 3, 9-dioxide/MEL (P-CA/MEL)**	30	P:nP 4:1	18	198	175	50	6	3.75	31.6	V-0	[184]
	―	―	65	1417	128.5	35	3	―	―	NR	[57]
**Phosphorus based FR: Tri (1-oxo-2,6,7-trioxa-1-phosphabicyclo [2,2,2] octane-methyl) phosphate/MPyP (P-FR/MPyP)**	30	P:N 1:1	40	175.2	90.6	35	3	7.05	―	V-0	[57]
	―	―	54	1199	97.8	35	―	―	―	―	[58]
**Phosphorus-based FR: Poly(4,4-diaminodiphenyl methane spirocyclicpentaerythritol bisphosphonate)/GNO (P-FR/GNO)**	20	P:nP 10:1	64	473	79	35	―	3.71	―	―	[58]
	―	―	30	390	44	35	1.6	―	17.4	―	[53]
**Phosphorus-based IFR: Poly (4,4-diamino diphenyl methane Obicyclicpentaerythritol phosphate-phosphate)/Znacac (P-IFR/Znacac)**	20	P:nP 19:1	23	175	25	35	1.6	3.00	27.4	―	[53]
**Phosphorus-based IFR: Poly (4,4-diamino diphenyl methane Obicyclicpentaerythritol phosphate-phosphate)/Cracac** **(P-IFR/Cracac)**	20	P:nP 19:1	24	184	24	35	1.6	3.10	28.2	―	[53]
	―	―	29	980	136	50	―	―	18.5	―	[56]
**Phosphorus and Nitrogene-based IFR: compound containing Phosphorus and Nitrogen /Dioctadecyl dimethyl ammonium chloride modified MMT** **(PN-IFR/m-MMT)**	30	P:nP 59:1	20	173	102	50	―	5.20	38.5	―	[56]
**Phosphorus and Nitrogene-based IFR: compound containing Phosphorus and Nitrogen /Dioctadecyl dimethyl ammonium chloride modified MMT** **(PN-IFR/m-MMT)**	30	P:nP 29:1	22	192	127	50	―	4.14	38	―	[56]
**Phosphorus and Nitrogene-based IFR: compound containing Phosphorus and Nitrogen /Dioctadecyl dimethyl ammonium chloride modified MMT** **(PN-IFR/m-MMT)**	30	P:nP 19:1	21	173	134	50	―	4.16	37.7	―	[56]
**Phosphorus and Nitrogene-based IFR: compound containing Phosphorus and Nitrogen /Dioctadecyl dimethyl ammonium chloride modified MMT** **(PN-IFR/m-MMT)**	30	P:nP 14:1	22	176	133	50	―	4.31	35.7	―	[56]
**Phosphorus and Nitrogene-based IFR: compound containing Phosphorus and Nitrogen /Dioctadecyl dimethyl ammonium chloride modified MMT** **(PN-IFR/m-MMT)**	30	P:nP 11:1	20	135	131	50	―	5.19	35.8	―	[56]
**Phosphorus and Nitrogene-based IFR: compound containing Phosphorus and Nitrogen /Dioctadecyl dimethyl ammonium chloride modified MMT** **(PN-IFR/m-MMT)**	30	P:nP 9:1	21	155	134	50	―	4.64	31	―	[56]
**Phosphorus and Nitrogene-based IFR: compound containing Phosphorus and Nitrogen /Dioctadecyl dimethyl ammonium chloride modified MMT** **(PN-IFR/m-MMT)**	30	P:n P6.5:1	10	202	132	50	―	1.72	28.5	―	[56]
**Phosphorus and Nitrogene-based IFR: compound containing Phosphorus and Nitrogen /Dioctadecyl dimethyl ammonium chloride modified MMT** **(PN-IFR/m-MMT)**	30	P:nP 5:1	19	202	133	50	―	3.25	25.5	―	[56]
	―	―	37	363	56	35	3	―	―	―	[55]
**Phosphorus-based IFR: compound containing Phosphorus(22%) and Nitrogene(18%)/Octadecyl trimethyl ammonium bromide modified MMT** **(P-IFR/m-MMT)**	28	P:nP 10.2:1	31	45	18	35	3	21.02	―	―	[55]
**Phosphorus-based IFR: compound containing Phosphorus(22%) and Nitrogene(18%)/Sodium dodecyl sulfonate intercalated Ni-Al LDH (P-IFR/m-LDH)**	28	P:nP 10.2:1	30	64	20	35	3	12.87	―	―	[55]
**Phosphorus-based IFR: compound containing Phosphorus(22%) and Nitrogene(18%)/A-POSS (P-IFR/A-POSS)**	28	P:nP 10.2:1	32	55	16	35	3	19.97	―	―	[55]
**Phosphorus-based IFR: compound containing Phosphorus(22%) and Nitrogene(18%)/MWCNT (P-IFR/MWCNT)**	28	P:nP 10.2:1	33	145	54	35	3	2.31	―	―	[55]
	―	―	37	363	56	35	3	―	―	―	[54]
**Phosphorus-based IFR: compound containing Phosphorus(22%) and Nitrogene(18%)/MWCNT (P-IFR/MWCNT)**	28	P:nP 17.6:1	33	225	73	35	3	1.10	―	―	[54]
**Phosphorus-based IFR: compound containing Phosphorus(22%) and Nitrogene(18%)/MWCNT (P-IFR/MWCNT)**	28	P:nP 10.2:1	33	145	54	35	3	2.31	―	―	[54]
**Phosphorus-based IFR: compound containing Phosphorus(22%) and Nitrogene(18%)/MWCNT (P-IFR/MWCNT)**	28	P:nP 7:1	31	140	79	35	3	1.53	―	―	[54]
	―	―	24.7	1198.2	78.7	50	2.4	―	―	NR	[139]
**Phosphoric acid/ethylenediamine (PA/EDA)**	25	P:nP 1.7:1	12.8	263.1	57.1	50	2.4	3.25	―	V-0	[139]
	―	―	42	831	112	35	3	―	18	NR	[50]
**PPU-CA/APP**	25	N:P 1:1	17.1	288	70	35	3	1.87	25.5	V-1	[50]
**PPU-CA/APP**	25	N:nN 2:1	19.7	568	75	35	3	1.02	25.5	V-0	[50]
**PPU-CA/APP**	25	N:nN 3:1	26.8	605	77	35	3	1.27	26.5	V-0	[50]
**PPU-CA/APP**	25	N:nN 4:1	22.2	642	77	35	3	0.99	27	V-0	[50]
	―	―	54	930	140	35	4	―	―	NR	[64]
**MP/PER**	25	N:nN 1.5:1	28	250	99	35	4	2.72	―	V-0	[64]
**MP/PER/Kaol**	25	N:nN:nN 1.66:1:0.11	35	305	110	35	4	2.51	―	V-0	[64]
**MP/PER/Kaol**	25	N:nN:nN 1.87:1:0.25	37	400	114	35	4	1.95	―	NR	[64]
	―	―	42	1290	228	50	6	―	18.1	NR	[185]
**MP/PER**	20	N:nN 1.6:2	25	380	212	50	6	2.17	29	V-2	[185]
**MP/PER**	25	N:nN 1.6:2	24	265	206	50	6	3.07	34	V-0	[185]
**MP/PER/Organically modified MMT (MP/PER/m-MMT)**	20	N:nN:nN 1.6:2:0.13	23	228	207	50	6	3.41	31.5	V-0	[185]
**MP/PER/Organically modified MMT (MP/PER/m-MMT)**	25	N:nN:nN 1.6:2:0.1	24	197	205	50	6	4.16	35	V-0	[185]
	―	―	51	903	119.6	35	3	―	17.5	NR	[186]
**MMP/PER**	26	N:nN 1.7:1	40	308	99	35	3	2.77	27.5	V-2	[186]
**MMP/PER/Lanthanum oxide (MMP/PER/La_2_O_3_)**	26	N:nN:nN 1.7:1:0.1	45	271	94.3	35	3	3.72	32	V-0	[186]
**MMP/PER/La_2_O_3_**	26	N:nN:nN 1.7:1:0.23	47	247	91.4	35	3	4.40	31.5	V-0	[186]
**MMP/PER/La_2_O_3_**	26	N:nN:nN 1.7:1:0.36	50	221	85	35	3	5.63	31.5	V-0	[186]
	―	―	38	1166	89.1	35	3	―	17	NR	[187]
**MPP/DPER**	30	N:nN 3:1	45	427.6	79.4	35	3	3.62	28.7	V-0	[187]
**MPP/EG/DPER**	30	N:nN:nN 1.5:1:0.5	20	218.2	68.7	35	3	3.64	33.2	V-0	[187]
	―	―	25	1239	123.6	50	3	―	18.5	NR	[143]
**Amino trimethylene phosphonic acid melamine salt/PER (MATMP/PER)**	25	N:nN 2:1	17	256.4	103.2	50	3	3.93	30.3	V-0	[143]
	―	―	54	961	175	35	4	―	―	―	[188]
**MPyP/PER**	25	N:nN 3:1	32	343	136	35	4	2.13	29	V-1	[188]
**MPyP/PER/Epoxy crosslinked β-cyclodextrin nanosponge** **(MPyP/PER/m-CD)**	25	N:nN:nN 3:1:0.35	30	235	118	35	4	3.36	32.5	V-0	[188]
	―	―	65	1417	128.5	35	3	―	―	NR	[57]
**MPyP/Phosphorus-based FR: Tri (1-oxo-2,6,7-trioxa-1-phosphabicyclo [2,2,2] octane-methyl) phosphate (MPyP/P-FR)**	30	N:P 1:1	40	175.2	90.6	35	3	7.05	―	V-0	[57]
	―	―	34	1727	112	35	3	―	17	NR	[66]
**MPyP/Triazine-based CFA: synthesized by reaction of cyanuric chloride and ethanolamine and ethylenediamine** **(MPyP/TA-CFA)**	30	N:N 3:1	12	431	84	35	3	1.88	29.5	V-0	[66]
**MPyP/Triazine-based CFA: synthesized by reaction of cyanuric chloride and ethanolamine and ethylenediamine** **(MPyP/TA-CFA)**	30	N:N 1:1	15	469	85	35	3	2.14	31.2	V-0	[66]
**MPyP/Triazine based CFA: synthesized by reaction of cyanuric chloride and ethanolamine and ethylenediamine** **(MPyP/TA-CFA)**	30	N:N 3:1	12	525	92	35	3	1.41	26.8	NR	[66]
	―	―	48	988	88.3	35	3	―	17	NR	[39]
**Triazine-based CFA: synthesized by reaction of cyanuric chloride and piperazine/APP (TA-CFA/APP)**	30	N:P 1:1	32	82.4	77.9	35	3	9.06	29	V-0	[39]
**Triazine-based CFA: synthesized by reaction of cyanuric chloride and piperazine/APP (TA-CFA/APP)**	30	N:nN 2:1	52	247	78.4	35	3	4.88	23	V-1	[39]
	―	―	66	633	44.2	35	3	―	17	NR	[23]
**Triazine-based CA: synthesized by reaction of 2-carboxyethyl (phenyl) phosphinic acid and tris (2-hydrooxyethyl) isocyanurate/APP (TA-CA/APP)**	20	N:P 1:1	38	83	41	35	3	4.73	30	V-0	[23]
	―	―	48	1351	107	35	3.2	―	18.5	NR	[27]
**Triazine-based CA: synthesized by reaction of cyanuric chloride, 2,6,7-trioxa-1-phosphabicyclo [2,2,2]octane-4-methanol and piperazine/APP (TA-CA/APP)**	20	N:nN 2:1	36	456	96	35	3.2	2.47	25.5	V-2	[27]
	―	―	47	860.6	110.3	35	3	―	18	NR	[179]
**Triazine-based CA: synthesized by reaction of cyanuric chloride and 2,6,7-trioxa-l-phosphabicyclo[2,2,2]octane-4-methanol and diethylenetriamine/APP (TA-CA/APP)**	30	N:nN 2:1	32	166.8	108.2	35	3	3.58	28	V-0	[179]
**Triazine-based CA: synthesized by reaction of cyanuric chloride and 2,6,7-trioxa-l-phosphabicyclo[2,2,2]octane-4-methanol and diethylenetriamine/APP (TA-CA/APP)**	30	N:P 1:1	31	136.5	81	35	3	5.66	32	V-0	[179]
	―	―	48	988	88.3	35	3.2	―	17	NR	[31]
**Triazin-based IFR: synthesized by reaction of cyanuric chloride and N-amino ethylpiperazine/APP (TA-IFR/APP)**	25	N:P 1:1	44	123	73.3	35	3.2	8.86	27.5	V-0	[31]
**Triazin-based IFR: synthesized by reaction of cyanuric chloride and N-amino ethylpiperazine/APP (TA-IFR/APP)**	25	N:nN 2:1	44	241	77.2	35	3.2	4.29	24.5	V-1	[31]
	―	―	62	1221	265	35	6	―	19	NR	[182]
**Triazine-based IFR and APP (TAandAPP-IFR)**	10	N:P ―	44	313	235	35	6	3.12	26	NR	[182]
**Triazine-based IFR and APP (TAandAPP-IFR)**	15	N:P ―	45	148	191	35	6	8.30	29	NR	[182]
**Triazine-based IFR and APP (TAandAPP-IFR)**	20	N:P ―	43	115	153	35	6	12.75	31	V-0	[182]
	―	―	45	1269	146.35	50	3	―	17.5	NR	[70]
**Piperazine-based IFR: Piperazine spirocyclic phosphoramidate/APP (PI-IFR/APP)**	30	N:nN 2:1	20	189.2	74.85	50	3	5.82	33.1	V-1	[70]
	―	―	42	1025	137.7	35	4	―	―	―	[74]
**Nitrogen-based FR: compound containing nitrogen (27.5 wt.%) and Phosphorus (15.6 wt.%)/Fumed silica (NP-FR/SiO_2_)**	25	N:nN 49:1	25	124	35.1	35	4	19.30	38	V-0	[74]
**Nitrogen-based FR: compound containing nitrogen (27.5 wt.%) and Phosphorus** **(15.6 wt.%)/Fumed silica (NP-FR/SiO_2_)**	25	N:nN 7.3:1	17	341	87.9	35	4	1.90	27	NR	[74]
	―	―	30	1093	108.2	50	3	―	18	NR	[75]
**Nitrogen-based IFR: Poly (diallyldimethylammonium) and polyphosphate polyelectrolyte complexe/Polyamide-6 (N-IFR/PA6)**	25	N:nN 4:1	17	295.2	80.5	50	3	2.81	27.3	V-1	[75]
	―	―	25	874.1	89.3	50	3	―	18	NR	[76]
**Nitrogen-based IFR: compound containing nitrogen (23%) and Phosphorus (21%)/Hollow glass microsphere (N-IFR/HGM)**	25	N:nN 49:1	16	93.8	74.4	50	3	7.15	34.5	V-0	[76]
**Nitrogen (23%) and Phosphorus (21%)-based intumescent flame retardant/Hollow glass microsphere (N-IFR/HGM)**	25	N:nN 24:1	17	78.8	68	50	3	9.90	36.5	V-0	[76]
**Nitrogen-based IFR: compound containing nitrogen (23%) and Phosphorus (21%)/HGM (N-IFR/HGM)**	25	N:nN 11.5:1	12	61.6	74.2	50	3	8.19	35.5	V-0	[76]
**Nitrogen-based IFR: compound containing nitrogen (23%) and Phosphorus (21%)/HGM (N-IFR/HGM)**	25	N:nN 5.25:1	13	81.6	72.5	50	3	6.86	34.5	V-0	[76]
	―	―	76	455	102	35	3	―	17	NR	[183]
**ATH/APP**	30	M:P 1:1	40	210	75	35	3	1.55	24	V-1	[183]
**4,4′-diphenylmethane diisocyanateandmelamine co-microencapsulated ATH and APP** **(mc-(ATHandAPP))**	30	M:P 1:1	75	120	53	35	3	7.20	25.5	V-0	[183]
	―	―	32	1470	175	50	4	―	18	―	[81]
**ATH/Glass Bubble (ATH/GB)**	60	M:M 11:1	31	212	53	50	4	22.17	25	―	[81]
**ATH/GB**	60	M:M 5:1	36	190	49	50	4	31.08	23.4	―	[81]
**ATH/GB/Octacedylamine modified ZrP (ATH/GB/m-ZrP)**	60	M:M:M 4.7:1:0.3	24	136	90	50	4	15.76	24	―	[81]
**ATH/GB/Octacedylamine modified ZrP (ATH/GB/m-ZrP)**	60	M:M:M 4.4:1:0.6	24	152	91	50	4	13.94	23.2	―	[81]
**ATH/GB/Octacedylamine modified ZrP (ATH/GB/m-ZrP)**	60	M:M:M 4.1:1:0.9	21	189	98	50	4	9.11	22.8	―	[81]
	―	―	37	1425	121.4	50	3	―	17.3	NR	[80]
**ATH/Cetyltrimethyl ammonium bromide modified Fe MMT (ATH/m-MMT)**	50	M:M 49:1	48	482	95.1	50	3	4.89	25.5	NR	[80]
**ATH/Cetyltrimethyl ammonium bromide modified Fe MMT (ATH/m-MMT)**	50	M:M 15.6:1	49	412	90.9	50	3	6.11	27.4	V-1	[80]
**ATH/Cetyltrimethyl ammonium bromide modified Fe MMT (ATH/m-MMT)**	50	M:M 9:1	53	329	89	50	3	8.46	29	V-0	[80]
	―	―	26	1967	112	50	3	―	―	―	[82]
**ATH/Styrene-co-vinylbenzyl chloride modified MMT (ATH/m-MMT)**	23	M:M 6.6:1	21	677	84	50	3	3.12	―	―	[82]
**ATH/Styrene-co-vinylbenzyl chloride modified MMT (ATH/m-MMT)**	30	M:M 2:1	20	592	77	50	3	3.71	―	―	[82]
**ATH/Styrene-co-vinylbenzyl chloride modified MMT (ATH/m-MMT)**	37	M:M 1.17:1	18	536	74	50	3	3.84	―	―	[82]
	―	―	24	687	119	25	3	―	17.8	―	[146]
**MDH/APP/PER/MEL**	44.2	M:nM:nM:nM 1.3:1:0.6:0.56	43	121	58.2	25	3	20.79	28	―	[146]
**MDH/APP/PER/MEL**	50	M:nM:nM:nM 2.1:1:0.6:0.56	43	121	54.5	25	3	22.21	28.8	―	[146]
**MDH/APP/PER/MEL**	54.4	M:nM:nM:nM 3:1:0.6:0.56	44	104	53.6	25	3	26.88	30.2	―	[146]
	―	―	166	412	105	50	3	―	17.8		[146]
**MDH/APP/PER/MEL**	44.2	M:nM:nM:nM 1.3:1:0.6:0.56	217	68.8	39.3	50	3	20.91	28	―	[146]
**MDH/APP/PER/MEL**	50	M:nM:nM:nM 2.1:1:0.6:0.56	220	57.3	36.4	50	3	27.48	28.8	―	[146]
**MDH/APP/PER/MEL**	54.4	M:nM:nM:nM 3:1:0.6:0.56	232	54.3	31.2	50	3	35.68	30.2	―	[146]
	―	―	26	1967	112	50	3	―	―	―	[82]
**MDH/Styrene-co-vinylbenzyl chloride modified MMT (MDH/m-MMT)**	37	M:M 1.17:1	24	476	70	50	3	6.10	―	―	[82]
	―	―	30	1684	89	50	3	―	―	―	[82]
**MDH/Styrene-co-vinylbenzyl chloride modified MMT (MDH/m-MMT)**	40	M:M 3:1	24	471	80	50	3	3.18	―	―	[82]
**MDH/Styrene-co-vinylbenzyl chloride modified MMT (MDH/m-MMT)**	50	M:M 4:1	23	385	69	50	3	4.32	―	―	[82]
**MDH/Styrene-co-vinylbenzyl chloride modified MMT (MDH/m-MMT)**	60	M:M 5:1	22	304	59	50	3	6.12	―	―	[82]
	―	―	38	1425	121.4	50	3	―	17.5	NR	[85]
**MDH/Cetyltrimeyhyl ammonium bromide modified Fe MMT (MDH/m-MMT)**	40	M:M 39:1	52	422	98.1	50	3	5.71	24.9	NR	[85]
**MDH/Cetyltrimeyhyl ammonium bromide modified Fe MMT (MDH/m-MMT)**	40	M:M 12.3:1	56	378	97.5	50	3	6.91	26.5	NR	[85]
**MDH/Cetyltrimeyhyl ammonium bromide modified Fe MMT (MDH/m-MMT)**	40	M:M 7:1	63	329	87.9	50	3	9.91	28.1	V-1	[85]
	―	―	71	2283	218	35	1	―	―	―	[84]
**MDH/4,4′-bis (acrylamido) diphenylsulfone crosslinked N-(4-methyl phenyl) acrylamide monomer (MDH/Cobalt chelate)**	50	M:M 9:1	72	619	306	35	1	2.66	―	―	[84]
**MDH/Cobalt chelate**	50	M:M 4:1	63	618	277	35	1	2.57	―	―	[84]
**MDH/Cobalt chelate**	50	M:M 2.3:1	53	776	236	35	1	2.02	―	―	[84]
**MDH/Cobalt chelate**	50	M:M 1.5:1	56	780	222	35	1	2.26	―	―	[84]
	―	―	37	584	75.6	50	3	―	―	―	[87]
**Cetyltrimethylammonium bromide modified MMT/SEP (m-MMT/SEP)**	5	M:M 1:1	62	417	63.7	50	3	2.78	―	―	[87]
**MDH/Organically treated SEP** **(MDH/m-SEP)**	15	M:M 2:1	29	325	62.1	50	3	1.71	―	―	[87]
**MDH/Organically treated SEP** **(MDH/m-SEP)**	20	M:M 3:1	26	205	53.5	50	3	2.82	―	―	[87]
**MDH/cetyltrimethylammonium bromide modified MMT/SEP (MDH/m-MMT/SEP)**	15	M:M:M 4:1:1	54	246	56.3	50	3	4.65	―	―	[87]
**MDH/cetyltrimethylammonium bromide modified MMT/SEP (MDH/m-MMT/SEP)**	20	M:M:M 4:1:1	50	209	50.1	50	3	5.69	―	―	[87]
	―	―	14	1104	106	35	0.4	―	―	―	[24]
**MMT/APP**	10	M:nM 1.5:1	28	764	64	35	0.4	4.78	―	―	[24]
**MMT/APP**	10	M:nM 4:1	29	751	62	35	0.4	5.20	―	―	[24]
**Modified MMT/APP (m-MMT/APP)**	10	M:nM 1.5:1	30	599	57	35	0.4	7.34	―	―	[24]
**Modified MMT/APP (m-MMT/APP)**	10	M:nM 4:1	31	575	56	35	0.4	8.04	―	―	[24]
	―	―	25	981	147	50	―	―	17.6	NR	[30]
**Polysiloxane based FR/APP (Si-FR/APP)**	25	M:P 1:1	14	277	97	50	―	3.00	28.9	V-0	[30]
	―	―	38	1284	241	50	6	―	―	―	[108]
**Ni_2_O_3_/AC**	15	M:C 1:1	18	385	132	50	6	2.88	―	―	[108]
	―	―	47	1933	176	50	5	―	―	―	[189]
**SEP/MWCNT**	12	M:nM 5:1	32	355	241	50	5	2.70	―	―	[189]
	―	―	62	1378	332	35	―	―	―	―	[190]
**Silicon/Stannous chloride (Si/SnCl_2_)**	5	M:M 1.5:1	91	860.1	193.7	35	―	4.03	―	―	[190]
	―	―	88	565.9	71.9	35	3	―	―	―	[105]
**C20A/TiO_2_**	5.5	M:M 10:1	83	458.9	78.1	35	3	1.07	20	―	[105]
**Ethylene glycol methacrylate phosphate modified C20A/TiO_2_ (m-C20A/TiO_2_)**	5.5	M:M 10:1	78	498.2	75.2	35	3	0.96	19	―	[105]
**Ethylene glycol methacrylate phosphate modified C20A/TiO_2_ (m-C20A/TiO_2_)**	10.5	M:M 20:1	61	424.7	74.8	35	3	0.88	20	―	[105]
	―	―	38	1284	214	50	6	―	18.2	―	[133]
**CF/MWCNT**	10	C:C 1:1	25	364	194	50	6	2.55	25.8	―	[133]
	―	―	30	1261	208	50	6	―	18	―	[131]
**CB/MWCNT**	4	C:C 3:1	26	402	187	50	6	3.02	23.8	―	[131]
**CB/MWCNT**	6	C:C 5:1	27	353	185	50	6	3.61	26.5	―	[131]
**CB/MWCNT**	8	C:C 1.6:1	25	314	180	50	6	3.86	27.6	―	[131]
	―	―	35	1212	198	50	6	―	18.2	―	[134]
**CB/CF**	8	C:C 1.66:1	27	361	166	50	6	3.08	25.7	―	[134]
	―	―	38	1284	241	50	6	―	―	―	[108]
**AC/Ni_2_O_3_**	15	C:M 1:1	18	385	132	50	6	2.88	―	―	[108]
	―	―	166	412	105	25	3	―	―	―	[145]
**PER/MEL/APP**	25.3	C:nC:nC 1.07:1:0.53	170	140	61.1	25	3	5.17	―	―	[145]
	―	―	24	687	119	50	3	―	―	―	[145]
**PER/MEL/APP**	25.3	C:nC:nC 1.07:1:0.53	32	198	79	50	3	6.96	―	―	[145]
	―	―	46	1541	90	35	3	―	―	―	[135]
**Cyclodextrin nanosponge/Triethylphosphate (CD/TEP)**	10	Bio:nBio 2.3:1	30	1529	93	35	3	0.63	―	―	[135]
**CD/TEP**	15	Bio:nBio 2:1	26	839	90	35	3	1.03	―	―	[135]
**CD/APP**	15	Bio:P 1:1	24	910	89	35	3	0.89	―	―	[135]
	―	―	49	1350	87.3	35	3	―	17.5	―	[136]
**Phosphorus and Nitrogen elements modified lignin/Nickel acetate (m-lig/Ni(Ac)_2_)**	20	Bio:nBio 9:1	31	330	69.5	35	3	3.25	26	―	[136]
**Phosphorus and Nitrogen elements modified lignin/Cobalt acetate (m-lig/Co(Ac)_2_)**	20	Bio:nBio 9:1	37	362	72.8	35	3	3.37	24.5	―	[136]
**Phosphorus and Nitrogen elements modified lignin/Zinc acetate (m-lig/Zn(Ac)_2_)**	20	Bio:nBio 9:1	38	368	73.5	35	3	3.37	23	―	[136]
